# Homotypic clustering of L1 and B1/Alu repeats compartmentalizes the 3D genome

**DOI:** 10.1038/s41422-020-00466-6

**Published:** 2021-01-29

**Authors:** J. Yuyang Lu, Lei Chang, Tong Li, Ting Wang, Yafei Yin, Ge Zhan, Xue Han, Ke Zhang, Yibing Tao, Michelle Percharde, Liang Wang, Qi Peng, Pixi Yan, Hui Zhang, Xianju Bi, Wen Shao, Yantao Hong, Zhongyang Wu, Runze Ma, Peizhe Wang, Wenzhi Li, Jing Zhang, Zai Chang, Yingping Hou, Bing Zhu, Miguel Ramalho-Santos, Pilong Li, Wei Xie, Jie Na, Yujie Sun, Xiaohua Shen

**Affiliations:** 1 Tsinghua-Peking Joint Center for Life Sciences, School of Medicine and School of Life Sciences, Tsinghua University, Beijing, 100084 China; 2 State Key Laboratory of Membrane Biology, Biomedical Pioneering Innovation Center (BIOPIC), School of Life Sciences, and College of Future Technology, Peking University, Beijing, 100871 China; 3 Bioland Laboratory (Guangzhou Regenerative Medicine and Health Guangdong Laboratory), Guangzhou, Guangdong, 510005 China; 4 MRC London Institute of Medical Sciences (LMS), London, W120NN UK; 5 Institute of Clinical Sciences (ICS), Faculty of Medicine, Imperial College London, London, W120NN UK; 6 National Laboratory of Biomacromolecules, CAS Center for Excellence in Biomacromolecules, Institute of Biophysics, Chinese Academy of Sciences, Beijing, 100101 China; 7 Lunenfeld-Tanenbaum Research Institute, University of Toronto, Toronto, Ontario M5T 3H7 Canada

**Keywords:** Chromatin structure, Transposition, Nuclear organization

## Abstract

Organization of the genome into euchromatin and heterochromatin appears to be evolutionarily conserved and relatively stable during lineage differentiation. In an effort to unravel the basic principle underlying genome folding, here we focus on the genome itself and report a fundamental role for L1 (LINE1 or LINE-1) and B1/Alu retrotransposons, the most abundant subclasses of repetitive sequences, in chromatin compartmentalization. We find that homotypic clustering of L1 and B1/Alu demarcates the genome into grossly exclusive domains, and characterizes and predicts Hi-C compartments. Spatial segregation of L1-rich sequences in the nuclear and nucleolar peripheries and B1/Alu-rich sequences in the nuclear interior is conserved in mouse and human cells and occurs dynamically during the cell cycle. In addition, de novo establishment of L1 and B1 nuclear segregation is coincident with the formation of higher-order chromatin structures during early embryogenesis and appears to be critically regulated by L1 and B1 transcripts. Importantly, depletion of L1 transcripts in embryonic stem cells drastically weakens homotypic repeat contacts and compartmental strength, and disrupts the nuclear segregation of L1- or B1-rich chromosomal sequences at genome-wide and individual sites. Mechanistically, nuclear co-localization and liquid droplet formation of L1 repeat DNA and RNA with heterochromatin protein HP1α suggest a phase-separation mechanism by which L1 promotes heterochromatin compartmentalization. Taken together, we propose a genetically encoded model in which L1 and B1/Alu repeats blueprint chromatin macrostructure. Our model explains the robustness of genome folding into a common conserved core, on which dynamic gene regulation is overlaid across cells.

## Introduction

The mammalian genomic DNA that is roughly 2 meters long in a cell is folded extensively in order to fit the size of the nucleus with a diameter of ~5–10 μm.^1^ Microscopic and 3C-based approaches reveal a hierarchical organization of the genome.^2–5^ At the megabase scale, chromatin is subdivided into two spatially segregated compartments, arbitrarily labeled as A and B, with distinct transcriptional activity and histone modification as well as other features such as CpG frequency and DNA replication timing.^6–10^ The euchromatic A compartment adopts a central position, whereas the heterochromatic B compartment moves towards the nuclear periphery and nucleolar regions.^11^ This nuclear organization appears to be conserved from ciliates to humans and has been maintained in eukaryotes over 500 million years of evolution.^12^ Within compartments at the kilobase-to-megabase scale, chromatin is organized in topologically associated domains (TADs), which serve as functional platforms for physical interactions between co-regulated genes and regulatory elements.^13^ At a finer scale, TADs are divided into smaller loop domains, in which distal regulatory elements such as enhancers come into direct contact with their target genes via chromatin loops.^14^ Intriguingly, most A/B compartments and TADs are relatively stable in different mouse and human cell types (Supplementary information, Text S1), whereas sub-TAD loops and a small fraction of lineage-specific regions with less pronounced compartment associations tend to be more variable for differential gene expression during cell-fate transition.^13,15–19^

Evidence suggests that compartments and TADs may be formed by distinct mechanisms. TADs are thought to be formed by active extrusion of chromatin loops by the ring-shaped cohesin complex, which co-localizes with the insulator protein CTCF at the boundaries and anchor regions of contact domains and loops.^20,21^ Depletion of CTCF disrupted TAD boundaries but failed to impact compartmentalization, whereas cohesion loss made TADs disappear but increased compartmentalization, although both eliminated sub-TAD loop contacts.^22–30^ These results indicate that compartmentalization of mammalian chromosomes emerges independently of proper insulation of TADs. A few mechanisms have been proposed for compartmentalization, such as anchoring heterochromatin to the nuclear lamina,^31–35^ preferential attraction of chromatin harboring similar histone modifications and regulators,^4,36–39^ and hypothetical models involving pairing of homologous sequences mediated by active transcription and phase separation of block copolymers.^36,40–44^ Although lamin-associated domains (LADs) contribute to a basal chromosome architecture, a large body of work has demonstrated a secondary role for lamina scaffolding in compartmental segregation of heterochromatin and euchromatin.^31–35,45–49^ In vitro assembled nucleosomal arrays harboring histone H3 lysine 9 di- and tri-methylation (H3K9me2 and H3K9me3) marks undergo phase separation with heterochromatin protein 1 (HP1) and associated proteins to form macromolecule-enriched liquid droplets, reminiscent of heterochromatin.^38^ However, the role of histone modifications in regulating compartmentalization in vivo remains uncertain. Taken SUV39H H3K9 methyltransferases for example, *SUV39H* double-null cells still exhibit DAPI-dense heterochromatin foci despite the loss of pericentric H3K9me3 marks;^39^ and double knockout mice of SUV39H survive at birth with abnormalities.^48^ A phase-separation model of block copolymers with similar activity appears attractive in explaining compartmental formation.^12,34,40–44^ However, this hypothesis remains inconclusive, owing to a large void of identification and experimental validation of the molecular drivers that underlie compartmental segregation of euchromatin and heterochromatin.

Repetitive elements comprise more than half of human and mouse genomes.^50,51^ Once regarded as genomic parasites,^52^ retrotransposons have been recently implicated in playing active roles in re-wiring the genome and gene expression programs in diverse biological processes.^53–61^ Long and short interspersed nuclear elements (LINEs and SINEs, respectively) are the two predominant subfamilies of retrotransposons in most mammals.^62^ L1 (also named as LINE1 or LINE-1) is the most abundant subclass of all repeats, making up to 19% and 17% (0.9–1.0 million copies) of the genome in mouse and human, respectively.^63^ B1 in mouse and its closely related, primate-specific Alu elements in human are the most abundant subclass of SINEs, constituting 3%–11% (0.6–1.3 million copies) of mouse and human genomes.^64,65^ L1 and B1/Alu have distinct nucleotide compositions and sequence lengths. L1 elements are 6–7 kb long and AT-rich, while Alu elements are ~300 bp long and rich in G and C nucleotides.^66^ Analysis of metaphase chromosome banding showed roughly inverse distributions of L1 and Alu elements in chromosomal regions with distinct biochemical properties.^45,67,68^ Initial studies suggested that Alu/B1 elements appear to be enriched in gene-rich, euchromatic A compartments, whereas L1 elements tend to be enriched in gene-poor, heterochromatic B compartments that interact with lamina-associated domains.^35,45,47,69–71^ However, evidence to pinpoint a role for L1 and B1/Alu repeats in organizing the genome has to our knowledge not been reported, albeit fragmented information about their localizations in scattered reports (Supplementary information, Text S1). Systematic mapping and visualization of L1 and B1/Alu distributions are still lacking.

We have postulated that the primary DNA sequences, particularly abundant repetitive elements embedded in the genome, may instruct genome folding.^61^ Here, we report that L1 and B1/Alu repeats tend to cluster with sequences from their own repeat subfamily and form grossly exclusive domains in the nuclear space, which efficiently explains and predicts the compartmental organization revealed by Hi-C. The segregated pattern of L1-rich sequences in the nuclear and nucleolar peripheries and B1/Alu-rich sequences in the nuclear interior is highly conserved across a variety of mouse and human cells, and re-occurs during the cell cycle. In addition, de novo establishment of nuclear segregation of L1- and B1-rich compartments is coincident with the formation of higher-order chromatin structures during early embryogenesis, and appears to be critically regulated by L1 and B1 repeat RNA. Importantly, depletion of L1 RNA in mouse embryonic stem cells (mESCs) significantly weakens spatial contacts of homotypic repeat DNA, disrupts the nuclear localization and segregation of L1- or B1-rich chromosomal sequences, and leads to attenuated compartmentalization of the higher-order chromatin structure. Moreover, we show that recombinant HP1α is able to bind RNA and to phase separate in the presence of RNA or DNA in vitro. Genome-wide co-localization of L1 and HP1α renders these repeat DNA and RNA sequences an advantage in promoting HP1α phase separation in heterochromatin contexts. Altogether, our findings suggest a genetically encoded mechanism by which L1 and B1/Alu repeats organize chromatin macrostructure at the compartmental level, providing an important clue to the conservation and robustness of the higher-order chromatin structure across mouse and human.

## Results

### L1 and B1/Alu distributions correlate with global compartmentalization in mouse and human

**Fig. 1:**
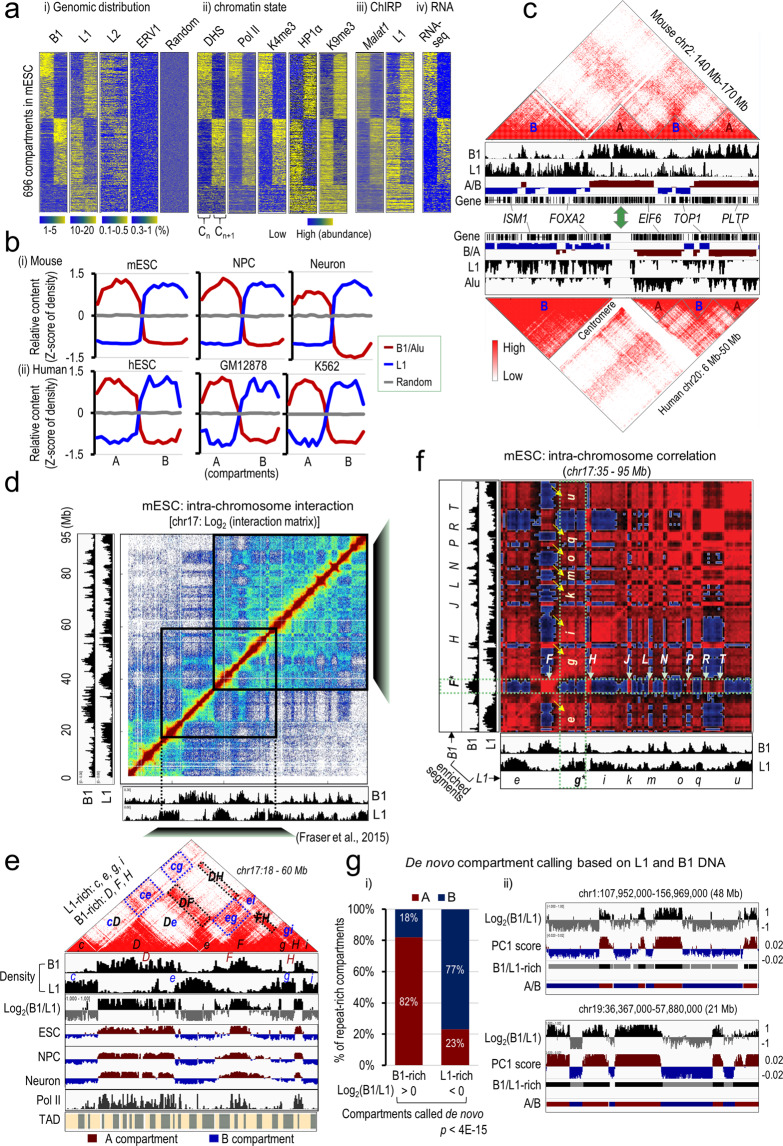
B1- and L1-rich genomic regions homotypically interact, characterize and predict Hi-C compartments. **a** Heatmaps of the distribution densities of B1, L1, L2, and ERV1 repeats and random genomic regions (panel (i)), DNase I hypersensitive sites (DHS) and ChIP-seq signals of Pol II, H3K4me3, HP1α, and H3K9me3 (panel (ii)), ChIRP-seq signals of *Malat1* and L1 RNA (panel (iii)), and RNA-seq (panel (iv)) in mESCs across two adjacent compartments (C_n_, C_n+1_). All signals in 696 compartments annotated in mESCs were sorted according to the B1 distributions shown in panel (i). **b** Relative contents of L1 and B1/Alu repeats across the A and B compartments annotated in various cell types in mouse (top) and human (bottom). Random genomic regions serve as the negative control. **c** Genome browser shots showing conserved domain structures as indicated by heatmaps of the Hi-C contact matrix over a syntenic region in mouse (top) and human (bottom) ESCs. The B1/Alu and L1 repeat densities, the A/B compartments are shown by eigenvalues of the Hi-C contact matrix, and Refseq gene annotations are shown underneath each heatmap. **d** Heatmap of normalized interaction frequencies at 100-kb resolution on chromosome 17 in mESCs. Genomic distributions and densities of B1 and L1 repeats are shown in the left and bottom tracks. **e** A zoomed-in view of the interaction matrix of the genomic region from 18 to 60 Mb on mouse chr17 (40-kb resolution). Under the heatmap, we show sequentially genomic distributions and densities of B1 and L1 repeats (in 10-kb bin), log_2_ ratio of B1 to L1 density, eigenvalues of the Hi-C matrix representing A/B compartments from mouse mESCs, neural progenitor cells (NPC) and neurons, and Pol II ChIP-seq signals and annotated TADs in mESCs. B1-rich regions are arbitrarily labeled as *D, F*, and *H* in uppercase. L1-rich regions are labeled as *c, e, g*, and *i* in lowercase. Some strong homotypic interactions between compartments rich in the same repeat subfamily (for example, between the B1-rich regions *DF*, *DE* and *FH*, and between the L1-rich regions *ce* and *cg*), are highlighted by dotted boxes. **f** Correlation heatmap showing Pearsonʼs correlation coefficients of the interaction frequencies of any two paired regions in a sub-region on chr17 (500-kb resolution). B1-rich regions are labeled in uppercase as *F, H … R, T*. L1-rich regions are labeled in lowercase as *e, g … q, u*. Dotted boxes (in red) and arrows highlight positive correlations of the anchor region *F* (indicated by *) with other B1-rich genomic regions (horizontal), and of the anchor region *g* (indicated by *) with other L1-rich genomic regions (vertical). **g** De novo compartment calling based on L1 and B1 DNA sequences. Panel (i) shows the percentage of L1- or B1-rich compartments overlapped with A or B compartments identified by Hi-C. Panel (ii) shows representative genomic regions with ratio of B1 to L1 in log_2_ scale and PC1 score of Hi-C interaction matrix.

We analyzed the genomic positions of the major repeat subfamilies in mouse and observed positive correlations within L1 or SINE B1 subfamilies, but strong inverse correlations between them (Supplementary information, Fig. S1a). This suggests that L1 and B1 elements tend to be positioned away from each other in the genome, while repeats from the same subfamily tend to be clustered. The non-random positioning of repeat sequences in the genome prompted us to examine their relative distributions in high-order chromatin structures. We first analyzed the published Hi-C data from mESCs.^72^ Dense L1 and B1 repeats appear to be enriched in distinct compartments across the mouse genome, and within a compartment they are evenly distributed without obvious bias towards the boundary (Fig. 1a, b). L2 repeats show weak enrichments in B1-rich compartments, whereas other types of retrotransposons such as ERV1 and ERVK tend to be randomly distributed (Fig. 1a and Supplementary information, Fig. S1b). The compartments marked by B1 repeats show enrichment of active histone marks (H3K4me3, H3K9ac, H3K27ac, H3K36me3), strong binding of RNA polymerase II (Pol II), and high levels of chromatin accessibility and transcription activity. In contrast, the compartments marked by L1 repeats show signatures of heterochromatin, including enrichment of the repressive H3K9me2 and H3K9me3 marks, and strong binding of heterochromatin proteins such as HP1α and the nuclear corepressor KRAB-associated protein-1 (KAP1 or TRIM28) (Fig. 1a and Supplementary information, Fig. S1b).

We then performed a quantitative sequence analysis of annotated A/B compartments in six distinct mouse and human cell types.^20,72^ All cells exhibit consistently high levels of SINE repeats (including B1, B2, B4 in mouse and Alu in human) in the A compartments and L1 repeats (including truncated or intact, and evolutionary old or young L1s) in the B compartments (Fig. 1b and Supplementary information, Fig. S1c–e). In contrast, L2 and ERV1 repeats fail to show consistent enrichments across mouse and human. In addition, unsupervised clustering revealed that the genomic positions of A/B compartments are highly similar across six cell types, with an average Spearman correlation coefficient > 0.73 within species and > 0.52 between species (Supplementary information, Fig. S1f). Compared to other subclass repeats, L1 and B1/Alu are most strongly related to the high-order chromatin structures, and their distributions appear to be conserved in homologous regions of the mouse and human genomes (Supplementary information, Fig. S1f). For example, a region in mouse chromosome 2 (chr2: 140–170 Mb) and its syntenic region in human chromosome 20 (chr20: 6–50 Mb) show similar patterns of Hi-C contact probabilities, and gene and repeat compositions and distributions in the corresponding A and B compartments along the DNA sequences (Fig. 1c).

We further analyzed the published datasets of higher-order chromatin interactions in 21 primary human tissues and cell lines.^17^ On the basis of the PC1 values of a principal components analysis on the Hi-C correlation matrix reported by Schmitt et al.,^17^ we found that A/B-compartmental associations are highly correlated across all 21 examined samples with correlation coefficients ranging 0.47–0.99 and a median value of 0.79 (Supplementary information, Fig. S2a). The degree of compartmental conservation is highly significant (*P* < 2.2e-16), as ~80% of the genome shows consistent compartmental labeling in at least 16 samples and ~40% is invariant in all 21 samples, in contrast to 7% and 0% to be expected by chance, respectively (Supplementary information, Fig. S2b, c). Most of compartmental switches that account for 20% of the genome occur in one or few (≤ 5) samples with less pronounced compartmental labeling (gray highlighted regions with low absolute values of PC1 in Supplementary information, Fig. S2d; see also Supplementary information, Text S1). Thus, despite some switching events occurring in individual cells, global compartmentalization is rather stable. Consistently, the genomic regions with conserved A or B compartments across samples exhibit significantly higher levels of Alu or L1 repeats, respectively (Supplementary information, Fig. S2e). Altogether, these results indicate that co-segregation of B1/Alu and L1 repeats with the A and B compartments appears to be stable in different cell types in mouse and human.

### Homotypic clustering of L1 and B1 repeats characterizes and predicts compartmental organization

To have a close look at repeat distribution and the higher-order chromatin structure, we took mouse chromosome 17 (chr17, 95 Mb in length) as an example to overlay L1 and B1 features on the Hi-C interaction matrix of mESCs. Interestingly, the plaid pattern of enriched and depleted interaction blocks in the Hi-C map is largely correlated with the compositions and distributions of B1 and L1 along the whole chr17 (Fig. 1d). In a 42-Mb region of chr17, four L1-rich compartments (denoted by *c*, *e, g* and *i*) and three B1-rich compartments (denoted by *D*, *F* and *H*) are alternately positioned along the linear DNA sequence (Fig. 1e). Strong interactions were observed within L1-rich compartments (represented by *ce*, *cg, eg, ei* and *gi*, dotted boxes) or B1-rich compartments (represented by *DF*, *DH* and *FH*, dotted boxes), but not between these two compartments (Fig. 1e). The interaction frequencies between *D* and *F* (*DF*) or between *c* and *e* (*ce*) are much stronger than those of *D* or *F* with *c* or *e* (*cD*, *De*, *eF*), despite the fact that these regions are closer in the linear sequence. Note that L1- or B1-rich segments often span several adjacent TADs (Fig. 1e and Supplementary information, Fig. S2f), consistent with the findings that TADs are smaller, structural units of compartments.^73,74^ L1 and B1 compositions within a TAD also exhibit strong anti-correlations (Pearson correlation coefficient <−0.75) across 2200 annotated TADs in mESCs (Supplementary information, Fig. S2g). This observation is consistent with repeat analyses at the genome-wide and compartmental levels (Fig. 1a and Supplementary information, Fig. S1a), illustrating a mutually exclusive distribution of L1 or B1-rich sequences along the genome.

Conversion of Hi-C contact frequencies into Pearson correlation coefficients sharpened our view of the long-range chromatin interactions (Fig. 1f). By visual inspection, we found that the plaid pattern of the Hi-C correlation map precisely matches the distribution and interaction status of L1 and B1. L1-rich or B1-rich regions show strong enrichment of contacts with regions containing the same repeat type (red blocks in Fig. 1f). We refer to these as homotypic contacts. Contacts between regions containing the other repeat type (heterotypic interactions) are strongly depleted (blue blocks in Fig. 1f). For example, in one region of chr17 (35 to 95 Mb), L1-rich segments (from *e* to *u*) and B1-rich segments (from *F* to *T*) exhibit high frequencies of homotypic contacts (Fig. 1f, highlighted by arrows), but strong depletion of heterotypic contacts. Similarly, homotypic contacts between L1-rich regions or B1-rich regions were also observed between chromosomes, as illustrated by chromosomes 17 and 19 (Supplementary information, Fig. S2h). These results indicate that genomic regions containing B1 or L1 repeats tend to interact with genomic regions containing repeat sequences from similar subfamilies, but not from different subfamilies, regardless of linear proximity, which characterizes the organization at intra- and inter-chromosomal levels.

Next, we sought to predict compartmental organization based on repeat distributions. We used the criterion of log_2_ ratio of B1 to L1 density [log_2_(B1/L1)] larger or smaller than 0 for B1-rich or L1-rich compartments, respectively. About 540 B1-rich and 648 L1-rich compartments were identified with a median size of 1.2 Mb across the mouse genome (Supplementary information, Table S1). The numbers and sizes of these B1- and L1-rich compartments called de novo are comparable to those of A and B compartments annotated by Hi-C in mESCs (366 and 364, respectively, with a median size of 1.9 Mb). Importantly, 82% of B1-rich compartments and 77% of L1-rich compartments are overlapped with annotated A or B compartments, respectively (Fig. 1g and Supplementary information, Fig. S3). Only 18% to 23% of compartments show inconsistent labeling between our prediction and Hi-C. We then analyzed genomic features in these ‘falsely’ labeled regions. Intriguingly, L1-rich regions that fall into Hi-C-annotated A compartments (designated as ‘L1.A’) still exhibit a high level of heterochromatic H3K9me3 mark and low levels of chromatin accessibility and gene expression, and contain genes enriched in specialized functions such as responses to pheromone and immunoglobulin and synapse (Supplementary information, Fig. S4a–c). Similarly, B1-rich regions that fall into Hi-C-annotated B compartments (designated as ‘B1.B’) exhibit high levels of chromatin accessibility and gene expression but low H3K9me3 binding (Supplementary information, Fig. S4a–c). In addition, L1.A and B1.B regions exhibit significantly less pronounced PC1 values (close to zero) than those consistent regions (B1.A and L1.B) (Supplementary information, Fig. S4a). Thus, a mere usage of B1 to L1 density ratios successfully re-constructs most of A and B compartments annotated by Hi-C, which suggests that the linear genomic DNA repeats contain the macroscopic structural information. Taken together, homotypic clustering of regions rich in B1 or L1 repeats nicely explains and predicts genome organization at the compartmental level.

### Nuclear segregation of L1- and B1/Alu-rich compartments is conserved

**Fig. 2:**
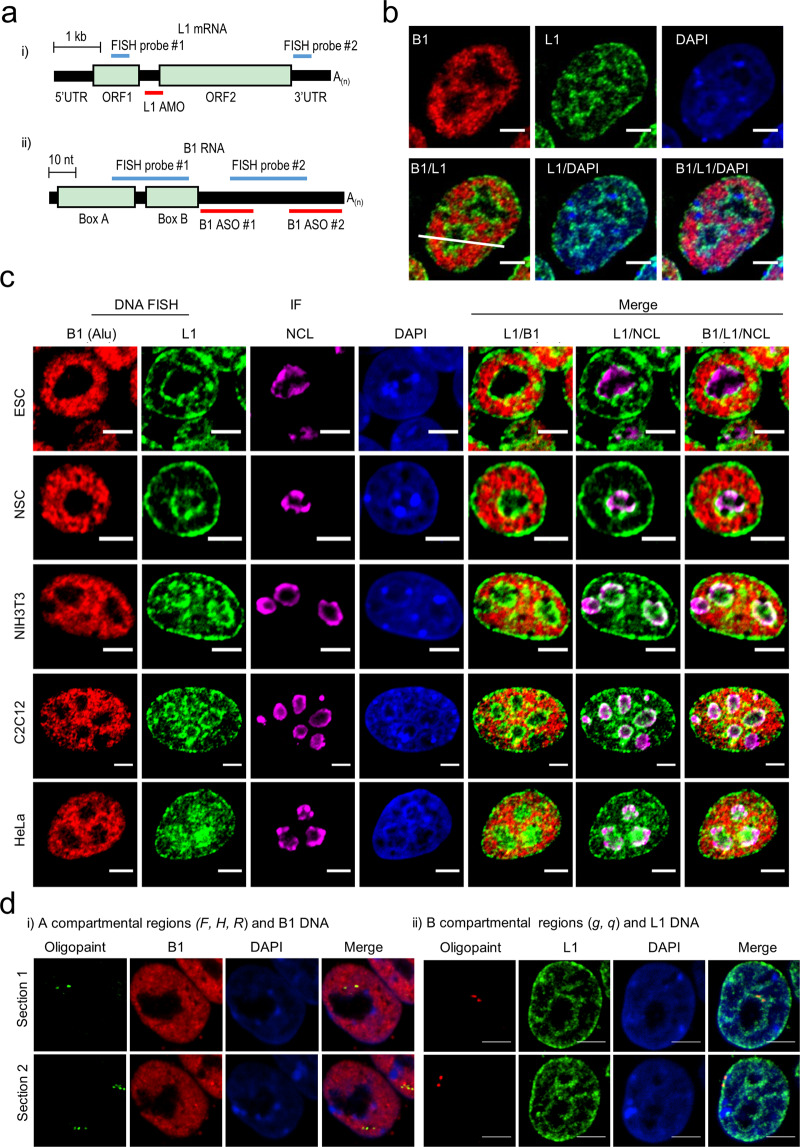
DNA FISH reveals the spatial segregation of L1 and B1 compartments. **a** Schematic illustrations of L1 (panel (i)) and B1 (panel (ii)) RNA targeted by AMO/ASO or DNA FISH probes. **b** Representative images of L1 (green) and B1 (red) repeats revealed by DNA FISH in mESCs. DNA is labeled by DAPI (blue). All scale bars, 5 μm. **c** Representative images of DNA immuno-FISH analysis of L1 (green) and B1 (red) DNA repeats, and NCL protein (purple) in mESCs (*n* = 37), NSC (*n* = 23), NIH3T3 (*n* = 18), C2C12 (*n* = 11) and HeLa cells (*n* = 15). **d** Representative images of Oligopaint DNA FISH of individual sites in A or B compartments. Panel (i), three A-compartmental regions (*F, H, R*) with B1 DNA FISH; panel (ii), two B-compartmental regions (*g, q*) with L1 DNA FISH. See also Supplementary information, Fig. S5.

High-resolution imaging of L1 and B1 distributions in the conventional nucleus remains lacking, despite initial evidence of their differential localization.^45,68^ To visualize their positioning in the nuclear space, we performed dual-color DNA fluorescence in situ hybridization (FISH) using fluorescence-tagged oligonucleotide probes that specifically target the consensus sequences of B1 and L1 elements (Fig. 2a). Strikingly, L1 and B1 exhibit distinct yet complementary nuclear localizations in mESCs (Fig. 2b and Supplementary information, Fig. S5a). B1 DNA shows punctate signals in the nuclear interior. In contrast, L1 DNA exhibits highly organized and concentrated signals that line the periphery of the nucleus and nucleolus. Weak L1 signals were also detected in a few areas of the nuclear interior subregions where B1 signals were absent. Both B1 and L1 signals are absent from DAPI-dense regions, which likely represent satellite repeat-enriched chromocenters.^75^

To confirm the L1 localization at the nucleolar periphery in mESCs, we performed DNA immuno-FISH, using an antibody against the nucleolar marker Nucleolin (NCL). Indeed, L1 signals surround and partially overlap with the ring-shaped signals of NCL at the nucleolar periphery (Fig. 2c). The localization of L1 surrounding the nucleus and nucleolus is consistent with sequencing-based analysis of nucleolus- and lamina-associated domains (NADs and LADs), in which L1-rich sequences are sequestered.^61,69,76,77^ In addition, to further confirm nuclear colocalization of L1-rich sequences in B compartments and B1-rich sequences in A compartments, we performed Oligopaint DNA FISH for five representative loci, each of which ranging from ~100 to 1 Mb was targeted by a set of 500–4500 DNA probes (targeting single-copy sequences) at a density of 200–300 bp per probe. Indeed, three regions (*F, H*, and *R*) annotated in the A compartment are colocalized with B1 FISH signals in the nuclear interior (205 out of 217 nuclei), whereas two B compartment-associated regions (*g* and *q*) are colocalized with L1 FISH signals in either LAD or NAD (248 out of 251 nuclei) (Fig. 2d and Supplementary information, Fig. S5b, c).

Moreover, to ask whether L1 and B1 localizations might vary with cell type, we analyzed four additional cell lines, including mouse neural stem cells (NSC), fibroblasts (NIH3T3), and myoblasts (C2C12), and human cervical cancer cells (HeLa) (Fig. 2c). Similar to mESCs, all these cells show non-overlapping localizations of B1/Alu in the nuclear interior and L1 at the nuclear and nucleolar peripheries. Thus, consistent with Hi-C results, the segregated staining pattern of B1/Alu and L1 further demonstrates that homotypic clustering of similar repeat sequences in the nuclear space divides the nucleus into distinct territories. This pattern is conserved across different cell types in mouse and human.

### Dynamic re-construction of L1 and B1/Alu segregation during the cell cycle

**Fig. 3:**
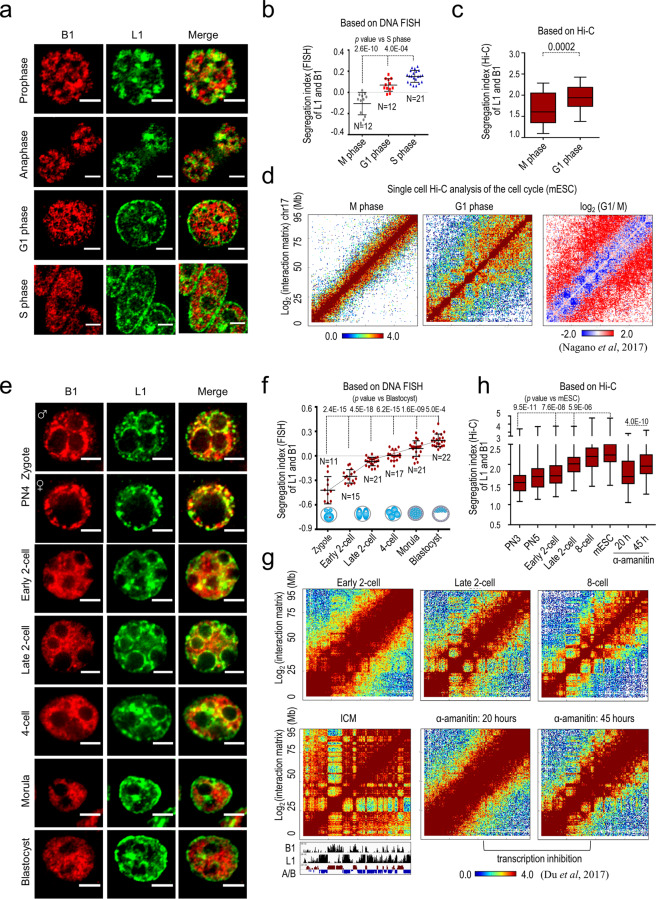
Dynamic segregation of L1 and B1 compartments during the cell cycle and embryonic development. **a**, **b** DNA FISH analysis of synchronized mESCs at different cell cycle stages. Representative images and the scatterplot of the segregation index of L1 and B1 DNA are shown in **a** and **b**, respectively. Data are presented as means ± standard deviation (SD). *n*, number of nuclei analyzed. M phase and G1 phase data are compared to S phase using the two-tailed Student’s *t*-test. *P* values are shown at the top. **c** Boxplot analysis of the ratio of homotypic interactions versus heterotypic interactions for B1 and L1 based on single-cell Hi-C data from mESCs. The *y*-axis shows the segregation index (Hi-C) of L1 and B1, which represents the ratio of average interaction frequency between compartments containing similar repeats (B1.B1 and L1.L1) to that between compartments containing different repeats (B1.L1) for all chromosomes (except X and Y). Larger values indicate a higher degree of homotypic interaction between B1- or L1-rich compartments. *P* values are calculated with the two-tailed Student’s *t-*test. **d** Heatmaps of normalized interaction frequencies at 500-kb resolution on chromosome 17 in mESCs at M (left) and G1 (middle) phase. A comparison of contact frequencies between M and G1 phase [log_2_(G1/M)] for the whole of chromosome 17 is shown on the right. **e**, **f** DNA FISH analysis of early embryos. Representative FISH images and the scatterplot of the segregation index of L1 and B1 DNA are shown in **e** and **f**, respectively. Data are presented as means ± SD (embryos were collected and processed in two independent experiments). *n*, number of nuclei analyzed. Each sample is compared to sample at the blastocyst stage and *P* values are calculated with the two-tailed Student’s *t*-test. Dotted lines show the trend-line. **g** Heatmaps of normalized interaction frequencies at 500-kb resolution on chromosome 17 in mouse embryos at the early 2-cell, late 2-cell, 8-cell, inner cell mass (ICM) stages and also in embryos treated with the transcription inhibitor α-amanitin for 20 and 45 h. Genomic densities of B1 and L1 repeats are shown at the bottom. **h** Boxplots showing the Hi-C segregation index of L1 and B1 in early embryos at various stages. The first six samples show a gradual increase of homotypic versus heterotypic interactions between L1-rich and B1-rich regions from zygotes (including PN3 and PN5 stages) to 8-cell embryos and pluripotent mESCs (in vitro equivalent of the inner cell mass cells of blastocysts). Each sample is compared to mESCs and *P*  values are calculated with the two-tailed Student’s *t-*test. The last two samples show the segregation index for embryos treated with α-amanitin for 20 or 45 h. *P* values are calculated with two-tailed Student’s *t*-test.

We then asked whether the nuclear segregation of L1- and B1-rich compartments could be re-constructed during mitosis when chromatin structure undergoes dynamic reorganization. DNA FISH analysis of synchronous mESCs showed that L1 and B1 localizations change dramatically at different cell cycle stages (Fig. 3a and Supplementary information, Fig. S6a). S-phase cells show non-overlapping and complementary localizations of L1 and B1 repeats (Figs. 2b and 3a). This is similar to the pattern we observed previously in asynchronous mESCs, more than half of which are in the S phase of the cell cycle (Supplementary information, Fig. S6a). However, L1 and B1 DNA signals are mixed on mitotic chromosomes in metaphase (M phase, including prophase and anaphase), when the nuclear membrane and nucleoli are disassembled. As the cell cycle progresses into the G1 phase, L1 and B1 DNA start to segregate again (Fig. 3a and Supplementary information, Fig. S6b). To quantify the degree of segregation, we defined a FISH-based segregation index as the negative value of Pearson’s correlation coefficient of L1 and B1 DNA signals in the nucleus. The FISH segregation index is lowest in M-phase cells, but increases significantly in the G1 phase and peaks in the S phase (Fig. 3b).

To provide molecular evidence for the segregation of repeats during the cell cycle, we analyzed the published Hi-C data from cell-cycle synchronized mESCs and HeLa cells.^78,79^ In both cell types, G1-phase cells exhibit a classic plaid pattern of hierarchical interactions, with enriched and depleted interaction blocks outside of the diagonal region of the Hi-C interaction heatmap (Fig. 3c, d and Supplementary information, Fig. S6c, d). In contrast, M-phase cells exhibit stronger signals along the diagonal, which represent the linearly organized, longitudinally compressed array of consecutive chromatin loops. To quantify this difference, we defined a Hi-C-based segregation index by calculating the ratio of homotypic versus heterotypic interaction frequencies between L1 and B1/Alu subfamilies. Indeed, the Hi-C segregation index is significantly higher in G1-phase cells than in M-phase cells (Fig. 3c and Supplementary information, Fig. S6c). These results indicate that segregation of B1/Alu and L1 repeats is dispersed by mitosis, and is re-established when the higher-order chromatin structure forms during each cell cycle in both mouse and human cells. This finding agrees with previous reports that in metaphase, chromosome folding becomes homogeneous and large megabase-scale A and B compartments are lost, whilst in interphase, chromosomes return to a highly compartmentalized state.^78,79^

### Dynamic establishment of L1 and B1 segregation in early embryogenesis

After fertilization, the chromatin undergoes extensive reprogramming from a markedly relaxed state in zygotes to fully organized structures in blastocysts.^80–82^ We performed a time-course DNA FISH analysis of L1 and B1 in early mouse embryos. During embryonic divisions, L1 and B1 signals are largely overlapping in zygotes, and become progressively more segregated in 2-cell, 4-cell, morula and blastocyst embryos (Fig. 3e, f). Consistently, analysis of the published Hi-C data of early embryos^81^ showed that early 2-cell embryos exhibit prevalent *cis*-chromosomal contacts along the diagonal of the Hi-C interaction map, whereas the plaid patterns of Hi-C interactions become readily detectable in late 2-cell embryos and are fully established in the inner cell mass (ICM) cells of blastocysts (Fig. 3g and Supplementary information, Fig. S7a). Plotting the FISH and Hi-C segregation indexes showed a gradual increase of L1 and B1 segregation along the course of blastocyst development, which reaches the highest level in blastocysts or in mESCs (Fig. 3f, h). We conclude that, in early embryos, compartmentalization of L1- and B1-rich regions appears to be established in a stepwise manner, coincident with de novo establishment of higher-order chromatin structures. Notably, the greatest change (steepest trend-line) of FISH segregation indexes occurs between the zygote and the late 2-cell stage (Fig. 3f), which implies that the initiation of B1 and L1 compartmentalization may coincide with the zygotic genome activation, during which massive transcription switches on.

It was reported that inhibition of Pol II by α-amanitin caused embryonic arrest at the late 2-cell stage,^83^ yet the higher-order chromatin structure could still be established.^80,81^ However, compared to the control groups, we found that α-amanitin-treated embryos exhibited significantly lower L1/B1 segregation indexes and less clear patterns of Hi-C plaids (Fig. 3g, h). At 20 h, α-amanitin-treated embryos showed a low median level of L1/B1 segregation indexes and a Hi-C pattern with extensive diagonal signals that are similar to those of early 2-cell embryos, while the control group had proceeded into the late 2-cell stage (Fig. 3g, h). At 45 h, the control group had proceeded into the 8-cell and morula stages, whereas α-amanitin-treated embryos (45 h) still showed low L1/B1 segregation and a Hi-C plaid pattern similar to that of late 2-cell embryos, despite the segregation index modestly increases compared with that of embryos at 20 h (Fig. 3g, h and Supplementary information, Fig. S7b). These results indicate a delayed and incomplete formation of the higher-order chromatin structure in the absence of zygotic Pol II transcription in mouse.

**Fig. 4:**
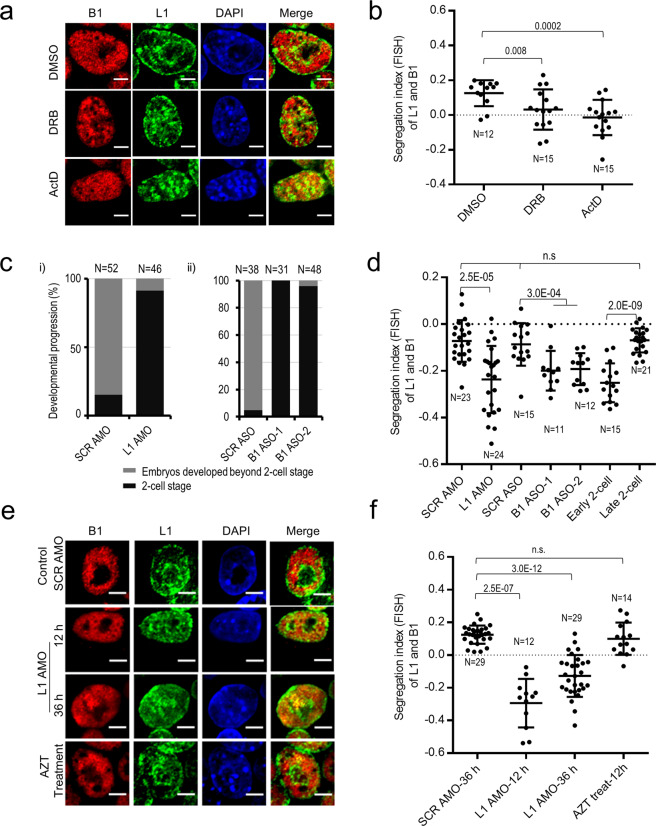
Repeat RNA and transcription promote the spatial segregation of L1 and B1 compartments. **a**, **b** DNA FISH analysis of L1 (green) and B1 (red) repeats in mESCs treated with transcription inhibitors for 3 h. Representative images and the scatterplot of the segregation index of L1 and B1 DNA are shown in **a** and **b**, respectively. DMSO, mock control; DRB (100 μM), a drug inhibits the release and elongation of Pol II; ActD (1 μg/mL), a drug inhibits both Pol I and II. Both drug treatments disrupt the perinucleolar staining of L1 DNA and induce mixing of the L1 and B1 DNA signals. Treatment with ActD elicits a stronger mixing effect than DRB. **c** Developmental analysis of embryos microinjected with scramble (SCR) or L1 AMO (panel (i)) or with SCR or two different B1 ASOs (ASO-1 and ASO-2) (panel (ii)) at the zygote stage. *n*, number of embryos analyzed. **d** Embryos depleted of L1 RNA by AMO or B1 RNA by ASO show poorer L1/B1 segregation, as indicated by significantly lower L1/B1 segregation indexes compared to the scramble AMO/ASO controls and noninjected late 2-cell embryos. Each dot represents an embryo analyzed. Data were collected from three independent experiments. *P* values are calculated with the two-tailed Student’s *t-*test. **e**, **f** DNA FISH analysis of L1 (green) and B1 (red) repeats in mESCs transfected with scramble AMO for 36 h, or L1 AMO for 12 and 36 h, or treated with the drug AZT for 12 h. Representative images and the scatterplot of the segregation index of L1 and B1 DNA are shown in **e** and **f**, respectively. All scale bars, 5 μm. *n*, number of nuclei analyzed. Data are presented as means ± SD (> 3 independent experiments, except two biological replicates for AZT treatment), and *P* values are calculated with two-tailed Student’s *t-*test.

In accordance with delayed chromatin folding in embryos, treatments of mESCs with the drug 5,6-dichloro-1-β-d-ribofuranosylbenzimidizole (DRB) which inhibits Pol II transcription elongation, led to a partial loss of L1 perinucleolar localization and a gain of mixed nuclear L1 and B1 signals (Fig. 4a, b and Supplementary information, Fig. S7c). Inhibition of both Pol I and II by a high concentration of actinomycin D (ActD) had a more severe effect compared to DRB treatment (Fig. 4a, b). Thus, in both ESCs and early embryos, inhibition of Pol II transcription appears to partially, but not completely, block L1/B1 segregation. These results imply that L1/B1 compartmentalization is likely to be autonomously initiated and subsequently facilitated by transcription.

### Repeat transcripts promote L1 and B1 segregation in embryonic cells

In an effort to link repeat function with chromatin structure, we sought to explore the role of repeat RNA that is transcribed from L1 and B1 sequences. Both L1 and B1 repeats are activated and highly expressed in two-cell embryos (Supplementary information, Fig. S7d).^71,84–86^ We have reported previously that depletion of L1 RNA by an antisense morpholino (AMO) in mouse embryos led to arrest at the 2-cell stage; and in mESCs, its depletion led to reduced proliferation and global de-repression of hundreds of L1-associated genes; however, it did not alter the expression of OCT4 and NANOG, two known master regulators of the pluripotency program, nor induced ESC differentiation.^61,87^ Using the same L1 AMO sequence, we depleted L1 RNA by 17.4% on average shown by RNA FISH (*n* = 16 embryos; Supplementary information, Fig. S8a). This modest depletion is consistent with the general consensus that AMO acts through steric blockage of its target RNA rather than inducing RNA degradation. In concordance with the previous report by Percharde et al.,^84^ more than 91.3% of embryos (42 out of 46) were arrested at the 2-cell stage in contrast to only 15.4% of embryos treated with scramble AMO that were arrested (Fig. 4c), indicating effective inhibition of L1 RNA.

We also sought to perturb B1 expression by microinjecting B1 antisense oligonucleotides (ASO) into mouse zygotes. Two B1 ASOs significantly downregulated B1 RNA levels by 36% shown by RNA FISH (*n* = 17 embryos; Supplementary information, Fig. S8b). Strikingly, embryos depleted of B1 RNA were able to pass the first embryonic division, but failed to divide further and became arrested at the 2-cell stage (*n* = 79 embryos; Fig. 4c), indicating an essential requirement for B1 RNA in embryonic development. We collected these embryos for DNA FISH analysis when the control group injected with scramble AMO or ASO had grown to the late 2-cell stage. Compared to the control embryos, both L1- and B1-depleted embryos exhibited significantly lower L1/B1 FISH segregation indexes (Fig. 4d and Supplementary information, Fig. S8c, d), indicating delayed segregation of L1 and B1 compartments.

In order to dissect the effects independent of embryonic progression, we then tried to deplete B1 and L1 transcripts in mESCs. B1/Alu repeats have been broadly implicated in diverse processes, including transcription, RNA processing, and nuclear export.^88–91^ Treatment of mESCs with B1 ASO led to severe cell death within hours of transfection (data not shown), which precluded direct assessment of B1 RNA in chromatin organization. It has been suggested that nuclear organization is critically dependent on interactions within heterochromatin,^34,92^ where L1, the most abundant one of all repeat subclasses, is predominantly enriched. In subsequent analysis, we in-depth characterized the effects of depleting L1 RNA on chromatin organization.

Treatments of mESCs with L1 AMO led to a depletion of L1 RNA by 28% (*n* = 30 cells) (Supplementary information, Fig. S8e, f). At both 12 and 36 h post transfection of L1 AMO, nucleoplasmic signals of L1 DNA were obviously increased and perinucleolar L1 signals became fuzzy or absent (Fig. 4e). B1 signals became more uniformly dispersed in the nucleoplasm, in contrast to punctate staining of B1 in the control mESCs. Emergence of the overlapping L1 and B1 FISH signals is indicative of decreased homotypic clustering and segregation of L1 and B1 DNA. In comparison, treatment with the drug azidothymidine (AZT), which blocks L1 retrotransposition activity,^93^ failed to affect the nuclear localization of L1 and B1 as well as L1 RNA levels (Fig. 4e and Supplementary information, Fig. S8g), illustrating an effect independent of L1 retrotransposition activity.

Image quantification of a large number of L1-depleted cells (*n* = 41 cells randomly picked at 12 and 36 h) showed significantly lower FISH segregation indexes (Fig. 4f), compared to mESCs treated with scramble AMO or AZT (*n* = 43 cells). This indicates that the most majorities of L1 AMO-treated cells exhibit decreased L1/B1 segregation. In contrast, increases of 2-cell-like cells and G2/M-arrested cells occur in small populations, from 2% to 9% and from 13% to 31%, respectively.^87^ In addition, cells in M phase show a drastically different staining pattern of L1 and B1 from cells in S phase (Fig. 3a). These observations argue against a secondary effect due to changes in mESC state upon depletion of L1 RNA.

### Hi-C reveals a key role of L1 RNA in maintaining the 3D chromatin structure

**Fig. 5:**
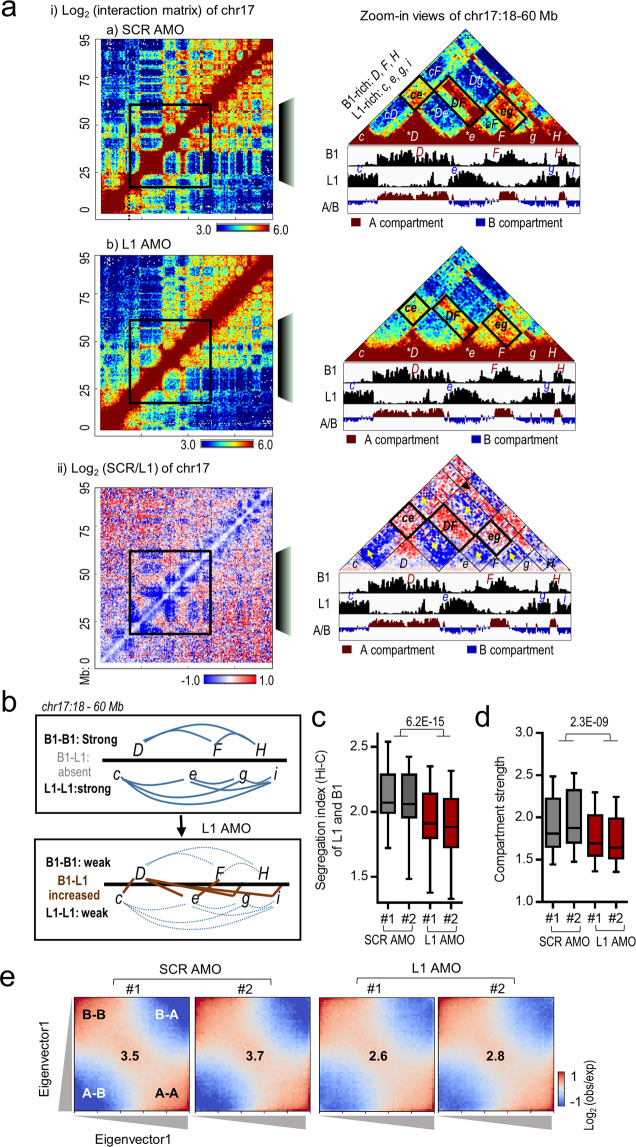
L1 RNA is required for the formation and maintenance of higher-order chromatin structure. **a** Hi-C analysis of mESCs treated with scramble control (SCR) or L1 AMOs for 36 h. Panel (i) shows Hi-C heatmaps of contact frequencies for SCR and L1 AMO, and panel (ii) shows the comparison of contact frequencies between SCR and L1 AMOs [log_2_(SCR/L1)]. The whole chromosome 17 (chr17) is shown on the left and the boxed sub-region (18–60 Mb) of chr17 is shown enlarged on the right (at 500-kb resolution). To better orient the visualization and comparison of these three sub-region heatmaps, representative homotypic interactions (*ce*, *DF*, and *eg*) are labeled with black boxes in the right panels. Relevant B1- and L1-rich compartments are labeled as in Fig. 1. **b** Schematic representation of compartmental interactions before and after depletion of L1 RNA based on Hi-C data shown in **a**. The control mESCs show strong homotypic interactions (indicated by blue solid lines), whereas L1-depleted cells show weakened homotypic interactions (dotted blue lines) and abnormal increases of heterotypic interactions (indicated by brown solid lines). **c** Boxplots of Hi-C-based segregation indexes. L1 AMO led to decreased ratios of homotypic interaction versus heterotypic interaction between L1-rich and B1-rich regions compared to SCR AMO. The *P* value was calculated by the two-tailed Student’s *t*-test. **d**, **e** Analysis of compartment strength showing decreased compartmentalization in mESCs treated with L1 AMOs for 36 h compared to SCR AMO. Boxplots of compartment strength (**d**) and saddle plots of Hi-C data binned at 100 kb resolution (**e**) are shown. The *P* value was calculated by the two-tailed Student’s *t*-test. Saddle plots were calculated using the Eigenvector1. The numbers at the center of the heatmaps indicate compartment strength calculated as the ratio of (A–A + B–B)/(A–B + B–A) using the mean values from the corners as indicated.

To reveal molecular defects in detail, we performed Hi-C analysis at 36 h after transfecting L1 AMO into mESCs. Direct visualization of Hi-C interaction maps revealed obvious differences in the plaid pattern of L1-depleted and control mESCs across mouse chromosomes (Fig. 5a and Supplementary information, Fig. S9a). In L1-depleted mESCs, as illustrated by chr17, Hi-C contact signals were abnormally increased along the diagonal line, whereas the plaid signals outside of the diagonal regions became fuzzy or even lost (Fig. 5a, panel (i)). Accordingly, a comparison of Hi-C contact frequencies of the control versus L1-depleted cells showed decreased ratios (in blue) across the diagonal and increased ratios (in red) in the periphery (Fig. 5a, panel (ii)). Examination of all 20 mouse chromosomes revealed similar changes (Supplementary information, Fig. S9a), indicating enhanced local chromosomal contacts but decreased long-range interactions across the genome in L1-depleted mESCs.

A zoomed-in view of a 42-Mb region of chr17 in L1-depleted mESCs further shows decreased homotypic chromatin contacts between L1-rich or B1-rich compartments, but aberrantly increased heterotypic contacts (Fig. 5a, b). For example, B1–B1 interactions (represented by *DF*, *DH* and *FH)* or L1–L1 interactions (represented by *ce*, *cg*, *ci, eg, ei* and *gi)* were downregulated in L1-depleted cells, whereas aberrant B1–L1 contacts (represented by *cD*, *De*, *Dg, Di, eF, Fg*, and *Hi*) were increased. At the genome-wide level, L1-depleted cells show significantly lower Hi-C segregation indexes of L1 and B1 compared to control mESCs (Fig. 5c).

To evaluate potential changes in the higher-order chromatin structure, we quantified and plotted compartment strength based on the ratio of homotypic (A–A and B–B) to heterotypic (A–B or B–A) compartmental interactions.^34,94^ Saddle plots of compartmental interactions show obvious decreases in homotypic B–B and A–A interactions and increases in aberrant A–B interactions upon L1 AMO (Fig. 5d, e and Supplementary information, Fig. S9b). Compared to scramble AMO samples, L1-depleted cells exhibit significantly reduced compartment strength (2.6–2.8 versus 3.5–3.7, *P* < 0.01). Thus, consistent with DNA FISH, Hi-C analysis further revealed that depletion of L1 RNA causes abnormal increases in heterotypic contacts and genome-wide decreases in homotypic repeat contacts, L1/B1 segregation, and A/B compartmentalization. We noted that depletion of L1 did not alter TAD boundaries (Supplementary information, Fig. S9c). The finding that L1 RNA regulates compartmental organization but not TADs is in line with the notion that the formation of compartments and TADs may involve distinct mechanisms.^29^

### L1 RNA regulates spatial contacts of L1/B1-rich sequences

**Fig. 6:**
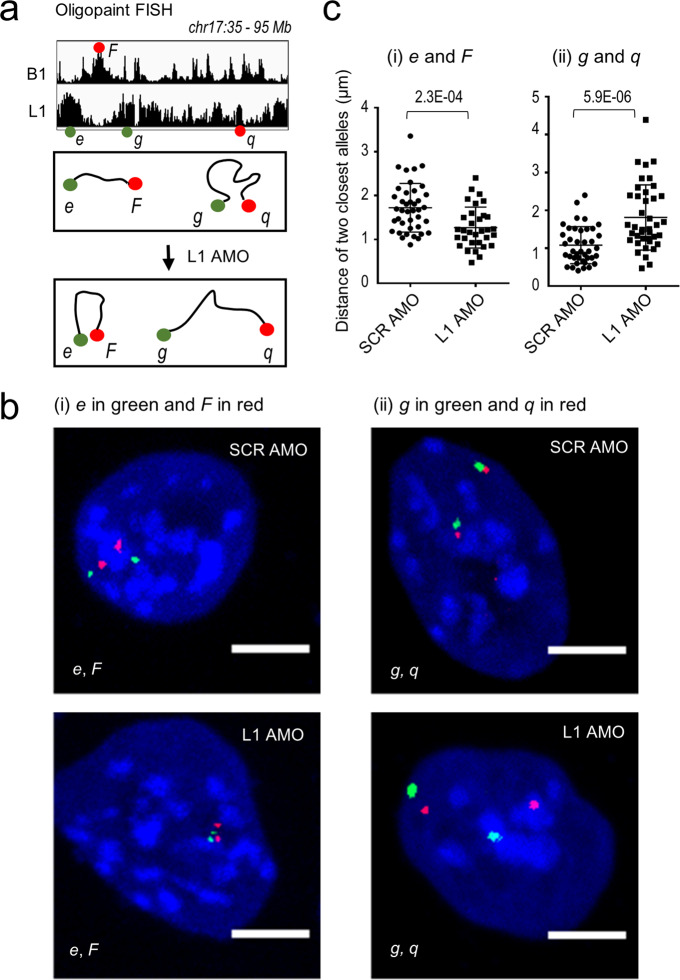
Depletion of L1 RNA alters nuclear localizations of L1/B1-rich sequences. Oligopaint DNA FISH analysis of four representative loci before and after depletion of L1 RNA for 36 h in mESCs. **a** Scheme of relative chromosomal localizations and spatial distances of the four compartments labeled by Oligopaint DNA FISH probes. B1-rich domain: *F*. L1-rich domains*: e, g*, and *q*. **b**, **c** Representative images and quantification of the spatial distances between two labeled compartments are shown in **b** and **c**, respectively. Each dot in **c** represents a nucleus. In cells treated with L1 AMO, the median distance between the two B1 and L1 compartments *e* and *F* is significantly decreased compared to the control, while the median distance between the two L1 compartments *g* and *q* is significantly increased. Data are presented as means ± SD. *n*, number of nuclei analyzed. *P* values are calculated by the two-tailed Student’s *t*-test. Scale bars, 5 μm.

Having shown a global role of L1 RNA in the regulation of chromatin organization, next we performed Oligopaint dual-color DNA FISH to ask whether depletion of L1 might affect the nuclear localization of specific chromosomal segments. We first chose two heterotypic repeat regions on chr17, *e* (L1-rich) and *F* (B1-rich), which are ~100-kb in length covered by 500 oligo probes (Fig. 6a and Supplementary information, Fig. S5b). The *e* and *F* sites are juxtaposed to each other with a linear genomic distance of 4.39 Mb. In the nuclear space, they are positioned far away from each other with a median distance of 1.72 ± 0.55 µm in control mESCs, whereas depletion of L1 RNA significantly shortened the spatial distance between them to 1.27 ± 0.47 µm (Fig. 6b, c, left).

Next, we chose two large L1-rich regions, *g* and *q*, each of which is ~1 Mb in length covered by 4500 probes (Fig. 6a and Supplementary information, Fig. S5b). The *g* and *q* regions are separated by multiple compartmental domains with a linear distance of 26.4 Mb in sequence; however, they reside in close spatial proximity (1.08 ± 0.45 µm) in the nuclei of control cells. L1 depletion significantly increased the nuclear distance between *g* and *q* to 1.81 ± 0.82 µm (Fig. 6b, c, right). These opposing changes observed between homotypic L1–L1 and heterotypic L1–B1 repeat contacts provide visual evidence for L1 RNA in the regulation of nuclear positioning of specific chromosomal segments. We noted that L1 AMO led to moderate but significant increases in the volume of two large L1-rich segments (*g* and *q*), implying heterochromatin decompaction (Supplementary information, Fig. S10a). In addition, L1 AMO did not alter the genome-wide binding of H3K9me3 (Supplementary information, Fig. S11), arguing against an indirect consequence due to loss of heterochromatic histone marks.

Taken together, the combined analyses of the whole nucleus and individual loci by sequencing and imaging approaches convincingly demonstrate that L1 RNA critically promotes spatial interactions of homotypic repeats and compartmental segregation of L1- and B1-rich chromosomal sequences. By comparison, depletion of CTCF or RAD21, the core component of cohesion, failed to affect homotypic contacts and nuclear segregation of L1 and B1 repeats (Supplementary information, Fig. S10b–i), which is consistent with the Hi-C results reported previously.^22,23,95^

### L1 repeats promote phase separation of HP1α

**Fig. 7:**
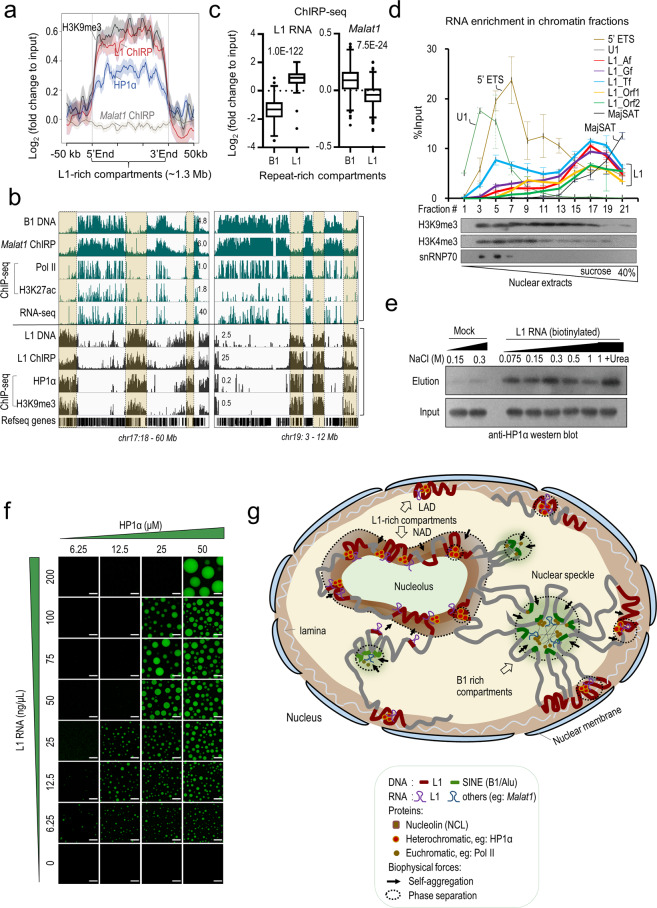
L1 repeats promote phase separation of HP1α. **a** Metagene analysis showing ChIRP DNA-seq signals for L1 and *Malat1* RNA, and ChIP-seq signals for HP1α and H3K9me3 in L1-rich compartments. ChIRP-seq and ChIP-seq reads densities were normalized to the input DNA. The shadow around each line represents standard error. **b** Genome browser view of sequencing tracks in two regions in mouse chr17: 18–60 Mb (left) and chr19: 3–12 Mb (right). The first five rows show the genomic density of B1 repeats, B1-related sequencing data (ChIRP-seq signals of *Malat1*, ChIP-seq signals of Pol II, and H3K27ac), and RNA-seq in mESCs. The lower tracks show the genomic density of L1 repeats (highlighted by beige shading) and L1-related sequencing data, including ChIRP-seq signals of L1 RNA, ChIP-seq signals of HP1α and H3K9me3. Refseq gene annotations are also included. **c** Boxplot showing the ChIRP-seq signal of L1 and *Malat1* RNA in B1 and L1-rich compartments. *Y*-axis showing the fold change of raw ChIRP-seq signal to input DNA. *P* values were calculated with two-tailed Student’s *t*-test and are shown in each plot. **d** RT-qPCR analysis of relative enrichments of various transcripts in chromatin fractions of mESCs separated by sucrose gradient centrifugation. Enrichment of RNA in each fraction was normalized to the input nuclear extracts (top). Data are shown as means ± SD (*n* = two biological replicates). Western blot analysis confirms the effectiveness of chromatin fractionation (bottom). **e** Biotinylated L1 RNA pulls down recombinant HP1α protein. Reactions without addition of L1 RNA were served as mock control. **f** Representative images of droplet formation at different concentrations of HP1α protein and L1 RNA. Concentrations of HP1α and RNA are indicated at the top and left of the images, respectively. Scale bars, 10 μm. Data are representative of three independent experiments. **g** The model. First, the intrinsic self-assembly property of L1 and B1/Alu repeats provides numerous nucleation points to seed the formation of nuclear subdomains. Repetitive DNA sequences also serve as anchor sites for transcription machinery, regulatory proteins and RNAs. Second, the embedded structural information in DNA repeats may be translated by their RNA transcripts, together with interacting DNA- and/or RNA-binding proteins. Molecular crowding generated by interactions among DNA sequences, RNAs, and proteins in subnuclear domains seeded by individual clusters of L1 or B1/Alu, may drive the aggregation of compartments containing the same repeat type through a phase-separation mechanism, which consequently folds the genome. Third, the nuclear segregation of L1-rich compartments and B1/Alu-rich compartments may be further reinforced by attaching their DNA sequences to subnuclear structures such as nuclear speckles and the nucleolus, respectively, which serve as scaffolds to stabilize the nuclear architecture.

To have a glimpse of the mechanism by which L1 regulates chromatin organization, we investigated the interplay of L1 repeat DNA and RNA with HP1α, a known H3K9me3 reader in heterochromatin organization. We have shown that HP1α binds strongly to L1-rich heterochromatin, but is depleted in B1-rich regions (Figs. 1a and 7a–c and Supplementary information, Fig. S12a). Previously, we performed chromatin isolation by L1 RNA purification followed by sequencing (ChIRP-seq).^61^ L1 RNA is significantly enriched in L1 DNA-associated compartments with high levels of H3K9me3 and HP1α signals, but is depleted in B1-associated compartments (Fig. 7a–c). We enriched heterochromatins by sucrose-gradient centrifugation of UV-crosslinked chromatin fragments and performed transcript analysis. Consistently, L1 transcripts are preferentially enriched in heterochromatic fractions that are depleted of H3K4me3 and snRNP70 (Fig. 7d). In addition, L1 RNA signals show no overlap with B1 RNA in the nucleus as shown by RNA FISH (Supplementary information, Fig. S12b).

HP1α has been reported to bind RNA with a preference towards nuclear RNA and major forward transcripts produced from satellite repeats.^96–99^ RNA immunoprecipitation in mESCs showed that HP1α binds to L1 transcripts (Supplementary information, Fig. S12c, d). To test their direct interactions, we purified human HP1α proteins and various RNA fragments in roughly 1-kb length transcribed in vitro. The full-length L1 sequence (6544 bp) was arbitrarily truncated into 8 overlapping 1-kb fragments (F1 to F8) in order to be efficiently produced by in vitro transcription (Supplementary information, Fig. S13a). For comparison, we also generated two synthetic DNA and RNA sequences in 1-kb length, comprising 8 tandem copies of either B1 element or scrambled B1 (designated as 8× B1 or 8× SCR, respectively). L1 mix as well as two synthetic fragments in DNA or RNA efficiently pulled down recombinant HP1α (Supplementary information, Fig. S13b). In addition, L1 RNA–HP1α interactions were robustly detected in highly stringent conditions with up to 1 M of salt and urea (Fig. 7e). These results indicate that HP1α exhibits strong binding activities towards RNA and DNA in vitro.

Recent studies have reported that HP1α forms phase-separated droplets in the presence of DNA or nucleosomes in vitro, and heterochromatin formation may entail a phase-separation mechanism.^37,38,100–102^ Indeed, the L1 DNA mix as well as two synthetic DNA controls (8× B1 and 8× SCR) promote the phase separation of HP1α, which fails to phase separate on its own (Supplementary information, Fig. S13c, d). Consistent with its strong RNA-binding activity, HP1α also phase separates with the L1 RNA mix in a concentration-dependent manner to form spherical droplets with liquid-like properties, such as fusion of droplets and rapid recovery after photo-bleaching (Fig. 7f and Supplementary information, Fig. S13d–f). Intriguingly, L1 RNA fragments from F3 to F6 with low GC contents (< 40%) covering the inter-ORF and central conserved ORF2 sequence (3.2-kb) tend to have higher activities in promoting HP1α droplet formation compared to F1 and F8 fragments with GC contents of 45%–56% (Supplementary information, Fig. S13g, h). In addition, careful examination of HP1α ChIP-seq in mESCs showed that HP1α preferentially binds to the central region of L1 repeat DNA (Supplementary information, Fig. S13i), which hints some degree of specificity in L1–HP1α interplay. However, there was no obvious difference detected between L1 mix and 8× B1 (60% in GC) RNA/DNA in HP1α phase separation (Supplementary information, Fig. S13j). Although we cannot conclude a sequence specificity of HP1α, clearly, HP1α shows strong RNA- and DNA-binding activities and phase-separates in the presence of RNA and DNA in vitro. Given the abundance and co-residence of L1 repeat DNA and RNA with HP1α within the nucleus, it is tempting to speculate that their co-localization may provide a means of specificity for L1 in promoting HP1α phase separation during heterochromatin formation.

## Discussion

Although tremendous efforts have been dedicated to studies of structural chromatin proteins and cataloging chromatin maps, the role of DNA sequences in 3D genome organization has been largely ignored. Interestingly, the overall higher-order chromatin structure has been reported to be stable across different cell types and conserved in related species, despite occasional compartment switches in a small portion of the genome in a given cell (Supplementary information, Text S1). This remarkable conservation of chromatin compartments suggests a fundamental principle which all cells stick to while coping with shifting signals in different cell fates. Compared to transcription and epigenetic modifications, the primary DNA sequence has an unparalleled advantage to directly control and govern the stability of 3D genome folding due to its static nature during development. Then, the question comes what DNA sequences serve such a task as the blueprint of 3D genome folding. By employing in silico polymer simulations to interpret microscopy and Hi-C data, Mirny and colleagues have suggested that compartmental segregation may occur through a microphase-separation mechanism of block copolymers.^29,34^ Together with the Solovei group, they further proposed that interactions between heterochromatic regions, rather than euchromatic contacts and lamina-heterochromatin interactions, are crucial for compartmentalization of the genome in both inverted and conventional nuclei.^29,34^ However, what was unknown in their model is the molecular determinants, particularly the genetic information of block copolymers that are responsible for chromatin compartmentalization.

In this study, we reveal a remarkable correlation between repeat distribution and compartmental organization of the higher-order chromatin structure. First, using complementary genomics and imaging approaches, we demonstrate that the self-clustering of L1 and B1/Alu repeats forms grossly exclusive nuclear domains that are highly correlated with and predict the known A/B compartments, and that nuclear segregation of L1-rich and B1/Alu-rich sequences is conserved across mouse and human cells, and can be dynamically established during cell division and early embryogenesis. Second, we show that depletion of L1 RNA by AMOs drastically alters repeat segregation and compartmentalization on a global scale and at individual loci by Hi-C, DNA FISH and Oligopaint FISH. Collectively, the overall positive correlation and the essentiality of L1 RNA in compartmental organization suggest a functional role for L1 repeats in driving genome folding. These results disfavor the notion that L1 or B1/Alu repeats are merely markers of large chromosomal segments with a different activity, although we cannot firmly exclude this possibility. Our model is also consistent with the growing evidence showing active roles of retrotransposons in re-wiring the genome and regulatory programs.^53–61^ As often a challenge for genome organization studies, we note that the current support for going beyond correlative evidence to really show a causative role of L1 is still limited.

L1 RNA tends to co-localize with L1 DNA sequences in regions enriched for the binding of HP1α and H3K9me3. Intriguingly, L1 RNAs can also be detected outside the nuclear and nucleolar periphery (Supplementary information, Fig. S12b). L1 RNA has a short half-life of 40 min.^61^ Torres-Padilla and colleagues reported previously that exogenous L1 RNA fails to rescue the chromatin defects upon abnormal silencing of L1 in mouse zygotes.^71^ Although these observations disfavor a *trans*-acting mechanism, it is possible that L1 transcripts might be mobilized to distal L1 DNA sequences. As 20%–40% of L1 repeats are located in annotated euchromatic compartments (Supplementary information, Fig. S1e), these L1 transcripts may be more readily visualized by microscopy. For the majority of L1 enriched in transcriptionally silenced heterochromatic environments, their expression might be temporally regulated in a more transient way, thus creating difficulty for direct visualization and detection. Studies of X chromosome inactivation revealed different roles for silenced and actively transcribed L1s in regulating heterochromatin formation induced by *Xist*.^103^ Silent L1 repeats participate in the assembly of a heterochromatic compartment, whereas transient transcription of certain young L1s facilitates local propagation of the silencing into regions that would be otherwise prone to escape. Recently, we have reported that depletion of L1 RNA leads to relocation of L1-rich DNA from inactive domains to the nuclear interior and genome-wide de-repression of L1-associated genes.^61^ Together, these results indicate a role of L1 RNA in mediating its own DNA’s function. However, the questions arise of where and when L1 RNA is produced, how it is regulated, and whether L1 transcription also plays a role during heterochromatin formation and compartmentalization.

Recombinant HP1α binds and phase separates with all tested DNA and RNA fragments in vitro, yet L1 RNAs in the central conserved region (inter-ORF and ORF2) show high activities compared to L1 sequences in the 5’ and 3’ ends. In mESCs, HP1α tends to bind the central region of L1 repeat DNA. These observations imply some degree of weak sequence-specificity for HP1α in recognizing its targets. This notion is congruent with several reports that HP1α preferentially binds nuclear RNA and rRNA rather than tRNA and randomly chosen RNA,^98^ and binds the forward strand but not the reverse of major repeat RNA.^99^ In addition, extensive co-localization of L1 and HP1α suggests a location-derived specificity in cells, while other DNA- and/or RNA-binding proteins may also endow the specificity of endogenous HP1α to L1 repeats. On the other hand, in the presence of substantial concentrations of macromolecules in a crowded milieu of living cells, even non-specific interactions may contribute considerably to total free energy that drives the phase separation of heterochromatic domains.^104^ Together, these results implicate L1 RNA and DNA in the phase separation of heterochromatin formation. Future work should dissect the specific domains or sequence features of L1 that mediate its interactions with HP1α and address their interplays in vivo.

As a chromatin-bound noncoding RNA, L1 RNA may facilitate heterochromatin formation through a number of ways, such as promoting HP1α phase separation by providing a means of multivalency and recruiting RNA-binding proteins to increase local molecular mass, or stabilizing DNA-binding activities of heterochromatin-associated proteins (for example, HP1α binds both L1 RNA and DNA, in a manner similar to YY1^105^), or acting as a scaffold to anchor L1-rich chromosomal segments to the nucleolar and nuclear peripheries. Cases for RNA in organizing subnuclear domains have been reported. For example, *Xist* RNA binds the lamin B receptor in the inner nuclear membrane to anchor the inactive X chromosome.^106^ Transcription of satellite repeats precedes chromocenter formation and their transcripts help to recruit SUV39H to centromeric DNA sequences.^107,108^ In-depth investigation of the expression, function and mechanism of L1 RNA could be a subject of future studies.

Based on our results together with previous reports, we propose a hypothetical model in which repetitive elements organize the macroscopic structure of the genome at three hierarchical levels (Fig. 7g). First, B1/Alu and L1 repeats serve as the genetic basis for A and B compartments, respectively. The abundance and scattering of these repeat elements in the genome provide numerous nucleation points or ‘structural codes’ to seed the formation of nuclear subdomains. Homotypic clustering of L1-rich or B1/Alu-rich regions initiates genome folding. Second, the structural information embedded in linear genomic DNA repeats is in part transacted by their transcripts, particularly L1 RNA as demonstrated in this study, into spatially ordered chromatin in the nucleus, likely through a phase-separation mechanism. Phase separation of individual subdomains based on differences in activity and protein composition may also lead to their segregation in the nuclear space and the eventual formation of distinct L1-rich heterochromatin and B1/Alu-rich euchromatin domains. However, the contribution of B1/Alu transcripts to 3D genome organization remains to be tested. Third, chromatin compartmentalization may be further reinforced and stabilized through the attachment of repeat DNA sequences to subnuclear structures such as the nucleolus and nuclear speckles.^109^ Evidence to support this notion includes our observation that L1 repeats are preferentially localized at the nuclear and nucleolar peripheries, and depletion of L1 RNA disrupts the localization of L1 DNA at these sites.

L1 or B1/Alu DNA tends to associate with distinct sets of histone marks and transcription and chromatin regulators.^61^ For example, heterochromatin proteins such as HP1α, KAP1, and SETDB1 are specifically enriched on L1 elements, whereas general transcription factors such as GTF3C2 and CEBPB and RNA Pol II subunits show enriched binding on B1/Alu.^61^ We envision that repeat RNA may provide additional layers of regulatory specificity and multivalency to generate molecular crowding. Specific interactions among similar RNAs and proteins at homologous L1 or B1/Alu chromosomal segments may not only enhance subdomain formation, but also promote their segregation through phase separation. Selectivity for similar binding partners has been reported by the Tjian group.^110^ There does not appear to be cross-talk between different transcription factors; instead, they interact among themselves to form concentrated hubs on synthetic lacO DNA arrays in cells.^110^ In addition, different biophysical properties of phase-separated L1 or B1 subdomains, for example chromatin compactness, may further promote their segregation.

We have shown that inhibition of Pol II in both embryos and mESCs led to delayed and incomplete formation of L1/B1 segregation and Hi-C plaid patterns without abolishing genome folding. Previously, the Solovei group reported clustering of exogenous sequences with genomic segments of the same repeat class.^44^ When a human artificial chromosome was introduced into mouse ESCs, human L1s spatially interact with mouse L1-rich regions but avoid the SINE-rich regions, and vice versa for human SINEs.^44^ Based on these findings, we posit that L1/B1 repeats may represent autonomously functional units of the genome, and homotypic repeat clustering initiates compartmentalization, which is subsequently facilitated by transcription. An autonomous model for DNA sequence-dependent nuclear organization has been well demonstrated by the self-assembly of tandem repeat sequences such as ribosomal DNA (rDNA) and satellite repeats, which promotes high-order assemblages of the nucleolus and pericentromeric domains, respectively.^111,112^

Although different in nucleotide sequences and length, both primate-specific Alu and rodent-specific B1 elements belong to the same class of SINE repeats, which originate from a common ancient ancestor, 7SL RNA, prior to the primate-rodent split about 80 million years ago,^113^ arguing against convergent evolution in driving retrotransposons in genome organization. Comparative genomics analysis across mammalian genomes has revealed that large-scale conserved patterns of retrotransposon accumulation follow similar evolutionary trajectories through conservation of synteny, gene regulation and nuclear organization, in spite of dissimilar retrotransposons.^114^ In addition, it has been reported that the landscape of endogenous L1 elements differs significantly from that of new L1 retrotransposon insertions, which broadly target all regions of the human genome, being insensitive to chromatin state.^115,116^ This suggests that purifying selection, rather than biased insertions, reshapes the genomic distributions of L1 and Alu/B1 post their integration.^61,115,117,118^ We speculate that the genetic marking of compartments with distinct activity is so important that during evolution it has imposed selective pressures on the most abundant subfamilies of LINE and SINE repeats for them to accumulate in specific compartments. In comparison, we find ERV retrotransposons to be randomly distributed, which is consistent with a previous report by the Ren group that no genome-wide enrichment of ERVs was found at TAD boundary.^13^ Recently, the Ren group reported that transcriptionally active HERV-H repeats, a subclass of ERVs, demarcate TADs in human hESCs.^119,120^ In fact, among > 1000 HERV-H repeats in human, < 50 show detectable expression in hESCs and only ~20 have a TAD boundary structure as indicated by directionality index. It is likely that a very small proportion of ERVs act at TAD and loop boundaries for more specific local chromatin regulation in a few genomic loci.

In summary, our study provides important initial evidence to unravel the fundamental principle of 3D genome organization. As discovered by Anfinsen in the late 1950s, the amino acid sequence of a protein determines its structure and function.^121,122^ Analogously, we propose that the primary DNA sequences, particularly L1 and B1/Alu elements, dictate how the genome folds and functions. We envision that genome folding occurs autonomously, through a process that is driven by homotypic clustering of regions containing L1 or B1/Alu repeat sequences, which could be further facilitated by transcription processes, transcripts produced at these repeat elements, regulatory proteins, and perhaps a combination of these factors that act above and beyond repeat DNA sequences to influence their chromatin states. The widespread yet conserved distribution of homologous repeats in mammalian genomes render them a unique advantage to perform such a task as the blueprint for genome organization and function, compared to histone marks and transcription activities. Structural information embedded in L1 and B1/Alu repeats may be universally recognizable, thus contributing to the high degree of stability and conservation in the compartmental organization that is observed across mouse and human cell types. We want to note that L1 and B1/Alu compartmental domains represent a structural and functional ground state of chromatin organization, on which subsequent regulatory features, such as dynamic enhancer–promoter interactions, are overlaid. Nevertheless, the same principle of homotypic clustering, phase separation and spatial segregation of chromosomal segments may be reiterated at different genomic scales, consequently folding the genome. Lastly, our study calls for more work towards a complete understanding how genome folding occurs, particularly, on revealing the causality and mechanisms how these repetitive sequences act.

## Materials and methods

### Genomic analysis of repeat sequences

The reference catalog of repetitive elements was built from RepeatMasker annotations.^123^ We used the 10-kb bin in all repeat analyses unless otherwise indicated. For de novo compartment calling, the mouse genome was first segmented into 100-kb bins, and the densities of L1 and B1 repeats were normalized to their genome background (19% for L1 and 3% for B1), and log_2_ of the ratio of normalized B1 to L1 densities [log_2_(B1/L1)] was calculated. The adjacent regions with size >500 kb were kept (85% left) and assigned as B1-rich (540) or L1-rich (648) compartments with a median size of 1.1 Mb to 1.3 Mb (Supplementary information, Table S1).

### mESC and embryonic experiments

For cell-cycle synchronization, mESC (J1) cells^124^ were treated with 1.25 mM Thymidine for 14 h and then 50 ng/mL Nocodazole for 7 h. G1 and S phase cells were collected at 1.5 h and 7 h, respectively, after Nocodazole release. mESCs were treated with DRB (100 μM) and ActD (1 μg/mL) for 3 h to inhibit transcription. For heterochromatin fractionation, sucrose gradient centrifugation of mESC nuclear extracts was performed as previously reported with modifications.^125,126^ For embryonic microinjection, ASO (5 μM) and AMO (1 mM) was injected into PN3 zygotes on a Leica DMI3000B microscope equipped with a Leica micromanipulator.

### Imaging analysis

DNA FISH,^4^ immuno-FISH,^109^ and RNA FISH^127^ in mESCs and embryos were performed as previously described with modifications. FISH Probes targeting consensus sequence of L1 and B1 (Fig. 2a) were used for both mESCs and embryos (Supplementary information, Table S2). For Oligopaint FISH analysis of non-repetitive sequences in A/B compartments, each of four regions in ~100-kb length, including B1-rich regions *F, H* and *R*, and L1-rich region *e*, is targeted by 500 DNA probes; and each of two large L1-rich regions *g* and *q* (~1 Mb) is targeted by 4500 DNA probes. Probe sets are shown in Supplementary information, Table S3. Image acquisition and quantification were conducted with UltraVIEW VoX spinning disc microscope (PerkinElmer) and Imaris version 8.4.1.

### RNA depletion by AMO or ASO

To deplete L1 RNA in mouse embryos and mESCs, we used the same morpholino antisense oligonucleotide (AMO or ASO) as Percharde et al. previously used.^87^ To deplete B1 RNA, two antisense oligonucleotide (ASO) sequences targeting the B1 consensus sequence were synthesized by IDT (Integrated DNA technologies). Sequences are listed in Supplementary information, Table S4.

### Hi-C analysis

Small-scale in situ Hi-C (sisHi-C) was performed as previously described,^81^ following two independent experiments of L1 or scramble (SCR) AMO transfection. The summary statistics for Hi-C quality control is shown in Supplementary information, Table S5. Paired-end raw reads of Hi-C library data were processed with HiCPro (version 2.7.7) as described.^128^ A and B compartments^6^ and compartmentalization strength^23,34,94^ were identified as described previously.

### Segregation index

The FISH-based segregation index is defined as the negative value of Pearson’s correlation coefficient of L1 and B1 DNA signals in the nucleus. The Hi-C-based segregation index is defined as the ratio of homotypic versus heterotypic interaction frequencies between L1 and B1/Alu subfamilies.

### In vitro pull-down and phase separation assays

A series of eight 1-kb fragments, designated as F1 to F8, were produced to cover the full-length 6544-kb L1 sequence by PCR (Supplementary information, Fig. 13a). Two artificial fragments comprising eight tandem copies of either B1 element (8× B1, in 1-kb length) or scrambled B1 sequence (8× SCR, in 1-kb length) were used for comparison. Biotin-labeled RNA was obtained by in vitro transcription for the pull-down experiment. We purified the recombinant human HP1α protein as previously described.^38^ In phase separation assays, recombinant HP1α with DNA or RNA fragments for L1, 8× B1, or 8× SCR were incubated at 4 °C overnight.

### Statistical analysis

Statistical analyses were carried out using Excel or R (version 3.4.3).

Please also see Supplementary information, Data S1 for details.

## Supplementary information

Supplementary information, Figure S1

Supplementary information, Figure S2

Supplementary information, Figure S3

Supplementary information, Figure S4

Supplementary information, Figure S5

Supplementary information, Figure S6

Supplementary information, Figure S7

Supplementary information, Figure S8

Supplementary information, Figure S9

Supplementary information, Figure S10

Supplementary information, Figure S11

Supplementary information, Figure S12

Supplementary information, Figure S13

Supplementary information, Table S1

Supplementary information, Table S2

Supplementary information, Table S3

Supplementary information, Table S4

Supplementary information, Table S5

Supplementary information, Text S1

Supplementary information, Data S1

## Data Availability

Sequencing data generated in this study have been deposited into the Gene Expression Omnibus database under accession numbers GSE123806 and GSE125766.
