# Supplementary material for: Homotypic clustering of L1 and B1/Alu repeats compartmentalizes the 3D genome
Source: Cell Res. 2021 Jan 29;31(6):613–30. doi: 10.1038/s41422-020-00466-6 (PMC8169921; doi:10.1038/s41422-020-00466-6)
Supplement: Supplementary file 1 — Supplementary information, Figure S1 [file 41422_2020_466_MOESM1_ESM.pdf]

Fig. S1

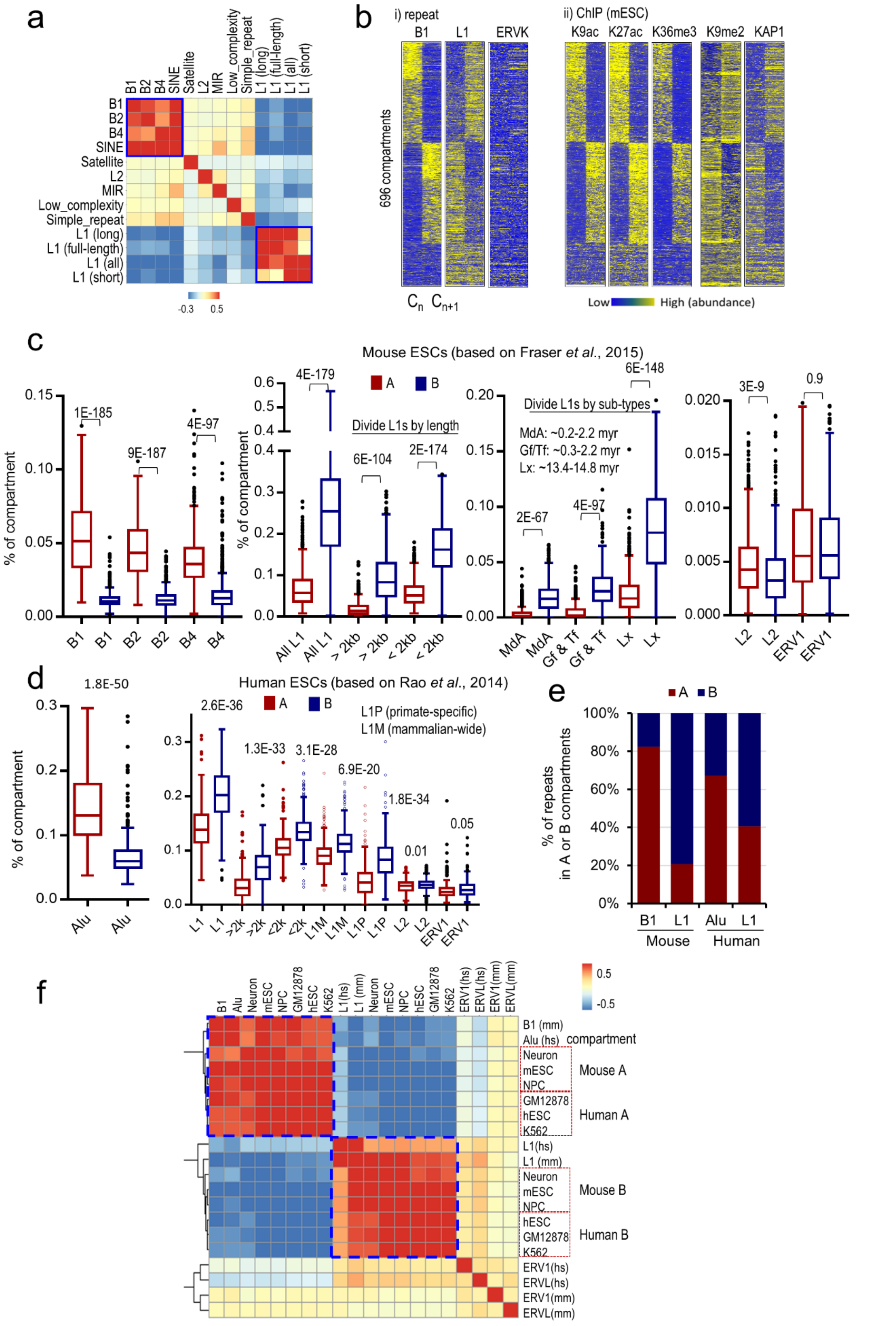
a
B1
B2
B4
SINE
Satellite
L2
MIR
Low_complexity
Simple_repeat
L1 (long)
L1 (full-length)
L1 (all)
L1 (short)
-0.3
0.5
b
i) repeat
B1
L1
ERVK
ii) ChIP (mESC)
K9ac
K27ac
K36me3
K9me2
KAP1
696 compartments
C_n C_{n+1}
Low
High (abundance)
c
Mouse ESCs (based on Fraser et al., 2015)
% of compartment
1E-185
9E-187
4E-97
4E-179
A
B
Divide L1s by length
6E-104
2E-174
All L1
> 2kb
< 2kb
6E-148
Divide L1s by sub-types
MdA: ~0.2-2.2 myr
Gf/Tf: ~0.3-2.2 myr
Lx: ~13.4-14.8 myr
2E-67
4E-97
MdA
Gf & Tf
Lx
3E-9
0.9
L2
ERV1
d
Human ESCs (based on Rao et al., 2014)
1.8E-50
Alu
2.6E-36
1.3E-33
3.1E-28
6.9E-20
1.8E-34
0.01
0.05
L1P (primate-specific)
L1M (mammalian-wide)
L1
>2k
<2k
L1M
L1P
L2
ERV1
e
A
B
% of repeats in A or B compartments
100%
80%
60%
40%
20%
0%
B1
L1
Alu
L1
Mouse
Human
f
0.5
-0.5
B1 (mm)
Alu (hs)
compartment
Neuron
mESC
NPC
Mouse A
GM12878
hESC
K562
Human A
L1(hs)
L1 (mm)
Mouse B
Human B
ERV1(hs)
ERVL(hs)
ERV1(mm)
ERVL(mm)

Fig. S1 Nonrandom distribution of B1 and L1 repeats.

(a) Unsupervised hierarchical clustering of mouse repeats based on genomic position. As L1 elements are frequently truncated at their 5′ ends during replication, we further divided all L1 repeats (all: 905,176 copies) into three subgroups according to their length in the genome: short (< 2 kb, 861,570 copies), long (>2 kb, 43,606 copies), and full-length (>5 kb, 15,580 copies).

(b) Heatmaps of the distribution densities of B1, L1 and ERVK repeats (panel i), and ChIP-seq signals of H3K9ac, H3K27ac, H3K36me3, H3K9me3 and KAP1 (panel ii) across two adjacent compartments (C_n, C_{n+1}). All signals in 696 compartments annotated in mESCs were sorted according to the B1 distributions shown in panel (i).

(c) Boxplots showing mouse repeat content in A and B compartments annotated in mESCs. The y-axis shows the percentage of a repeat subclass in either an A or B compartment. Different repeat sub-classes are shown: B1, B2 and B4 repeats (left), L1 repeats (middle two panels), and ERV1 and L2 repeats (right). As L1 elements are frequently truncated at their 5′ ends during replication, all L1 repeats were further divided into evolutionary old (Lx) or young (MdA, Gf and Tf) and short (< 2 kb) or long (>2 kb) L1s groups according to their genomic length. *p* values are calculated with the two-tailed Student’s *t*-test.

(d) Boxplot analysis of human repeat content in A and B compartments annotated in hESCs. Different repeat subclasses are shown: Alu repeats (left), and L1 and ERV1 repeats (right). We divided L1 repeats into short (< 2 kb), long (>2 kb), primate-specific L1P family and the older mammalian-wide L1M family. *p* values are calculated with the two-tailed Student’s *t*-test.

(e) Percentages of L1 or B1/Alu repeats in A or B compartments in mouse (the left two columns) and in human (the right two columns).

(f) Unsupervised hierarchical clustering of repeat content and the corresponding PC1 values of the Hi-C contact matrix in the syntenic regions between human and mouse. The eigenvalues of the Hi-C contact matrix representing A and B compartments from six cell types (mESCs, neural progenitor cells (NPC), neurons and hESC, K562, GM12878) are highly similar, suggesting that A and B compartments are conserved across different cell types and species. A compartments show a strong positive correlation with Alu/B1 repeats but a negative correlation with L1 repeats. The opposite pattern is observed for B compartments.
