# Supplementary material for: Homotypic clustering of L1 and B1/Alu repeats compartmentalizes the 3D genome
Source: Cell Res. 2021 Jan 29;31(6):613–30. doi: 10.1038/s41422-020-00466-6 (PMC8169921; doi:10.1038/s41422-020-00466-6)
Supplement: Supplementary file 2 — Supplementary information, Figure S2 [file 41422_2020_466_MOESM2_ESM.pdf]

Fig. S2

a

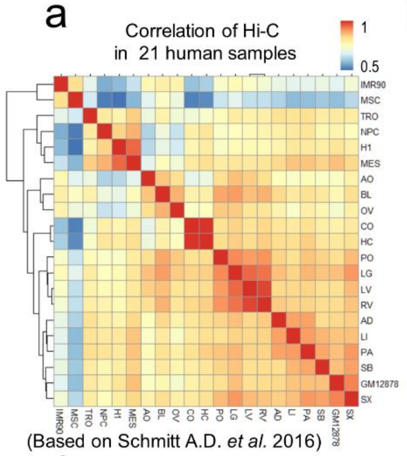

(Based on Schmitt A.D. *et al.* 2016)

b

% of genome with conserved compartments in various tissues

# of tissues	individual %		cumulative %	
	Obs.	Exp.	Obs.	Exp.
= 21	40%	0%	40%	0%
≥ 20	12%	0%	53%	0%
≥19	9%	0%	61%	0%
≥18	7%	1%	69%	1%
≥17	6%	2%	75%	3%
≥16	5%	5%	80%	7%
≥15	5%	9%	84%	16%
≥14	4%	15%	88%	31%
≥13	4%	20%	92%	51%
≥12	3%	22%	96%	73%
≥11	4%	27%	100%	100%

c

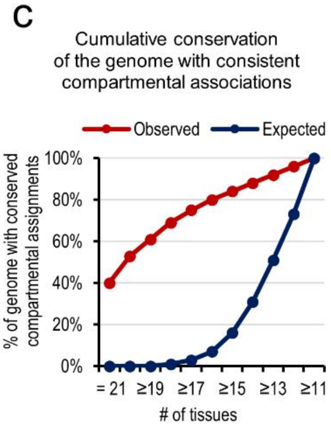

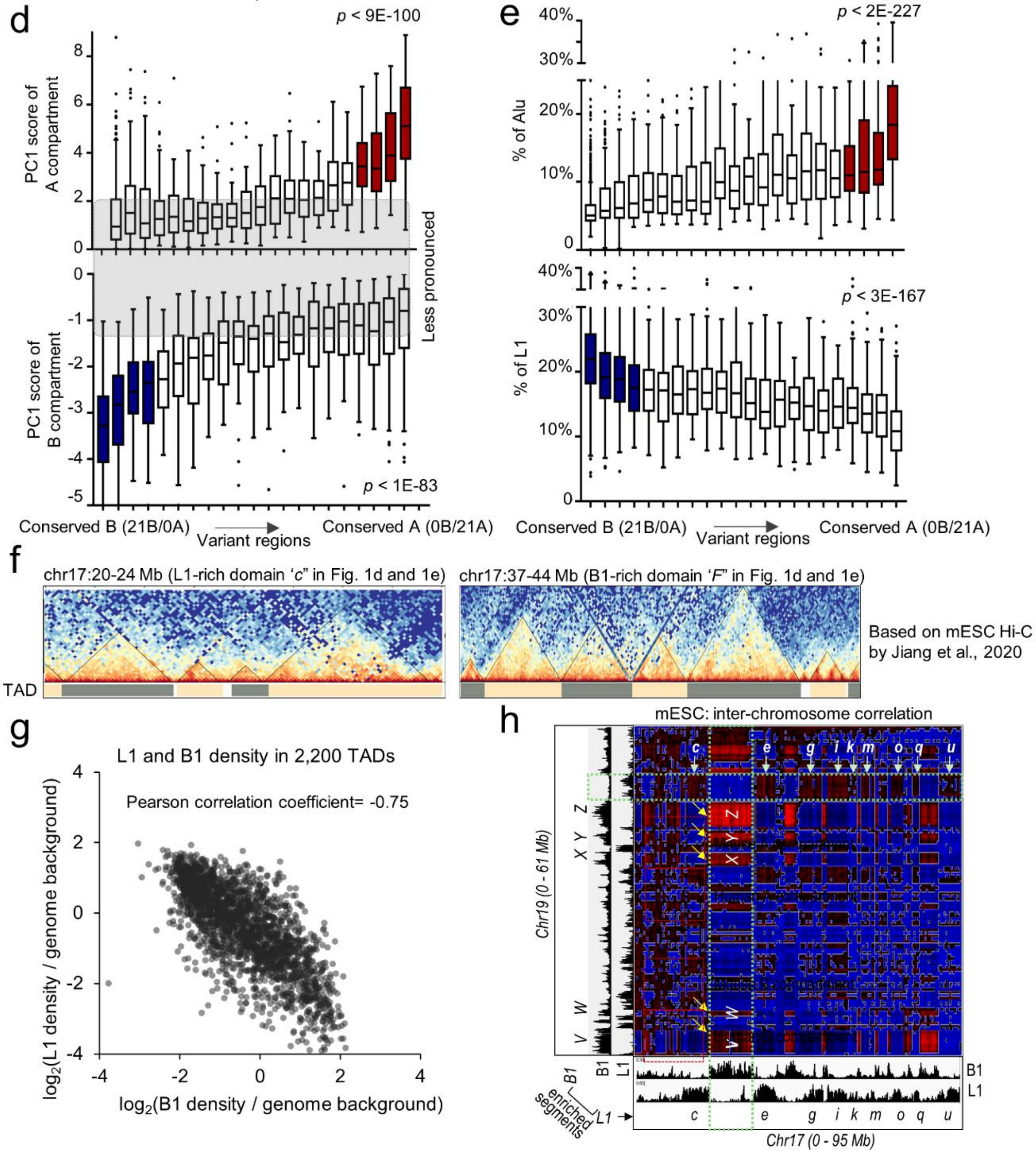

Fig. S2 Conservation of compartmental organization across 21 human cell lines and tissues. (a-e) Analysis of compartmental conservation based on PC1 values of the Hi-C contact matrix in 21 human cell lines and tissues report by Schmitt *et al*[1]. PC1 values of Hi-C contact matrix were obtained from Table S2 of the previous report by Schmitt *et al*[1].

(a) Unsupervised hierarchical clustering of PC1 values of the Hi-C contact matrix in 21 human cell lines and tissues. Genomic positions of A/B compartments are highly similar across cell types, with an average Spearman correlation coefficient ranging 0.47-0.99 and a median value of 0.79.

(b) Percentage of the human genome with conserved compartments in various numbers of tissues.

(c) Cumulative conservation of the genome with consistent compartmental association based on panel (b).

(d) Boxplot showing the PC1 values of genomic regions with different compartmental assignments by Hi-C. The y axis is the PC1 value of A (upper) or B (lower) compartments. The x axis shows genomic regions with various compartmental assignments that are sequentially arranged as 21B/0A, 20B/1A, 19B/2A, …, 2B/19A, 1B/20A, 0B/21A from left to right. The label "21B/0A" or ''0B/21A'' means the genomic regions where all 21 samples have a compartment B or A label, respectively, thus representing the most conserved regions. The label ''20B/1A'' represents the genomic regions where 20 samples exhibit a compartment B label and 1 sample exhibit a compartment A label. The far left (for example, 21B/0A, 20B/1A, 19B/2A, and 18B/3A, highlighted in dark blue) and far right columns (for example, 3B/18A, 2B/19A, 1B/20A, and 0B/21A, highlighted in dark red) represent genomic regions with more conserved B or A compartmental assignment, respectively. The middle columns represent the genomic regions that tend to be more variant across different tissues and show less pronounced PC1 scores (arbitrarily defined based on *p* values; highlighted in grey). *p* values to the most conserved A and B (21B/0A and 0B/21A) are calculated with two-tailed Student's *t*-test.

(e) Boxplots showing the density of Alu (top) and L1 (bottom) in genomic regions with different compartmental assignments by Hi-C. Similar to (d), boxplots in the left and right regions represent genomic regions with conserved B and A compartment assignments, respectively, whereas the genomic regions in the middle tend to be more variant across different tissues. *p* values of conserved A and B are calculated with two-tailed Student's *t*-test.

(f) Heatmaps of B1-rich (chr17:20-24 Mb) and L1-rich (chr17: 37-44 Mb) regions, related to Fig. 1e. Annotated TADs are clearly illustrated and confirmed by the Hi-C contact heatmaps

(g) Scatter plot showing distribution of L1 and B1 repeats in 2,200 TADs identified by Dixon *et al*., 2012[2].

(h) Correlation heatmap showing Pearson correlation coefficients of inter-chromosomal interaction frequencies between chromosomes 17 and 19 at 500-kb resolution. B1-rich regions on chr19 are arbitrarily labeled in uppercase as *V, W, X, Y,* and *Z*. L1-rich regions on chr17 are labeled as shown in panels (E-F) from *c, e … q,* to *u*. The horizontal dotted box and arrows highlight positive correlations of an L1-rich region on chr19 with other L1-rich regions on chr17. The vertical dotted box and arrows show positive correlations of a B1-rich region on chr17 with other B1-rich regions on chr19.

References:

1. Schmitt, A. D. *et al.* A Compendium of Chromatin Contact Maps Reveals Spatially Active Regions in the Human Genome. *Cell Rep* **17**, 2042-2059, doi:10.1016/j.celrep.2016.10.061 (2016).
2. Dixon, J. R. *et al.* Topological domains in mammalian genomes identified by analysis of chromatin interactions. *Nature* **485**, 376-380, doi:10.1038/nature11082 (2012).
