# Supplementary material for: Homotypic clustering of L1 and B1/Alu repeats compartmentalizes the 3D genome
Source: Cell Res. 2021 Jan 29;31(6):613–30. doi: 10.1038/s41422-020-00466-6 (PMC8169921; doi:10.1038/s41422-020-00466-6)
Supplement: Supplementary file 3 — Supplementary information, Figure S3 [file 41422_2020_466_MOESM3_ESM.pdf]

Fig. S3

De novo compartment calling based on L1 and B1 DNA sequences

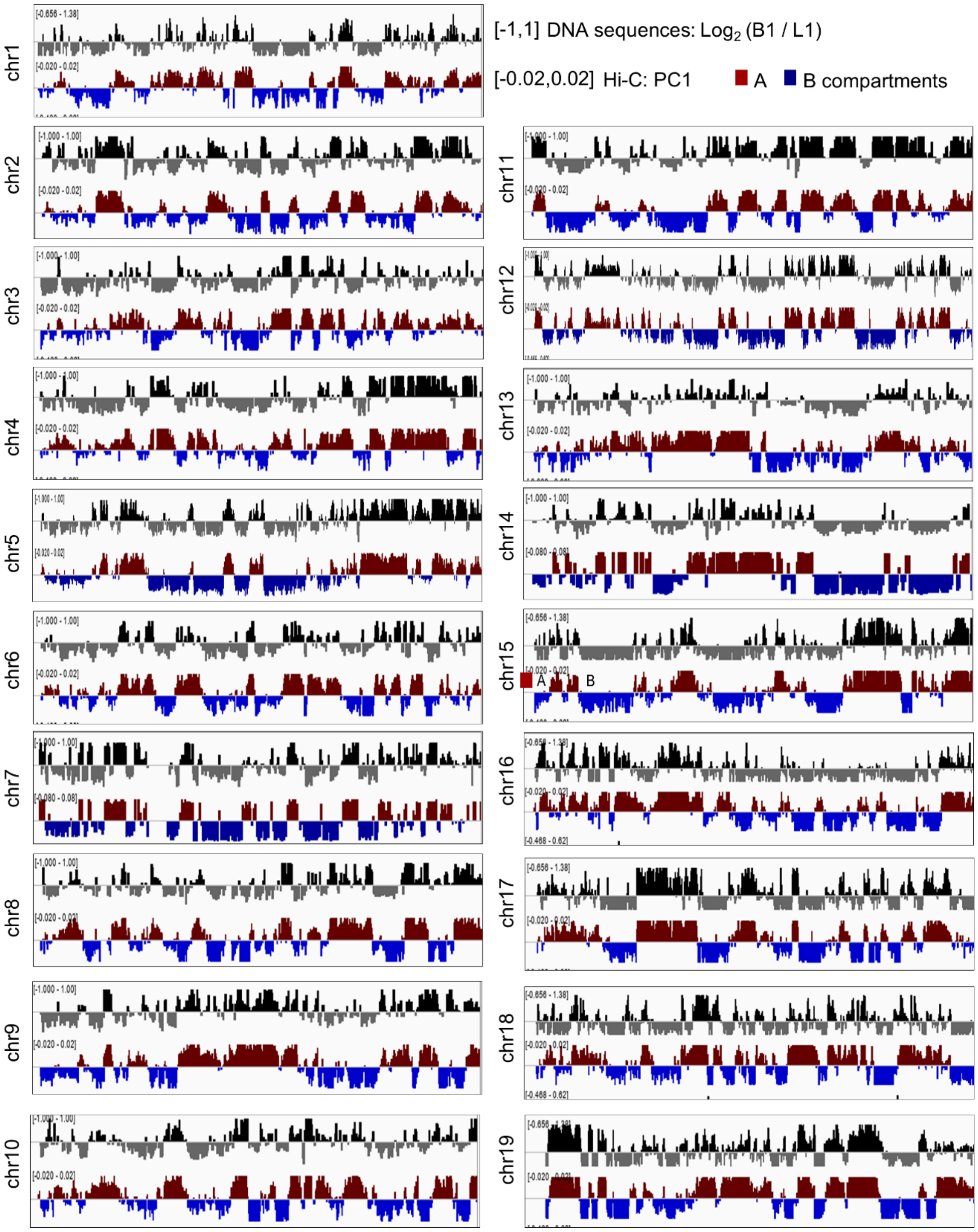

Fig. S3 *De novo* compartment calling based on L1 and B1 DNA sequences.

All mouse chromosomes showing the ratio of B1 to L1 density in $\log_2$ scale [$\log_2$ (B1 / L1)] (upper) and the PC1 score of Hi-C interaction matrix (lower) at 20-kb resolution.
