# Supplementary material for: Homotypic clustering of L1 and B1/Alu repeats compartmentalizes the 3D genome
Source: Cell Res. 2021 Jan 29;31(6):613–30. doi: 10.1038/s41422-020-00466-6 (PMC8169921; doi:10.1038/s41422-020-00466-6)
Supplement: Supplementary file 4 — Supplementary information, Figure S4 [file 41422_2020_466_MOESM4_ESM.pdf]

Fig. S4

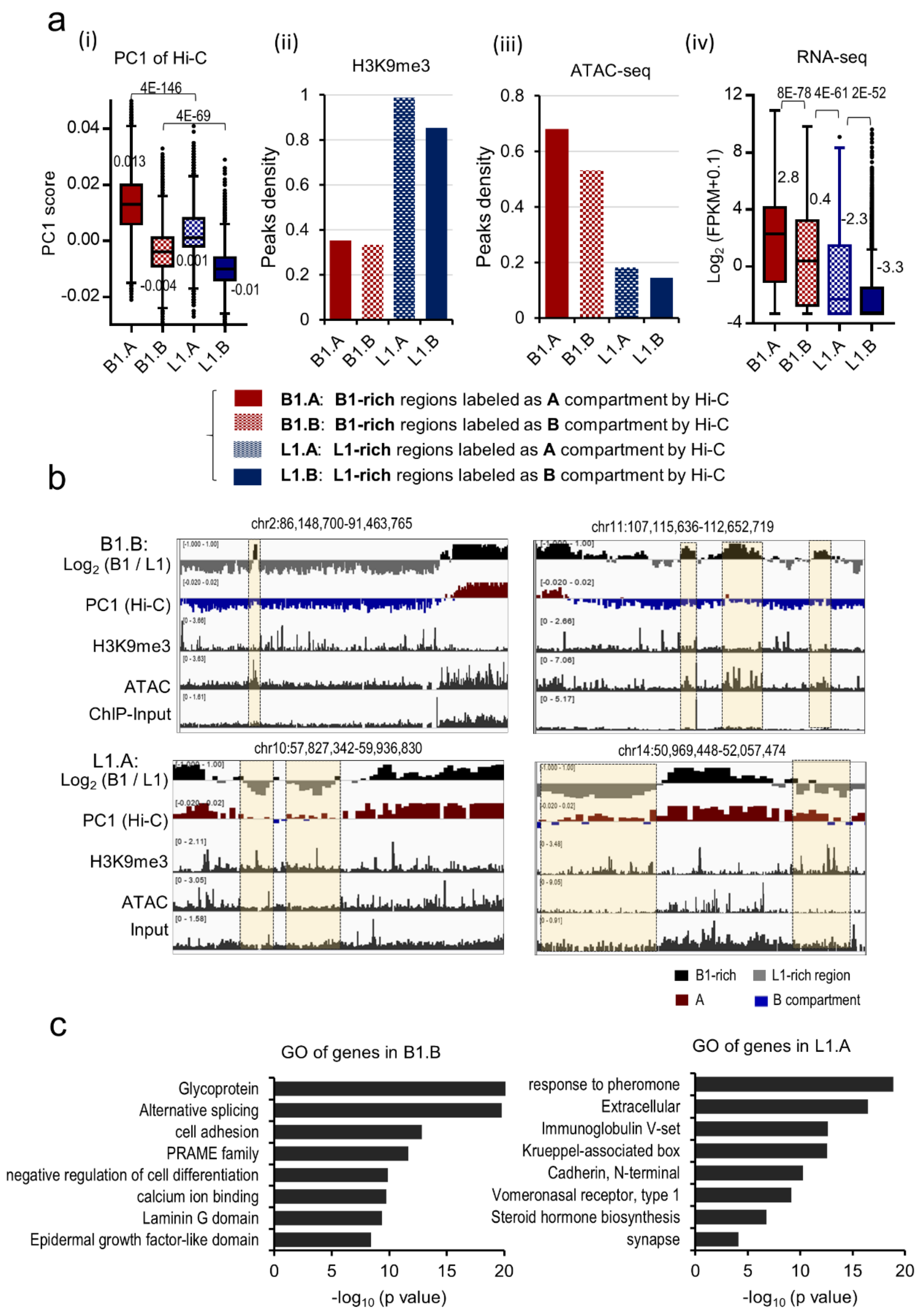

a
(i) PC1 of Hi-C
PC1 score
4E-146
4E-69
0.013
0.001
-0.004
-0.01
B1.A B1.B L1.A L1.B
(ii) H3K9me3
Peaks density
(iii) ATAC-seq
Peaks density
(iv) RNA-seq
Log2 (FPKM+0.1)
8E-78 4E-61 2E-52
2.8
0.4
-2.3
-3.3
B1.A: B1-rich regions labeled as A compartment by Hi-C
B1.B: B1-rich regions labeled as B compartment by Hi-C
L1.A: L1-rich regions labeled as A compartment by Hi-C
L1.B: L1-rich regions labeled as B compartment by Hi-C
b
B1.B:
Log2 (B1 / L1)
PC1 (Hi-C)
H3K9me3
ATAC
ChIP-Input
chr2:86,148,700-91,463,765
chr11:107,115,636-112,652,719
L1.A:
Log2 (B1 / L1)
PC1 (Hi-C)
H3K9me3
ATAC
Input
chr10:57,827,342-59,936,830
chr14:50,969,448-52,057,474
B1-rich
L1-rich region
A
B compartment
c
GO of genes in B1.B
Glycoprotein
Alternative splicing
cell adhesion
PRAME family
negative regulation of cell differentiation
calcium ion binding
Laminin G domain
Epidermal growth factor-like domain
-log10 (p value)
GO of genes in L1.A
response to pheromone
Extracellular
Immunoglobulin V-set
Krueppel-associated box
Cadherin, N-terminal
Vomeronasal receptor, type 1
Steroid hormone biosynthesis
synapse
-log10 (p value)

Fig. S4 Sequence and chromatin features of genomic regions with consistent or inconsistent compartmental labeling.

(a) Chromatin features in four genomic regions with consistent or inconsistent compartmental labeling between *de novo* prediction and Hi-C. Genomic regions with consistent labeling include “B1.A” and “L1.B” which represent B1-rich or L1-rich regions that fall into Hi-C annotated A or B compartments, respectively. The inconsistent labeled regions include “B1.B” and “L1.A” which represent B1-rich or L1-rich regions that fall into Hi-C annotated B or A compartments, respectively . Boxplots in panel (i) show PC1 values of Hi-C interaction matrix in each group. Panels (ii) and (iii) show the peak densities of heterochromatic H3K9me3 mark and ATAC-seq. Boxplots in panel (iv) show the gene expression level in each group. *p* values are calculated with two-tailed Student’s *t*-test.

(b) Representative genomic regions with inconsistent compartmental labeling between *de novo* prediction and Hi-C. Inconsistently labeled B1.B (upper) and L1.A (lower) regions are highlighted in yellow. They tend to have low PC1 values. B1.B regions tend to show high ATAC-seq signals. L1.A regions tend to show low ATAC-seq signals but high H3K9me3 signals.

(c) Gene ontology analysis of genes in genomic regions with inconsistent compartmental labeling. Left: B1.B; Right: L1.A.
