# Supplementary material for: Homotypic clustering of L1 and B1/Alu repeats compartmentalizes the 3D genome
Source: Cell Res. 2021 Jan 29;31(6):613–30. doi: 10.1038/s41422-020-00466-6 (PMC8169921; doi:10.1038/s41422-020-00466-6)
Supplement: Supplementary file 5 — Supplementary information, Figure S5 [file 41422_2020_466_MOESM5_ESM.pdf]

Fig. S5

a

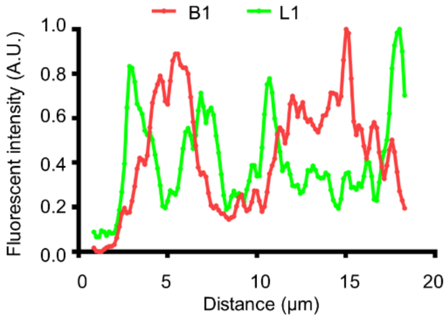

B1
L1
Fluorescent intensity (A.U.)
1.0
0.8
0.6
0.4
0.2
0.0
0
5
10
15
20
Distance (μm)

b

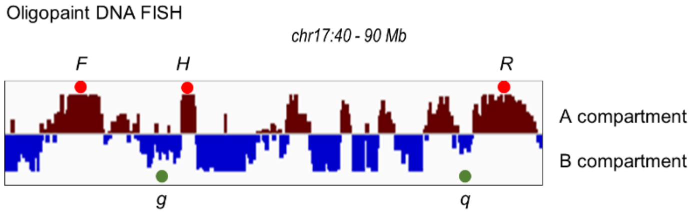

Oligopaint DNA FISH
chr17:40 - 90 Mb
F
H
R
A compartment
B compartment
g
q

c

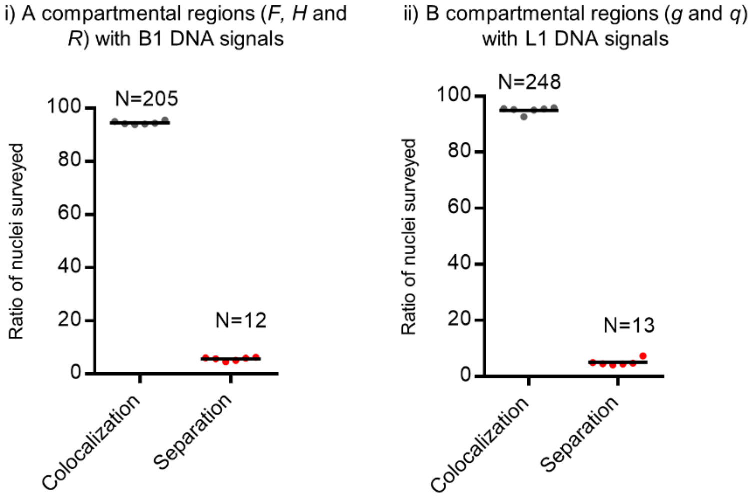

i) A compartmental regions (F, H and R) with B1 DNA signals
N=205
N=12
Ratio of nuclei surveyed
100
80
60
40
20
0
Colocalization
Separation
ii) B compartmental regions (g and q) with L1 DNA signals
N=248
N=13
Ratio of nuclei surveyed
100
80
60
40
20
0
Colocalization
Separation

Fig. S5 Oligopaint and DNA FISH analysis of A and B compartments.

(a) Normalized fluorescence intensity profiles along the white line in the merged B1/L1 image shown in panel Fig. 2b. The L1 channel shows four intensity peaks that surround the two intensity peaks in the B1 channel. These two peaks in the B1 channel correspond to valleys in the L1 channel.

(b) Scheme of chromosomal localizations and spatial distances of the five compartments labeled by Oligopaint DNA FISH probes. A-compartment-associated regions: *F, H and R*. B compartment-associated regions*: g and q.*

(c) Statistic summary of FISH signals to show nuclear colocalization of A- or B-compartment-associated genomic sequences with B1 or L1 repetitive DNA, respectively, as shown in Fig. 2d. i) A compartmental regions (*F, H* and *R*) with B1 DNA signals. ii) B compartmental regions (*g* and *q*) with L1 DNA signals. Each dot represents a field of view in microscope. N represents the total number of cells counted.
