# Supplementary material for: Homotypic clustering of L1 and B1/Alu repeats compartmentalizes the 3D genome
Source: Cell Res. 2021 Jan 29;31(6):613–30. doi: 10.1038/s41422-020-00466-6 (PMC8169921; doi:10.1038/s41422-020-00466-6)
Supplement: Supplementary file 6 — Supplementary information, Figure S6 [file 41422_2020_466_MOESM6_ESM.pdf]

Fig. S6

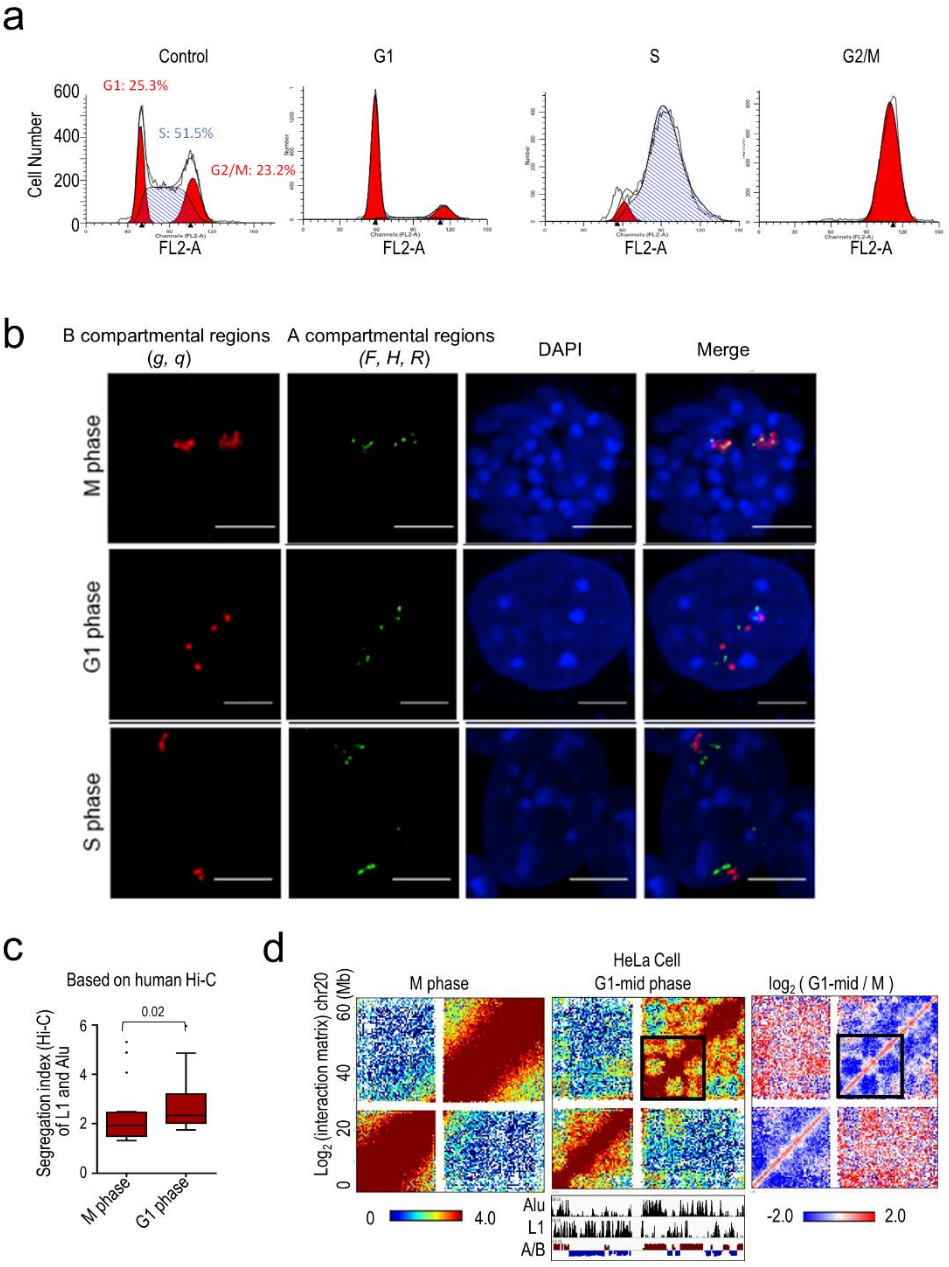
a
Control
G1
S
G2/M
G1: 25.3%
S: 51.5%
G2/M: 23.2%
Cell Number
600
400
200
0
FL2-A
b
B compartmental regions (g, q)
A compartmental regions (F, H, R)
DAPI
Merge
M phase
G1 phase
S phase
c
Based on human Hi-C
0.02
Segregation index (Hi-C) of L1 and Alu
6
4
2
0
M phase
G1 phase
d
HeLa Cell
M phase
G1-mid phase
log2 (G1-mid / M)
Log2 (interaction matrix) chr20 (Mb)
60
40
20
0
4.0
Alu
L1
A/B
-2.0
2.0

Fig. S6 Dynamic establishment of higher-order chromatin organization during the cell cycle.

(a) FACS analysis of cell-cycle synchronized mESCs.

(b) Oligopaint DNA FISH analysis of representative compartmental regions during cell cycle progression from the M phase to the G1 and S phase. A-compartment associated regions: *F, H, R* (green color); B-compartment associated regions: g, *q* (red color), as illustrated in Fig. S5b.

(c) Boxplot analysis of the ratio of homotypic versus heterotypic contacts for Alu-rich and L1-rich regions based on Hi-C data from cell cycle-synchronous HeLa cells. The *y*-axis shows the Hi-C segregation index [(Alu.Alu and L1.L1) / Alu.L1] (female cell line, X chromosomes excluded). *p* values are calculated with two-tailed Student's *t*-tests.

(d) Heatmap of normalized interaction frequencies at 500-kb resolution on chromosome 20 in Hela cell at M (left) and G1-mid (middle) phase. Comparison of contact frequencies between M and G1-mid phase [$\log_2$(G1-mid/M)] for the whole chromosome 20 are shown on the right. Genomic densities of Alu and L1 repeats are shown at the bottom.
