# Supplementary material for: Homotypic clustering of L1 and B1/Alu repeats compartmentalizes the 3D genome
Source: Cell Res. 2021 Jan 29;31(6):613–30. doi: 10.1038/s41422-020-00466-6 (PMC8169921; doi:10.1038/s41422-020-00466-6)
Supplement: Supplementary file 7 — Supplementary information, Figure S7 [file 41422_2020_466_MOESM7_ESM.pdf]

Fig. S7

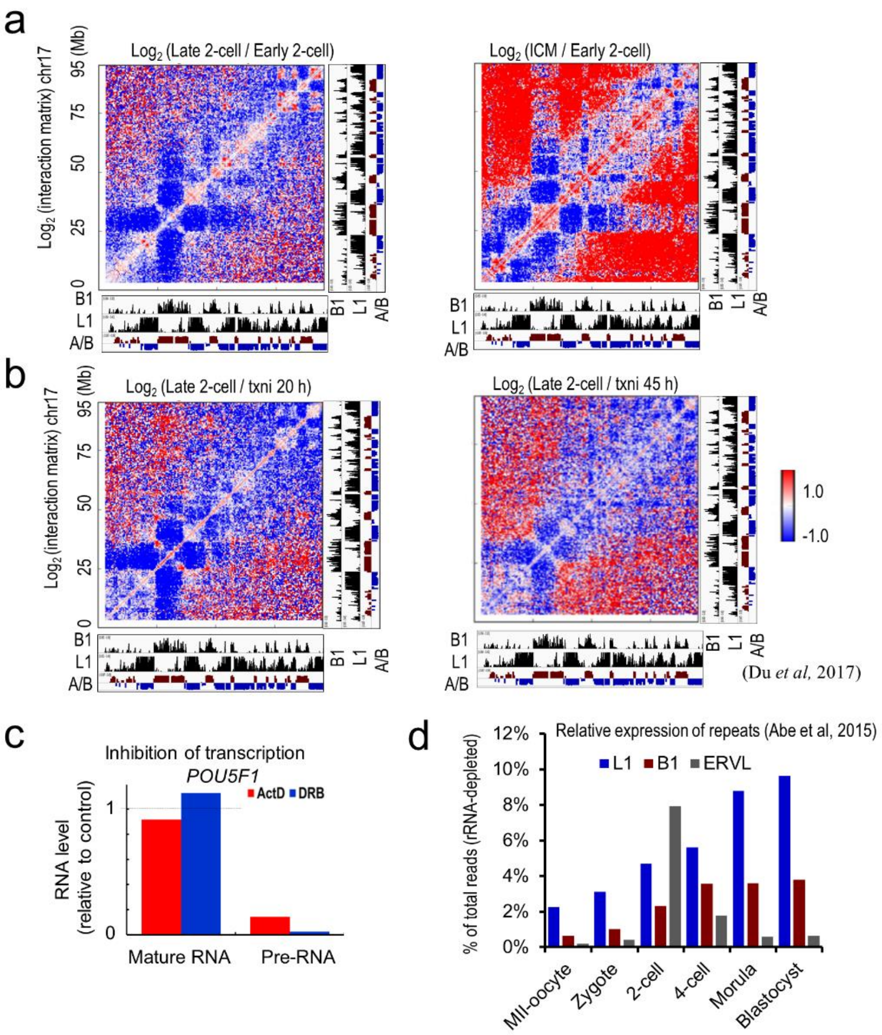
a
Log2 (Late 2-cell / Early 2-cell)
Log2 (ICM / Early 2-cell)
Log2 (interaction matrix) chr17
95 (Mb)
75
50
25
0
B1
L1
A/B
b
Log2 (Late 2-cell / txni 20 h)
Log2 (Late 2-cell / txni 45 h)
1.0
-1.0
(Du et al, 2017)
c
Inhibition of transcription
POU5F1
ActD
DRB
RNA level (relative to control)
1
0
Mature RNA
Pre-RNA
d
Relative expression of repeats (Abe et al, 2015)
L1
B1
ERVL
% of total reads (rRNA-depleted)
12%
10%
8%
6%
4%
2%
0%
MII-oocyte
Zygote
2-cell
4-cell
Morula
Blastocyst

Fig. S7 Dynamic establishment of higher-order chromatin organization during the early embryonic development.

(a) Heatmap showing comparisons of contact frequencies of late 2-cell embryos (left) and ICM (right) to early 2-cell embryos.

(b) Heatmap showing comparisons of contact frequencies of late 2-cell embryos to embryos treated with the transcription inhibitor α-amanitin for 20 hours (left) and 45 hours (right).

(c) Relative expression of *POU5F1* mature RNA and pre-mRNA in mESCs after treatment with the transcription inhibitors DRB and ActD for 3 hours. Both drug treatments greatly reduced the level of *POU5F1* pre-mRNA, while the level of mature *POU5F1* mRNA was not obviously changed, demonstrating successful inhibition of transcription.

(d) Relative expression of various repeats during embryogenesis. The y-axis represents the percentage of total reads in sequencing analysis of rRNA-depleted RNA collected from various stages of embryos based on Abe *et al.* (2015)[3].

Reference

3. Abe, K. *et al.* The first murine zygotic transcription is promiscuous and uncoupled from splicing and 3' processing. *EMBO J* **34**, 1523-1537, doi:10.15252/embj.201490648 (2015).
