# Supplementary material for: Homotypic clustering of L1 and B1/Alu repeats compartmentalizes the 3D genome
Source: Cell Res. 2021 Jan 29;31(6):613–30. doi: 10.1038/s41422-020-00466-6 (PMC8169921; doi:10.1038/s41422-020-00466-6)
Supplement: Supplementary file 8 — Supplementary information, Figure S8 [file 41422_2020_466_MOESM8_ESM.pdf]

Fig. S8

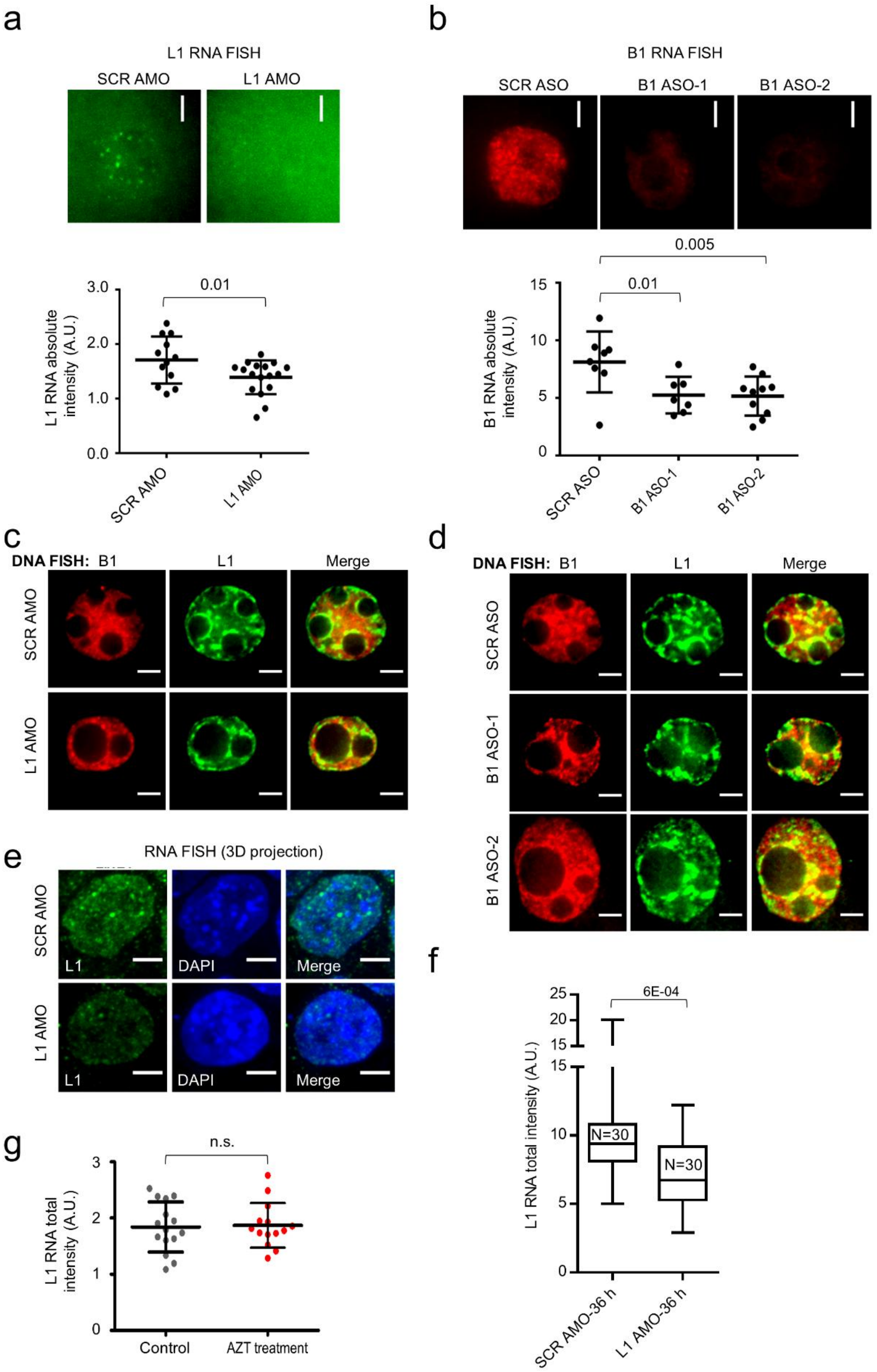
a
L1 RNA FISH
SCR AMO
L1 AMO
0.01
L1 RNA absolute intensity (A.U.)
3.0
2.0
1.0
0.0
SCR AMO
L1 AMO
b
B1 RNA FISH
SCR ASO
B1 ASO-1
B1 ASO-2
0.005
0.01
B1 RNA absolute intensity (A.U.)
15
10
5
0
SCR ASO
B1 ASO-1
B1 ASO-2
c
DNA FISH: B1
L1
Merge
SCR AMO
L1 AMO
d
DNA FISH: B1
L1
Merge
SCR ASO
B1 ASO-1
B1 ASO-2
e
RNA FISH (3D projection)
SCR AMO
L1 AMO
L1
DAPI
Merge
f
6E-04
L1 RNA total intensity (A.U.)
25
20
15
15
10
5
0
N=30
N=30
SCR AMO-36 h
L1 AMO-36 h
g
n.s.
L1 RNA total intensity (A.U.)
3
2
1
0
Control
AZT treatment

Fig. S8 L1 and B1 repeat transcripts promote L1/B1 segregation in embryonic cells.

(a) RNA FISH analysis of L1 (green) RNA in late 2-cell-stage embryos microinjected with scramble (SCR) or L1 AMO at the zygote stage.

(b) RNA FISH analysis of B1 (red) RNA in late 2-cell-stage embryos microinjected with scramble ASO, B1 ASO-1 or B1 ASO-2 at the zygote stage. Panels (a-b) show representative images (upper) and scatterplots of the absolute intensity of L1 or B1 RNA (lower).

(c) DNA FISH analysis of B1 (red) and L1 (green) DNA in late 2-cell-stage embryos microinjected with scramble or L1 AMO at the zygote stage.

(d) DNA FISH analysis of B1 (red) and L1 (green) DNA in late 2-cell-stage embryos microinjected with scramble, B1 ASO-1 or B1 ASO-2 at the zygote stage.

(e-f) RNA FISH analysis of L1 (green) RNA in mESCs transfected by scramble AMO or L1 AMO for 36 hours. Representative images (e) and the boxplot of the L1 RNA absolute intensity (f) are presented.

(g) RNA FISH analysis of L1 RNA in mESCs treated with the drug AZT for 12 hours, or mock control. The scatterplot of L1 RNA total intensity is shown. N, number of nuclei analyzed.

In panels showing scatterplots and boxplots, data are presented as mean ± SD, and *p* values are calculated with the two-tailed Student's t-test; n.s. represents $p>0.05$. In panels showing RNA FISH, images show maximal intensity projections of z confocal sections; representative images were acquired using the same imaging condition and intensity threshold. Scale bars, 5 μm.
