# Supplementary material for: Homotypic clustering of L1 and B1/Alu repeats compartmentalizes the 3D genome
Source: Cell Res. 2021 Jan 29;31(6):613–30. doi: 10.1038/s41422-020-00466-6 (PMC8169921; doi:10.1038/s41422-020-00466-6)
Supplement: Supplementary file 9 — Supplementary information, Figure S9 [file 41422_2020_466_MOESM9_ESM.pdf]

Fig. S9

a

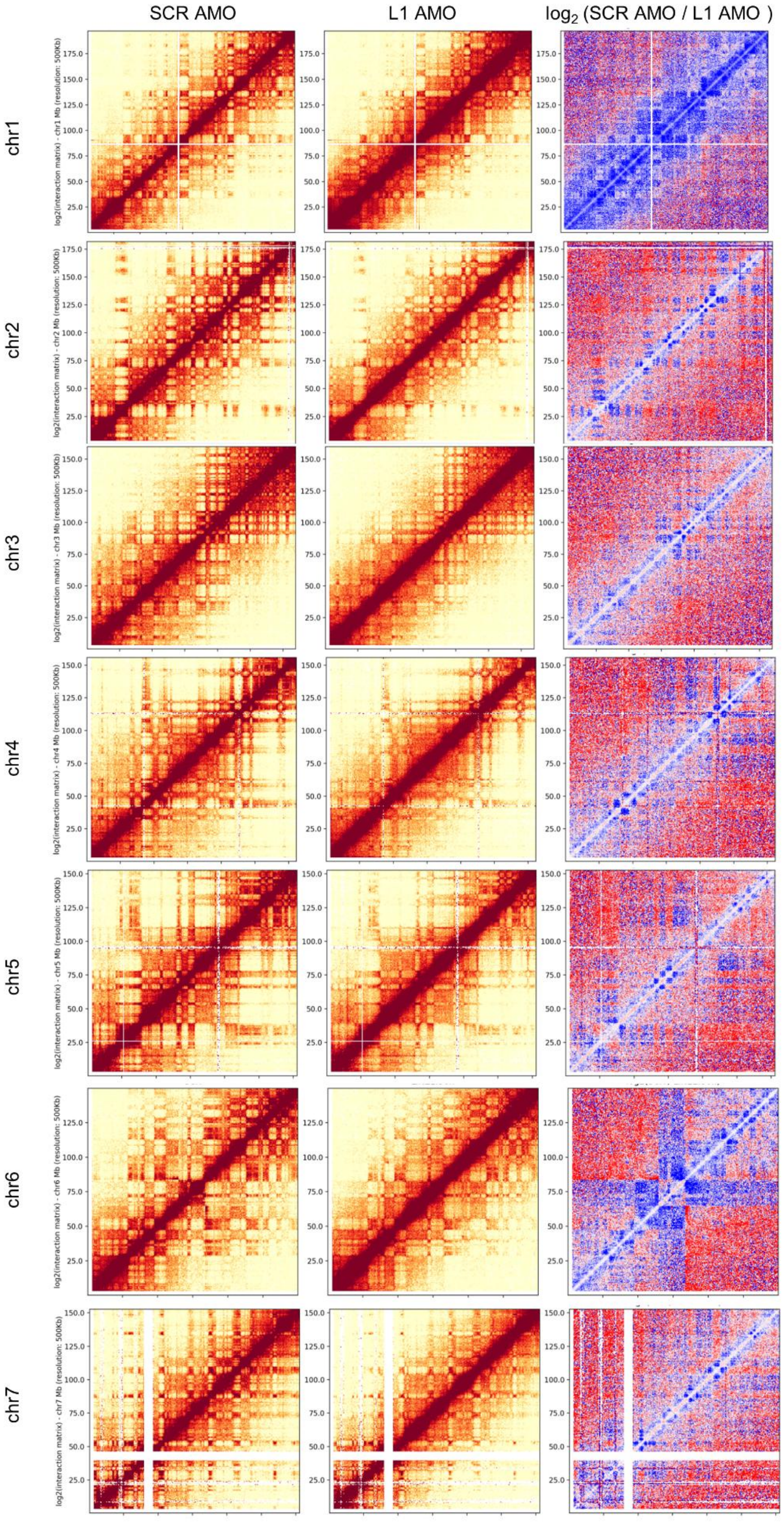

SCR AMO
L1 AMO
log2 (SCR AMO / L1 AMO)
chr1
chr2
chr3
chr4
chr5
chr6
chr7

Continued

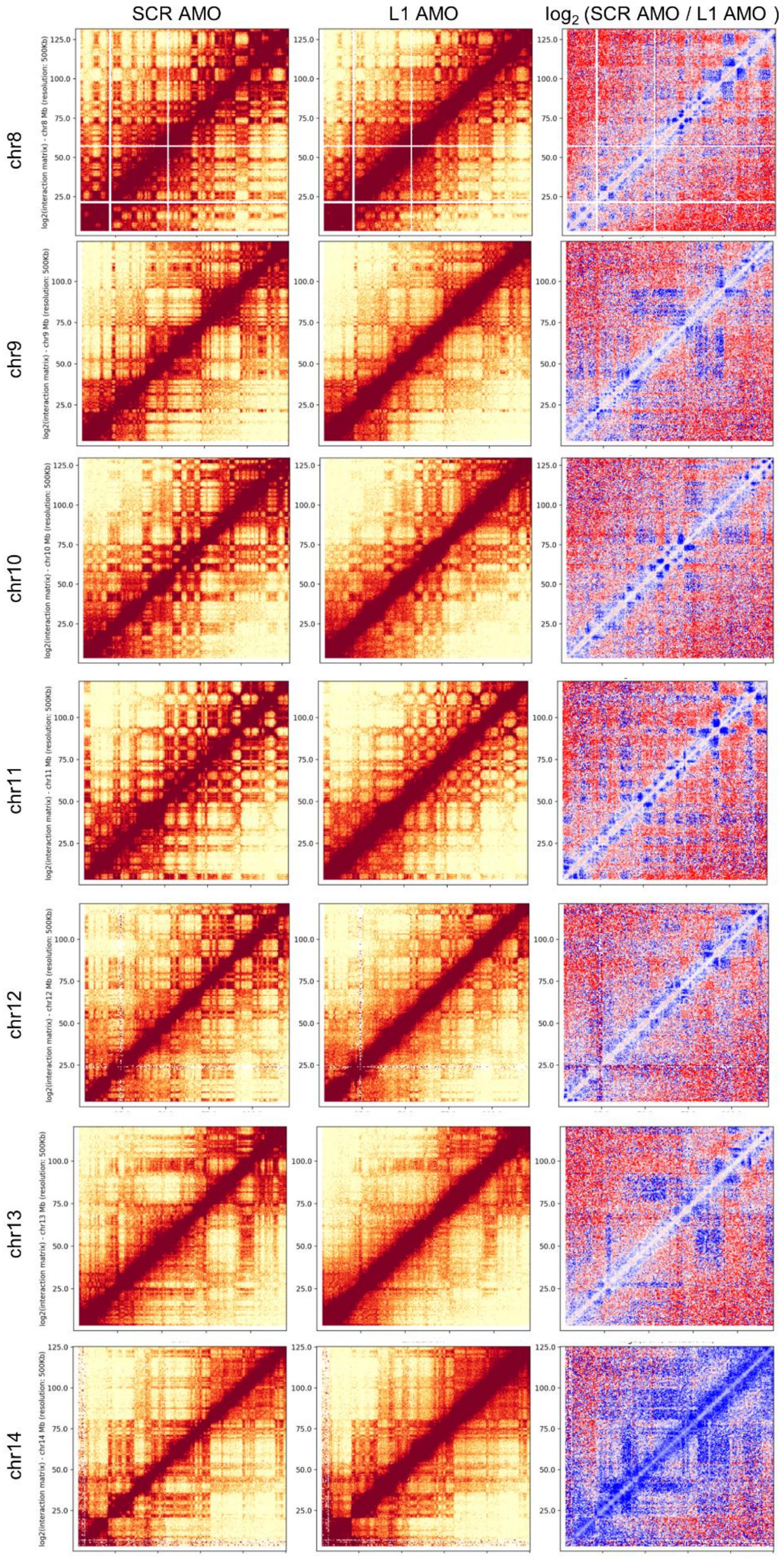

SCR AMO
L1 AMO
log$_2$ (SCR AMO / L1 AMO)
chr8
chr9
chr10
chr11
chr12
chr13
chr14

Continued

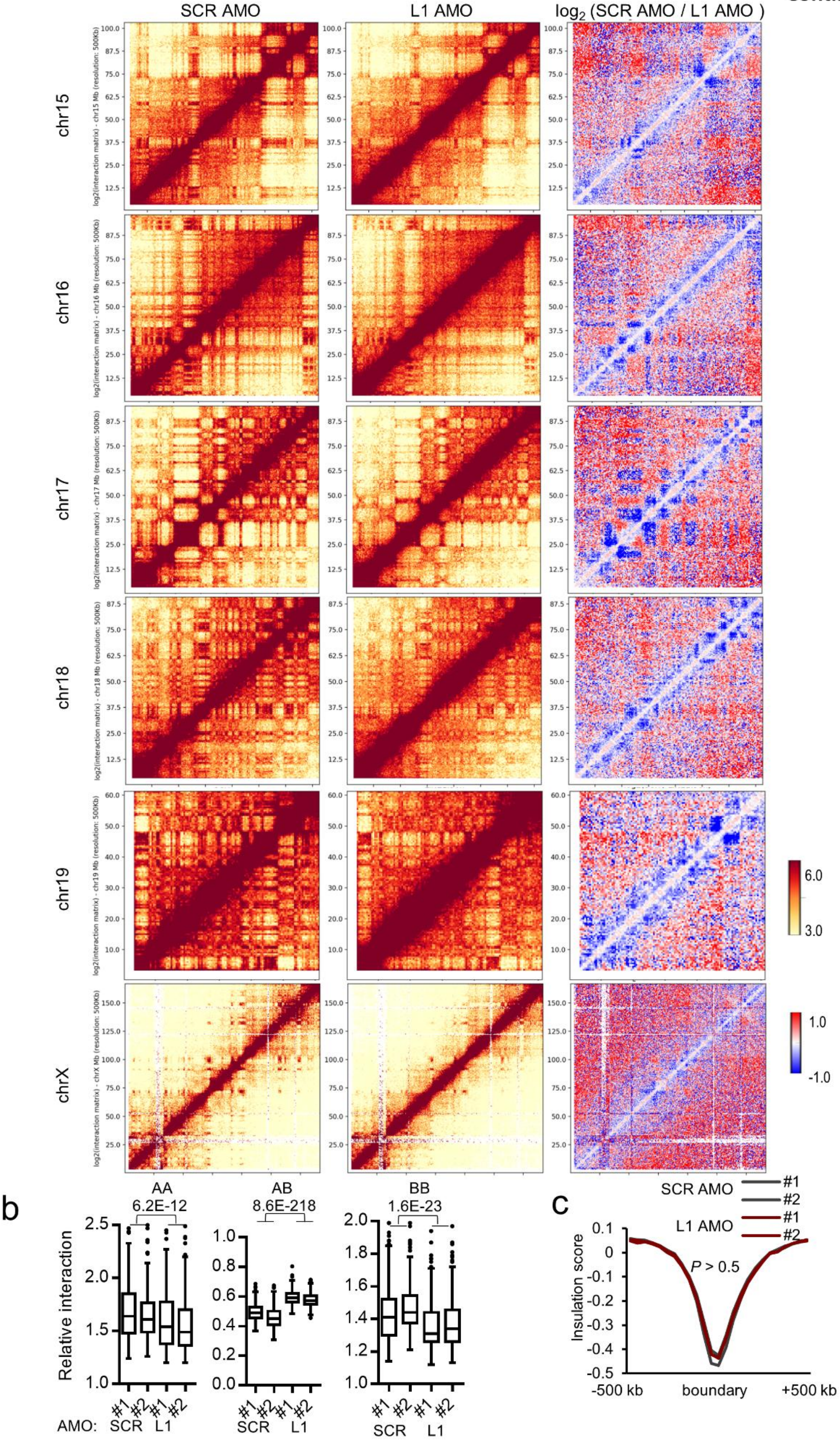

SCR AMO
L1 AMO
log2 (SCR AMO / L1 AMO)
chr15
chr16
chr17
chr18
chr19
chrX
6.0
3.0
1.0
-1.0
b
AA
6.2E-12
AB
8.6E-218
BB
1.6E-23
Relative interaction
#1 #2 #1 #2
AMO: SCR L1
c
SCR AMO #1 #2
L1 AMO #1 #2
Insulation score
P > 0.5
-500 kb
boundary
+500 kb

Fig. S9 L1 RNA is required for the formation and maintenance of higher-order chromatin structure.

(a) Hi-C analysis of mESCs treated with scramble (SCR) and L1 AMOs for 36 hours (pooled data from 2 biological replicates). Hi-C heatmaps show the contact frequencies for SCR and L1 AMO and the comparison of contact frequencies between SCR and L1 AMOs [$\log_2$(SCR/L1)]. All chromosomes are shown at 500-kb resolution.

(b) Analysis of compartment strength showing decreased compartmentalization in mESCs treated with L1 AMOs for 36 hours compared to SCR AMO. Boxplots of A-A, B-B, A-B interaction are shown. L1 AMO led to decreased A-A and B-B but increase of A-B interaction compared to SCR AMO. The *p* value was calculated by the two-tailed Student's *t*-test.

(c) Metagene analysis of insulation scores around the TAD boundary in mESCs treated with L1 or SCR AMO. Two biological replicates are shown.
