# Supplementary material for: Homotypic clustering of L1 and B1/Alu repeats compartmentalizes the 3D genome
Source: Cell Res. 2021 Jan 29;31(6):613–30. doi: 10.1038/s41422-020-00466-6 (PMC8169921; doi:10.1038/s41422-020-00466-6)
Supplement: Supplementary file 10 — Supplementary information, Figure S10 [file 41422_2020_466_MOESM10_ESM.pdf]

Fig. S10

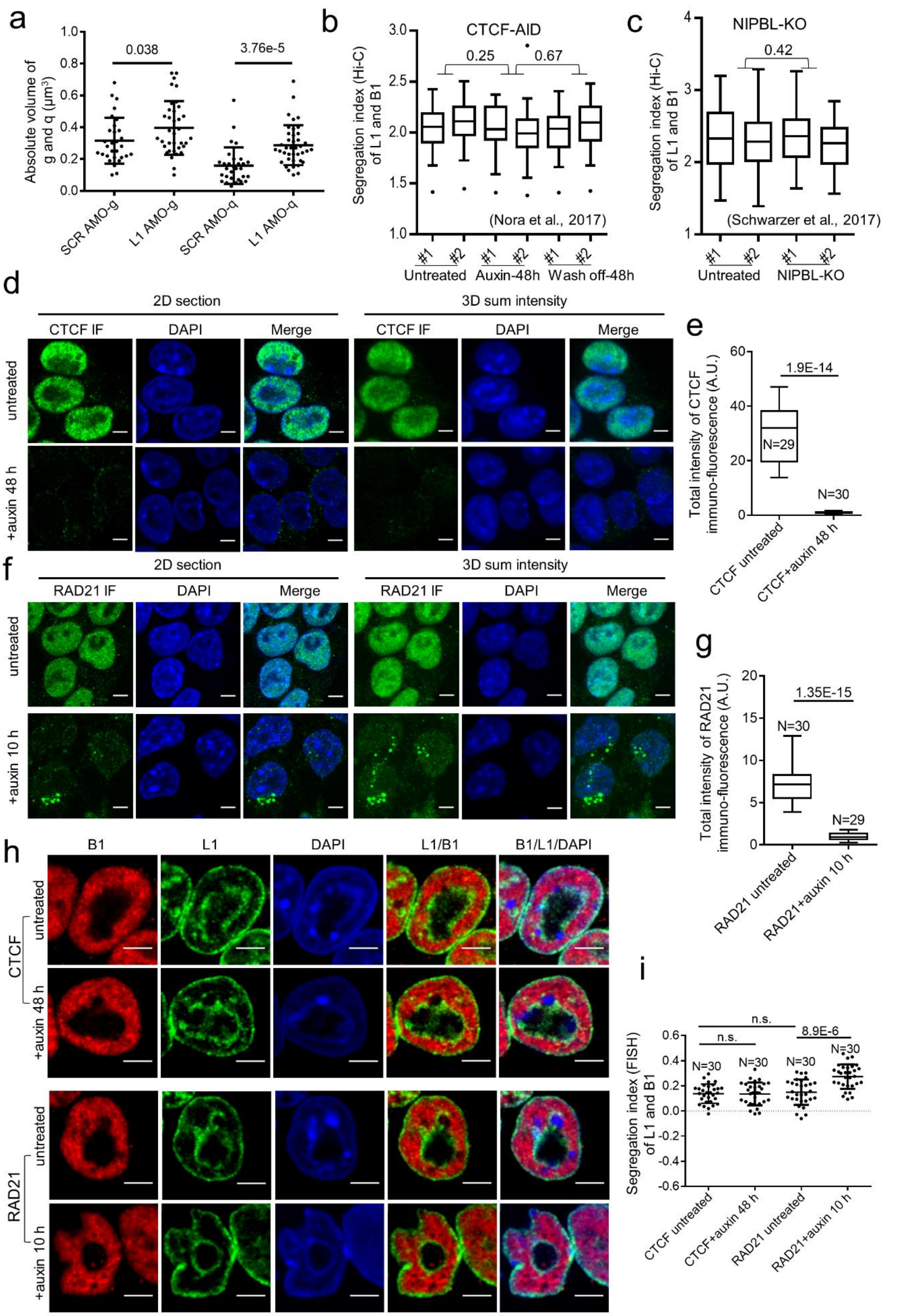

a
Absolute volume of g and q (μm³)
0.038
3.76e-5
SCR AMO-g
L1 AMO-g
SCR AMO-q
L1 AMO-q
b
CTCF-AID
Segregation index (Hi-C) of L1 and B1
0.25
0.67
(Nora et al., 2017)
#1
#2
Untreated
Auxin-48h
Wash off-48h
c
NIPBL-KO
0.42
(Schwarzer et al., 2017)
Untreated
NIPBL-KO
d
2D section
3D sum intensity
CTCF IF
DAPI
Merge
untreated
+auxin 48 h
e
Total intensity of CTCF immuno-fluorescence (A.U.)
1.9E-14
N=29
N=30
CTCF untreated
CTCF+auxin 48 h
f
RAD21 IF
+auxin 10 h
g
Total intensity of RAD21 immuno-fluorescence (A.U.)
1.35E-15
RAD21 untreated
RAD21+auxin 10 h
h
B1
L1
L1/B1
B1/L1/DAPI
CTCF
RAD21
i
Segregation index (FISH) of L1 and B1
n.s.
8.9E-6
CTCF untreated
CTCF+auxin 48 h
RAD21 untreated
RAD21+auxin 10 h

Fig. S10 Segregation of L1 and B1 is preserved upon depletion of CTCF or cohesin.

(a) Absolute volume of two B compartmental regions (*g* and *q*). The *p* value was calculated by the two-tailed Student's *t*-test. Related to Fig. 6b (panel ii).

(b) Boxplots showing the Hi-C segregation index of L1 and B1 in WT ESCs or ESCs depleted of CTCF. The *p* value was calculated by the two-tailed Student's *t*-test. The analysis was based on the published Hi-C data by Nora et al., 2017[4].

(c) Boxplots showing the Hi-C segregation index of L1 and B1 in WT ESCs or ESCs depleted of NIPBL. The *p* value was calculated by the two-tailed Student's *t*-test. The analysis was based on the published Hi-C data by Schwarzer et al., 2017[5].

(d) Immunofluorescence of CTCF in the ESCs with or without auxin treatment. The images of WT and ESCs treated with auxin are shown with the same imaging conditions and under the same intensity threshold. All scale bars, 5 μm.

(e) Total intensity of CTCF immunofluorescence (A.U.) in the ESCs with or without auxin treatment. The *p* value was calculated by the two-tailed Student's *t*-test.

(f) Immunofluorescence of RAD21 in the ESCs with or without auxin treatment. All scale bars, 5 μm. The images of WT and ESCs treated with auxin are shown with the same imaging conditions and under the same intensity threshold. All scale bars, 5 μm.

(g) Total intensity of RAD21 immunofluorescence (A.U.) in the ESCs with or without auxin treatment. The *p* value was calculated by the two-tailed Student's *t*-test.

(h) Representative images of L1 (green) and B1 (red) repeats revealed by DNA FISH in WT ESCs and ESCs depleted of CTCF or RAD21. DNA is labeled by DAPI (blue). All scale bars, 5 μm.

(i) Scatterplot of the segregation index of L1 and B1 DNA in (e). The *p* value was calculated by the two-tailed Student's *t*-test.

References:

4. Nora, E. P. *et al.* Targeted Degradation of CTCF Decouples Local Insulation of Chromosome Domains from Genomic Compartmentalization. *Cell* **169**, 930-944, doi:10.1016/j.cell.2017.05.004 (2017).
5. Schwarzer, W. *et al.* Two independent modes of chromatin organization revealed by cohesin removal. *Nature* **551**, 51-56, doi:10.1038/nature24281 (2017).
