# Supplementary material for: Homotypic clustering of L1 and B1/Alu repeats compartmentalizes the 3D genome
Source: Cell Res. 2021 Jan 29;31(6):613–30. doi: 10.1038/s41422-020-00466-6 (PMC8169921; doi:10.1038/s41422-020-00466-6)
Supplement: Supplementary file 11 — Supplementary information, Figure S11 [file 41422_2020_466_MOESM11_ESM.pdf]

Fig. S11

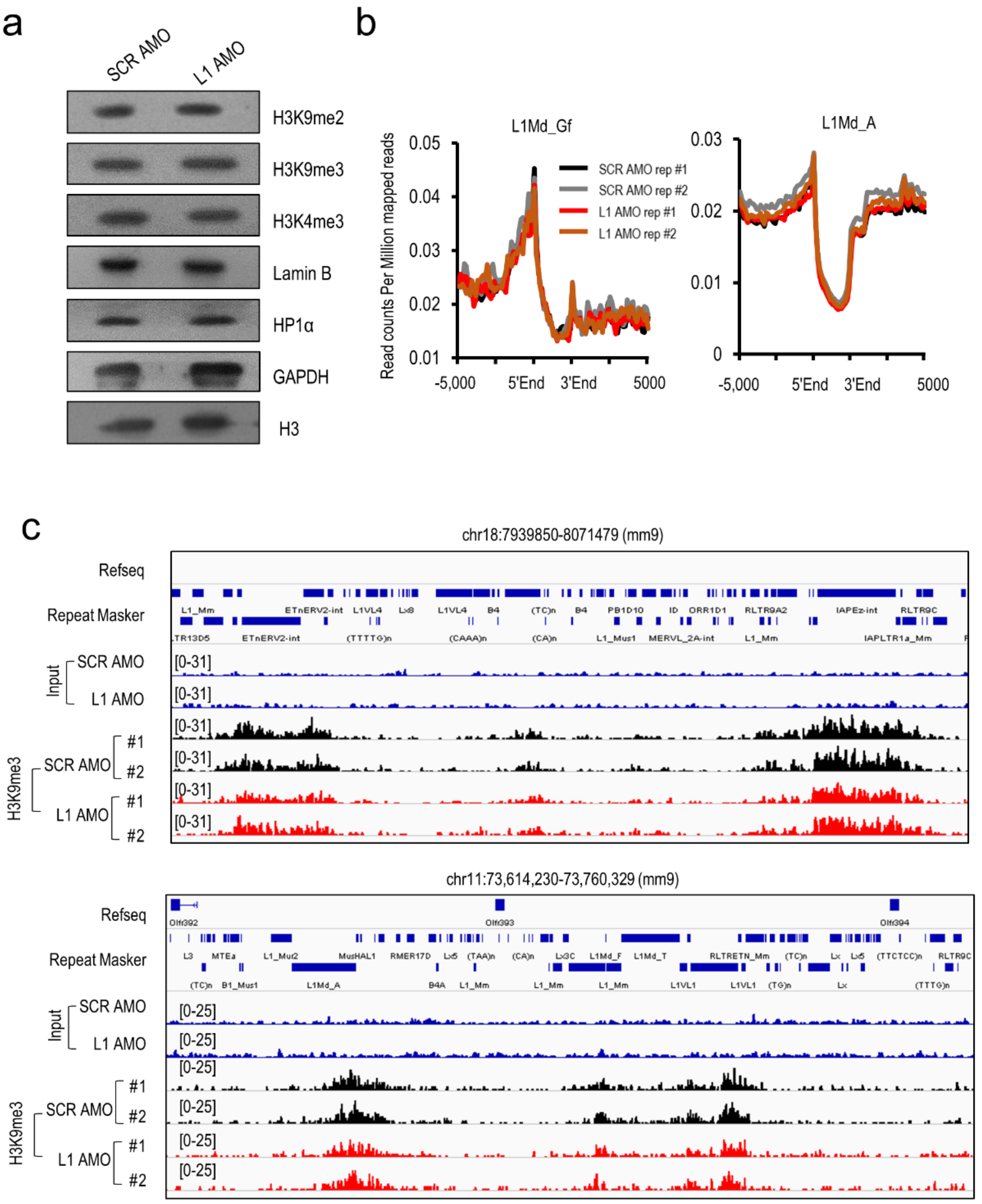

a
SCR AMO
L1 AMO
H3K9me2
H3K9me3
H3K4me3
Lamin B
HP1α
GAPDH
H3
b
L1Md_Gf
L1Md_A
Read counts Per Million mapped reads
SCR AMO rep #1
SCR AMO rep #2
L1 AMO rep #1
L1 AMO rep #2
0.05
0.04
0.03
0.02
0.01
0
-5,000
5'End
3'End
5000
c
chr18:7939850-8071479 (mm9)
Refseq
Repeat Masker
Input
SCR AMO
L1 AMO
H3K9me3
#1
#2
[0-31]
chr11:73,614,230-73,760,329 (mm9)
Olfr392
Olfr393
Olfr394
[0-25]

Fig. S11 Heterochromatic histone mark H3K9me3 is preserved upon depletion of L1 RNA.

(a) Western blot analysis of histone modification mark, HP1α and Lamin B after depletion of L1 RNA in mESCs.
(b) Metagene analysis of H3K9me3 ChIP-seq signal around the L1Md_Gf and L1_A family in mESCs treated with L1 or SCR AMO. Two biological replicates are shown.
(c) Genome browser view of H3K9me3 ChIP-seq signal tracks in two regions in mESCs. Annotations of Refseq gene and Repeat Masker are shown in the first and second row, respectively.
