# Supplementary material for: Homotypic clustering of L1 and B1/Alu repeats compartmentalizes the 3D genome
Source: Cell Res. 2021 Jan 29;31(6):613–30. doi: 10.1038/s41422-020-00466-6 (PMC8169921; doi:10.1038/s41422-020-00466-6)
Supplement: Supplementary file 12 — Supplementary information, Figure S12 [file 41422_2020_466_MOESM12_ESM.pdf]

Fig. S12

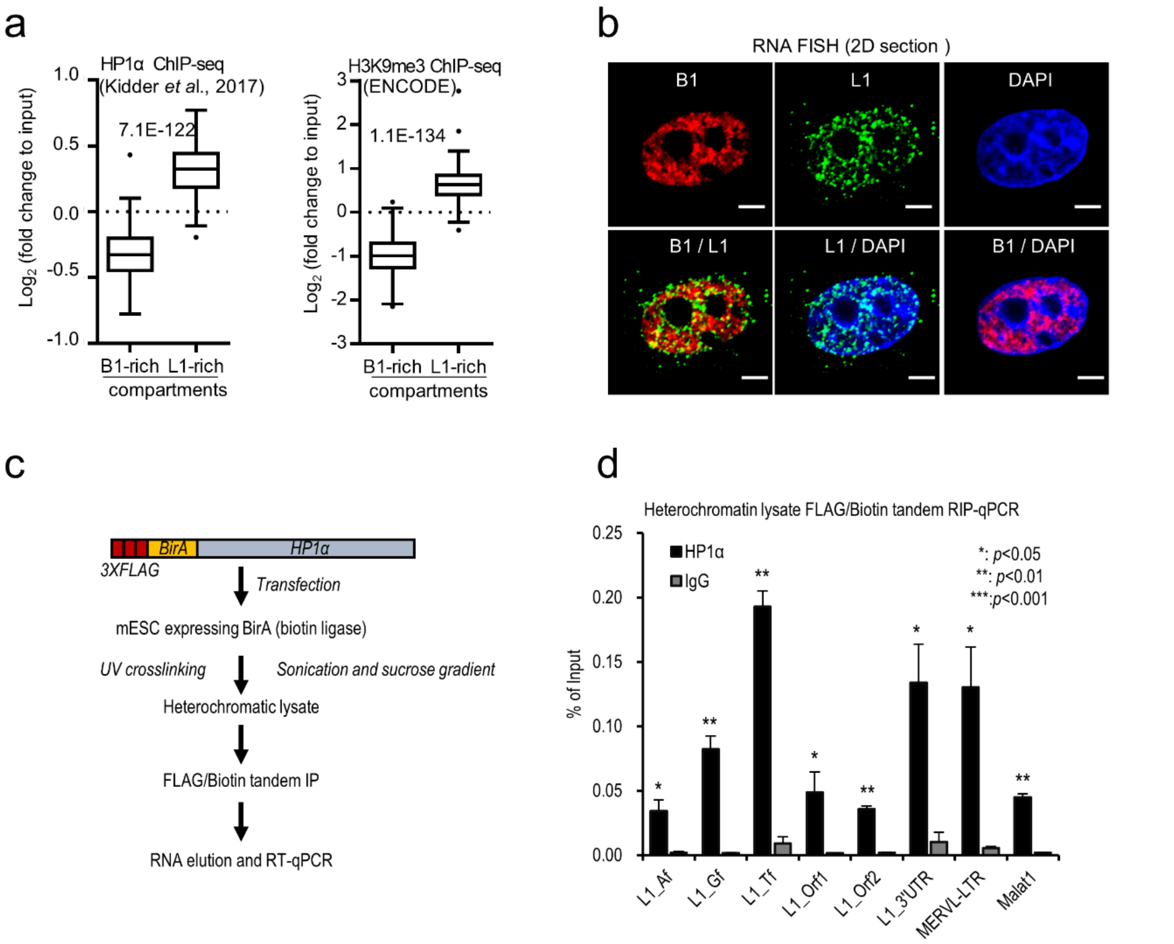

a
HP1α ChIP-seq
(Kidder et al., 2017)
7.1E-122
Log2 (fold change to input)
1.0
0.5
0.0
-0.5
-1.0
B1-rich L1-rich
compartments
H3K9me3 ChIP-seq
(ENCODE)
1.1E-134
3
2
1
0
-1
-2
-3
B1-rich L1-rich
compartments
b
RNA FISH (2D section)
B1
L1
DAPI
B1 / L1
L1 / DAPI
B1 / DAPI
c
BirA
HP1α
3XFLAG
Transfection
mESC expressing BirA (biotin ligase)
UV crosslinking
Sonication and sucrose gradient
Heterochromatic lysate
FLAG/Biotin tandem IP
RNA elution and RT-qPCR
d
Heterochromatin lysate FLAG/Biotin tandem RIP-qPCR
HP1α
IgG
*: p<0.05
**: p<0.01
***:p<0.001
% of Input
0.25
0.20
0.15
0.10
0.05
0.00
L1_Af
L1_Gf
L1_Tf
L1_Orf1
L1_Orf2
L1_3'UTR
MERVL-LTR
Malat1

Fig. S12 HP1α bindings in L1 and B1-rich compartments and RNA FISH of L1 and B1.

(a) Boxplot showing the ChIP-seq signal of HP1α and H3K9me3 in B1- and L1-rich compartments. Y-axis showing the fold change of raw ChIP-seq signal to input DNA. *p* values are calculated with two-tailed Student's *t*-test.

(b) RNA FISH analysis of L1 (green) and B1 (red) RNA in mESCs. Representative images are presented. Scale bars, 5 μm.

(c) Schematic illustration of RIP-qPCR of HP1α tagged by 3x FLAG and biotin moieties.

(d) RIP-qPCR of HP1α. IgG was shown as a control. *p* values are calculated with two-tailed Student's *t*-test.
