# Supplementary material for: Homotypic clustering of L1 and B1/Alu repeats compartmentalizes the 3D genome
Source: Cell Res. 2021 Jan 29;31(6):613–30. doi: 10.1038/s41422-020-00466-6 (PMC8169921; doi:10.1038/s41422-020-00466-6)
Supplement: Supplementary file 13 — Supplementary information, Figure S13 [file 41422_2020_466_MOESM13_ESM.pdf]

Fig. S13

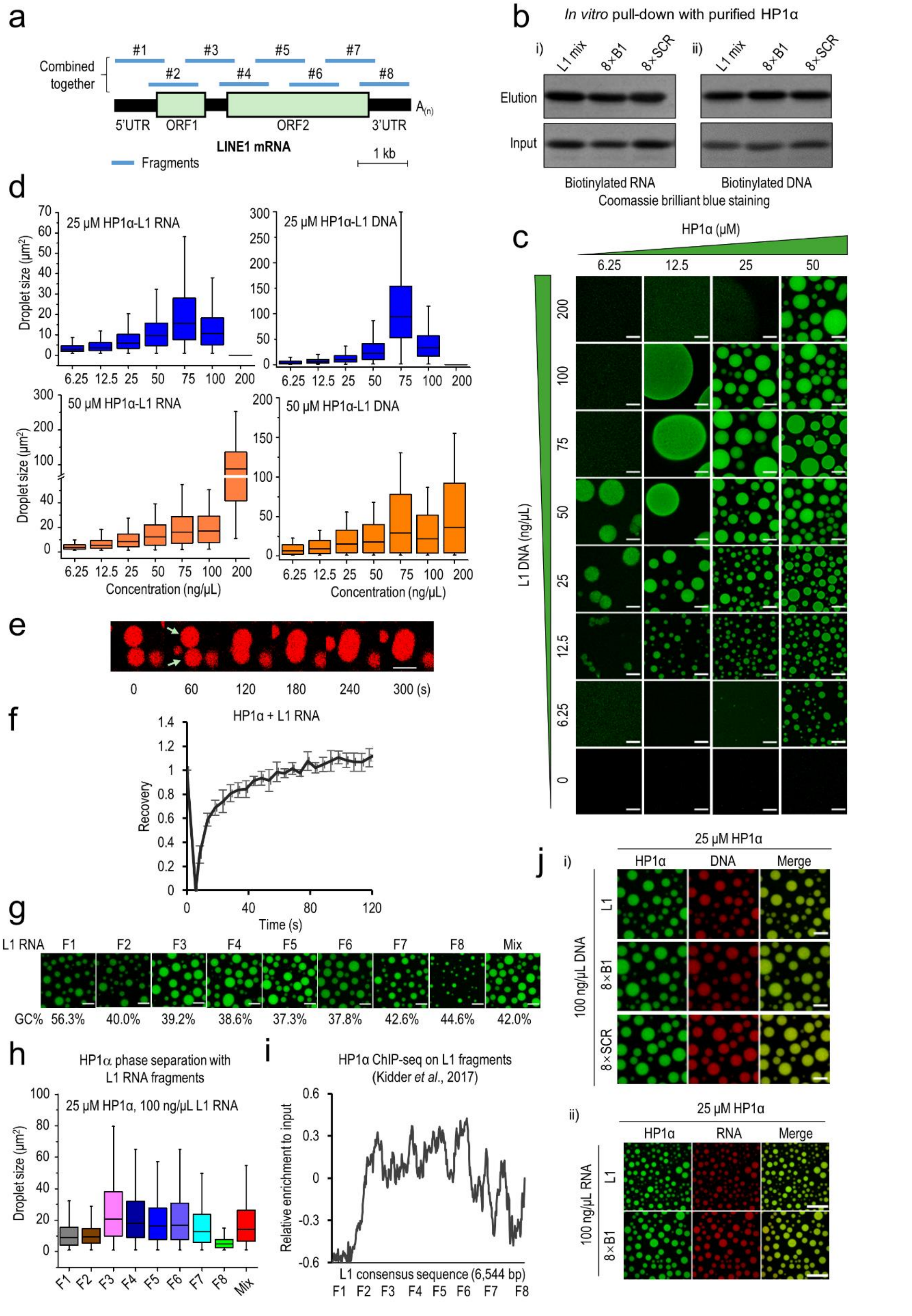

a
Combined together
#1 #2 #3 #4 #5 #6 #7 #8
5'UTR ORF1 ORF2 3'UTR
LINE1 mRNA
Fragments
1 kb
b
In vitro pull-down with purified HP1α
i) L1 mix 8×B1 8×SCR
ii) L1 mix 8×B1 8×SCR
Elution
Input
Biotinylated RNA
Biotinylated DNA
Coomassie brilliant blue staining
c
HP1α (μM)
6.25 12.5 25 50
L1 DNA (ng/μL)
200 100 75 50 25 12.5 6.25 0
d
25 μM HP1α-L1 RNA
25 μM HP1α-L1 DNA
50 μM HP1α-L1 RNA
50 μM HP1α-L1 DNA
Droplet size (μm²)
6.25 12.5 25 50 75 100 200
Concentration (ng/μL)
e
0 60 120 180 240 300 (s)
f
HP1α + L1 RNA
Recovery
Time (s)
g
L1 RNA F1 F2 F3 F4 F5 F6 F7 F8 Mix
GC% 56.3% 40.0% 39.2% 38.6% 37.3% 37.8% 42.6% 44.6% 42.0%
h
HP1α phase separation with L1 RNA fragments
25 μM HP1α, 100 ng/μL L1 RNA
Droplet size (μm²)
F1 F2 F3 F4 F5 F6 F7 F8 Mix
i
HP1α ChIP-seq on L1 fragments (Kidder et al., 2017)
Relative enrichment to input
L1 consensus sequence (6,544 bp)
F1 F2 F3 F4 F5 F6 F7 F8
j
i) 25 μM HP1α
HP1α DNA Merge
100 ng/μL DNA
L1 8×B1 8×SCR
ii) 25 μM HP1α
HP1α RNA Merge
100 ng/μL RNA
L1 8×B1

Fig. S13 HP1α binds and phase separates with RNA and DNA.

(a) Schematic illustrations of L1 fragments used for interaction and phase separation analysis with HP1α.

(b) *In vitro* RNA and DNA pull-down of HP1α.

(c) Representative images of droplet formation at different concentrations of HP1α protein and L1 DNA. Concentrations of HP1α and DNA are indicated at the top and left of the images, respectively. Scale bars, 10 µm. Data are representative of 3 independent experiments.

(d) Boxplot showing the size of droplets formed by HP1α and L1 RNA/DNA.

(e) Fluorescence time-lapse microscopy of HP1α-L1 RNA droplets. Two fusing bodies are indicated by arrows. Scale bars, 10 µm. Data are representative of 5 independent experiments.

(f) FRAP of HP1α-L1 RNA droplets. The plot shows the time course of the recovery after photobleaching. Time 0 indicates the time of the photobleaching pulse. Scale bar, 10 µm. Data are presented as means ± SD (N = 6). RNA concentration: 50 ng/µl; HP1α concentration: 25 µM.

(g) Representative images of droplet formation of HP1α protein and L1 RNA fragments shown in (a). GC contents of L1 RNA fragment were shown at the bottom.

(h) Boxplot showing the size of droplets formed by HP1α and L1 RNA fragments shown in (g).

(i) HP1α preferentially binds to the central region of L1 repeat DNA. Relative enrichments of HP1α ChIP-seq signals normalized to the input (y-axis) are shown across the consensus L1 element (6,544 bp; from F1 to F8), based on HP1α ChIP-seq in mESCs reported by Kidder et al. (2017)[6].

(j) Representative images of droplet formation of HP1α protein with various DNA (upper: L1, 8xB1 and 8xSCR) and RNA fragments (lower: L1 and 8xB1).

Reference

6. Kidder, B. L., Hu, G., Cui, K. & Zhao, K. SMYD5 regulates H4K20me3-marked heterochromatin to safeguard ES cell self-renewal and prevent spurious differentiation. *Epigenetics Chromatin* **10**, 8, doi:10.1186/s13072-017-0115-7 (2017).
