# Supplementary material for: Homotypic clustering of L1 and B1/Alu repeats compartmentalizes the 3D genome
Source: Cell Res. 2021 Jan 29;31(6):613–30. doi: 10.1038/s41422-020-00466-6 (PMC8169921; doi:10.1038/s41422-020-00466-6)
Supplement: Supplementary file 19 — Supplementary information, Text S1 [file 41422_2020_466_MOESM19_ESM.pdf]

Supplementary information, Text S1 Supplemental Discussion.

Organization of the genome into euchromatin and heterochromatin has been known for over 90 years[1]. Tremendous efforts have been dedicated to studies of structural chromatin proteins and cataloging chromatin maps. Despite our understanding of how the genome is organized in different hierarchical scales, the basic principle of its formation remains unknown. Intriguingly, the overall genome organization, including TADs and most A/B compartments, seems to be *invariant* in different mouse and human cells at both bulk and single-cell levels[2-9]. Yet, the mechanisms of compartmental formation continue to be unresolved.

Apart from studies of chromatin proteins, we have taken a different perspective by considering the possibility that the structural information may be embedded in the genome itself. In this paper, we show a nearly perfect correlation of L1 and B1 distributions in the genome with the 3D folding (particularly at the compartmental level), and reveal the essentiality of L1 RNA in promoting homotypic repeat clustering and proper compartmentalization. The collective evidence based on genomics and experimental demonstrations suggests a *functional* role for L1s in driving homotypic repeat clustering, compartmental segregation and formation of the higher-order genome structure, rather than a mere compartmental marker. The role of B1/Alu RNA in genome folding requires future exploration. Nevertheless, the abundance (~30%) and widespread scattering nature of L1 and B1/Alu repeats in the mouse and human genomes render them a unique advantage to perform such a task as the genomic *blueprint* to organize the genome structure and function[10]. Considering transcription and epigenetic machineries have to act above and beyond DNA sequence, the structural information provided by repeat DNA should be the most *rudimental* as it instructs transcription activities and *de novo* establishment of histone marks, which in turn provide feedback on genome folding.

However, the full revelation of the causal relationship remains a challenge (perhaps it is impossible with current technology to dissect such a complex phenomenon as genome folding without getting into a bit of circular logic). Future investigations are also required to determine the underlying mechanisms by which these repeats function in detail. Nonetheless, our results have introduced an entirely novel concept of L1 and B1/Alu repeats in organizing the 3D genome, which will take the field in an electrifying new direction. To put our findings in the context of current knowledge in the field, here we summarize and cite relevant data figures that were reported in the literature related to two aspects, including 1) the conservation of 3D genome across human and mouse; 2) L1 and B1 distributions and their correlation to the A/B compartments.

Part 1. Summary of representative reports related to the conservation of 3D genome folding.

1. Genome portioning into TADs is largely stable across cell types in mouse and human.

The Ren group (2012) first proposed the topologically associated domains (TAD) as a megabase-sized local chromatin interaction domain of metazoan genomes[2]. Their initial analysis of 4 cell types including mouse and human ESCs, mouse cortex and human lung fibroblast (IMR90), revealed that most of TAD boundaries are shared and largely invariant between cell types[2]. They concluded that “the topological domains we identified are well conserved between mice and human”, and suggested that “the sequence elements and mechanisms that are responsible for establishing higher order structures in the genome may be relatively ancient in evolution”. The observation that chromosomes are composed of cell-type-invariant TADs was further confirmed by Hi-C analysis of a broad range of cell types and during dynamic biological processes such as stem cell differentiation and somatic cell reprogramming[3-5]. In the paper by Dixon and Ren *et al.* (2015), they stated that “although the positioning of TADs remains stable between cell types, numerous changes in chromatin structure occur within domains” and “a subset of TADs in a given lineage undergo concerted, domain-wide changes in interaction frequency”. In the paper by Schmitt and Ren *et al.* (2016), they identified a total of 3,010 distinct TAD boundaries in 21 human tissues and cell lines and reported that “TAD boundaries

are indeed highly conserved across cell lines and tissues". Stadhouders and Graf *et al.* (2018) studied 3D genome organization during cell reprogramming from fully differentiated B lymphoids to pluripotent stem cells[5]. They showed that only a minor portion of TAD borders were altered during reprogramming; however, "quantitative aspects of TADs, namely their connectivity and insulation potential, are subject to substantial changes during reprogramming and therefore are more cell-type specific in nature". Given the general consensus that TADs are cell-type invariant, evolutionarily conserved in a broad spectrum of cell types across species, TADs have been considered as the basic units of chromosome folding and regarded as an important secondary structure in chromosome organization[11,12].

2. Analysis of chromatin looping has revealed substantial conservation of 3D genome structure across mammals.

Rao and Aiden *et al*. (2014) generated dense 3D maps at kilobase resolution in nine human and mouse cell lines representing all germ layers, and revealed substantial conservation of 3D genome structure across mammals[6]. As they stated in the paper, contact domains (mean length:220 kb; similar to TADs), which exhibit consistent histone marks, are often preserved across cell types. By focusing on chromatin loops, they showed that between 55% and 75% of peaks (loci with high contact frequency) found in any given cell type tested were also found in a representative human cell type—GM12878 B-lymphoblastoid, which was studied with the densest Hi-C contacts in a resolution of 950 bp. Further examination of orthologous regions across species showed that 50% of peaks and 45% of domains called in mouse were also called in humans.

3. Conservation of genome organization at the compartmental level.

Using Hi-C, the Dekker group (2009) reported that the genome is organized by the spatial segregation of open and closed chromatin to form two genome-wide compartments, which were arbitrarily labeled A and B and are correlated with transcriptional activity[7]. Although the high degree of conservation in TADs is generally accepted, compartmental conservation remains a matter of controversy in the field.

Dixon and Ren et al. (2015) reported "extensive A/B compartment switch" across six human cell lines, as 36% of the genome switched compartments in at least one of the lineages analyzed, including hESCs, four hESC-derived lineages, and IMR90 fibroblasts. In fact, according to their Extended Data Figure 2c (right), only 3.8% to 25% of the genome switched compartments in any given cell type compared to hESCs. This indicates that the most majorities (75%~96%) of the genome show no change at the compartment level between cell types.

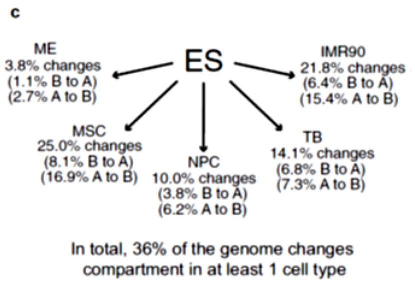

Cited from Extended Data Fig. 2c of Dixon *et al.*, 2015, Nature.
Percentage of the genome that changes A/B compartment upon differentiation of ES cells into each of the five differentiated lineages.

Schmitt and Ren et al. (2016) analyzed Hi-C maps in a large set (21) of human cells, including 14 tissues and 7 cell lines[4]. They also reported "substantial compartment A/B switching across primary tissues" as indicated by 59.6% of the genome that was dynamically compartmentalized, despite high conservation of TAD boundaries across different cells. However, Figure 1C and 1E show a similar ratio (40%) of the genome with conserved TAD boundary region or showing conserved compartments [25% for B (21B) and 15% for A (21A)] in all 21 tissues. Given this similar degree of conservation observed in TADs and compartments, it is puzzling how completely different conclusions were reached: TAD is indeed conserved, whereas compartment is substantially dynamic? In addition, re-analysis of Hi-C results in their Extended Data Fig. 2 showed that ~40% of the genome has invariant compartments in all 21 tissues and cell types (consistent with their result in Figure 1C), and ~74% are invariant in at least 17 distinct samples, and ~80% in >16 samples

(please see our Extended Data Fig. 2). This degree of compartment conservation is highly significant, considering that, by chance, only 0% of the genome in all 21 tissue, 3% in >17 tissues, and 7% in >16 tissues are expected to remain unchanged of compartmental status (chi-square test p value < 2.2E-16).

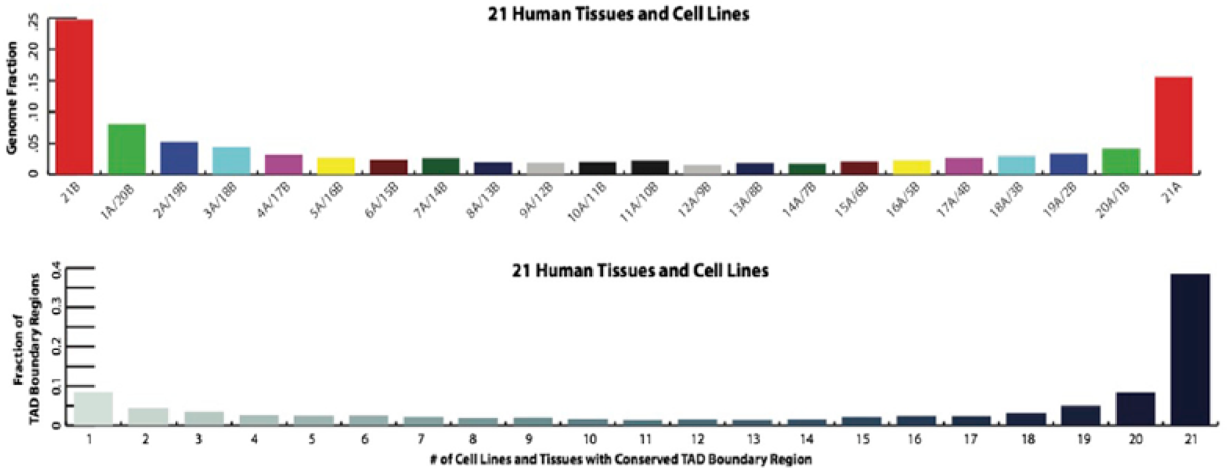

Cite from Figure 1C and 1E of Schmitt *et al*., 2016. Cell Reports
Upper: (C) Bar plots showing the degree of conservation of A/B compartment labels of 21 human cell lines and adult tissues. The y axis is the fraction of the genome conserved by the 22 possible combinations of compartment A/B designations. The label below each bar represents the composition of the compartment designations. For example, "16A/5B" represents the genomic regions where 16 samples exhibit a compartment A label and the other five samples exhibit a compartment B label.
Lower: (E) Bar plots showing the degree of TAD boundary conservation across 21 human cell lines and tissues. For each putative boundary region, we tallied how many samples have a boundary within that region. The y axis is the fraction of TAD boundaries conserved at least a certain number of samples, as categorized along the x axis.

Furthermore, one caveat of compartmental analysis of a region is that compartment assignment was determined by continuous PC1 values as the first principal component to compartments A and B of a principal components analysis (PCA) based on Hi-C. Positive PC1 correspond to compartment A, and negative PC1 correspond to compartment B, while regions with a PC1 of zero often correspond to centromeric and telomeric regions of the chromosomes[3]. Note that the absolute value of PC1 represents the statistic likelihood of a region to be associated with compartment A or B. A high absolute value of PC1 means more accurate when a compartment is designated as either A or B. For regions with low absolute PC1 values (closer to zero), their compartmental assignment may not be reliable due to random variation. We found that regions showing frequent compartmental switches, including variable A compartment in those conserved B regions (as designated as 1A/20B, 2A/19B,3A/18B, 4A/17B, 5A/16B etc. in the paper) and variable B in those conserved A (20A/1B, 19A/1B, 18A/3B, 17A/4B, 16A/5B, etc.), tend to have a lower absolute value of PC1 that is closer to zero. These "heretic" changes should be interpreted with caution. Because of their less pronounced compartmental associations at the beginning, the assessment of their changes in compartments between cells may not be reliable, which may lead to an over-estimation of compartmental changes.

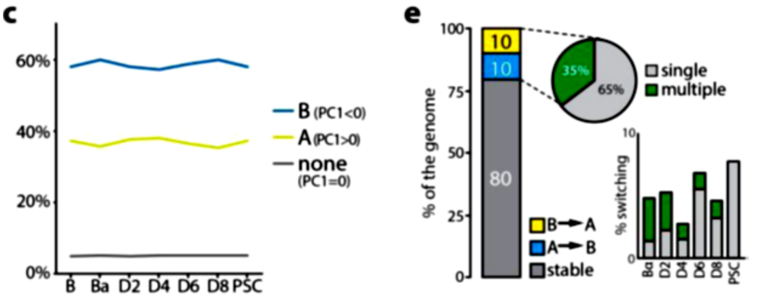

Cited from Supplementary Fig 2c and 2e of Stadhouders *et al.,* 2018, Nature Genetics.
(c) Line chart depicting genome fractions assigned to A or B compartments at the different time points. Regions that could not be assigned (PC1=0, e.g. telomeric regions) are shown in gray.
(e) Fraction of the genome that switches compartment at any point during the time course. Bar graph depicts switching percentages per timepoint.

Stadhouders and Graf *et al.* (2018) reported that the overall proportions assigned to A and B compartments (40% to A and 60% to B) remained unchanged and only 20% of the genome switched compartments throughout reprogramming, although compartmentalization strength was dynamically altered. They made an important note that compartment switching typically occurred in regions with low PC1 values (statistically less reliable) at the edges of A- or B-compartment domains. As Stadhouders *et al.* indicated in the paper, loci with a less pronounced compartment association are more likely to change their compartment status. In this regard, the calculation of ~20% of the genome undergoing compartmental switches might be overly estimated. Altogether, these results indicate that **compartmental organization is relatively stable across different lineages, from top to bottom Waddington landscape for cell fate.**

Moreover, single-cell Hi-C and microscopic analyses of individual mammalian genomes have reported that compartments A and B in a spatially polarized organization are relatively stable and present in individual cells, despite single-cell 3D genomes are intrinsically stochastic and dynamic[9,13-17]. For example, in the paper by Stevens and Laue *et al.* (2017), they showed that A and B compartments are organized in a consistent way on a genome-wide basis in all examined 8 cells. Although the structures of individual TADs and loops vary markedly from cell to cell, regions belonging to the A or B compartments always cluster together and A segregates from B. Structural simulation based on Hi-C revealed that in all cells, the chromosomes pack together to give an outer B compartment ring, an inner A compartment right, and an internal B compartment region around the hollow nucleoli.

Furthermore, Rennie and Andersson *et al.* (2018) characterised predictions of 3D genome structure in 76 human cell types, observing largely invariant chromatin compartments between cells[8]. They found that cell-type identities are governed by differences in enhancer and promoter interactions or position-independent effects.

Lastly, we want to note that the observation of compartmental conservation does not conflict with the idea of dynamic gene regulation across cells. First, considering that non-coding DNA (repeats, regulatory elements, and noncoding RNA gene) greatly outnumbers coding DNA (producing mRNA and protein; <2%) in both human and mouse genomes, we reason that genic regions showing changes in transcriptional state in a cell may only account for a very small proportion of the genome. Second, although gene expression is correlated with its compartmental association, most of expression changes cannot be explained by compartmental switching. It was reported that 75% of all mRNAs in human and mouse are ubiquitously expressed in different levels in most tissues[18]. This suggests that most majorities of gene expression changes occur in the active compartment and are pertinent to up-and-down regulation, although there are cases that involves on-and-off and compartmental switches, such as the lncRNA ThymoD[19]. For developmental genes that do show on-and-off switches during cell-fate transition or in response to external signal, they also appear to reside in active compartments and are regulated through poised transcriptional activation by bivalent histone marks[20,21]], rather than switching their compartmental status. For example, we find that the HOXA cluster of genes that are only activated in specific cell types appears to reside in A compartments in 18 of 21 tissues, including ESCs (data not shown). During heat shock, despite dramatic transcriptional activation in hundreds of genes, there are no

significant changes in compartmentalization[22]. Consistent with these observations, we find that ~72% of human and mouse genes, including B1/Alu-rich genes with house-keeping activities and genes associated with simple repeats, appear to be located in A compartments in mESCs[10]. In comparison, genes in B compartments are mostly L1-associated genes that are strongly enriched in highly specialized functions, including olfactory, vomeronasal and pheromone receptor activities, immunoglobulin function and retinol metabolism, and tend to be expressed in terminally differentiated cells[10]. Moreover, evidence suggests decoupled transcriptional activation and changes in higher chromatin structures. For example, although transcriptional activation is sufficient to alter 3D genome organization, gene positioning and chromatin folding, these changes could occur independently of transcription[23-26]. Only a subset of genes appeared to be affected by compartmental changes during stem cell differentiation, whereas most genes remained unaffected[3]. During cell reprogramming, the gain or loss of TAD borders also did not correlate with overall increased or decreased local gene expression, respectively[5]. In the example of olfactory receptor genes[27], despite there are ~1,100 receptor genes that tend to form large gene clusters in the mouse genome and be sequestered in B compartments, one neuron expresses only one receptor, arguing against extensive compartmental switches from B to A in any given cell.

Taken together into a coherent theme, we believe compartments are stable in a genome-wide scale despite occasional switches in estimated 3~25% of the genome of analyzed cells. Consistent folding of the most majorities of the genome in different cells governs common transcriptional and nuclear activities through the same mechanisms and machineries. By sticking to a fundamental principle, the chromatin copes with cellular signals with more exquisite changes in specific genomic regions. This macroscopic stability coupled with local dynamics in genome organization appears to be both economic and effective in ensuring the robustness yet specificity in the function and regulation of individual genomes across diverse cell types.

Part 2. Evidence related to L1/B1 repeats and chromatin compartments

Although initial reports have suggested compartmental distributions of different repeats, these lines of evidence were fragmented and hidden in the literature. L1 and B1 repeats have not yet been explicitly implicated in chromatin organization, not even mentioning experimental testing, prior to our study.

1. Sequencing-based evidence

1) Initial analysis of human and mouse genomes revealed that L1s are enriched in gene-poor and AT-rich regions, whereas SINE elements are enriched in gene-rich and GC-rich genomic regions[28,29]. This observation was further confirmed by several studies[30-33].

2) Bas van Steensel group utilized DamID technology to reveal DNA sequences in lamina-associated domain (LAD) in fly, mouse and human cells [33-36]. They reported that constitutive LADs (cLADs) are evolutionarily conserved between human and mouse and contribute to a basal chromosome architecture. LADs are characterized by low gene activity (reminiscent of compartment B), and are enriched in LINE elements but depleted in B1/Alu. However, it was never discussed or tested whether LINE elements may regulate the positioning of cLADs (please see the original figure panels on the right). Importantly, chromatin segregation remains intact in naturally inverted nuclei of nocturnal rod photoreceptors that lack nuclear peripheral tethers[37] . Removal of major tethers of LADs, including all lamins or lamin B receptor LBR, have no detectable effects on the genome-wide interaction pattern of

Cited from Meuleman and Steensel *et al.*, 2013. Genome Research "Constitutive nuclear lamina–genome interactions are highly conserved and associated with A/T-rich sequence".

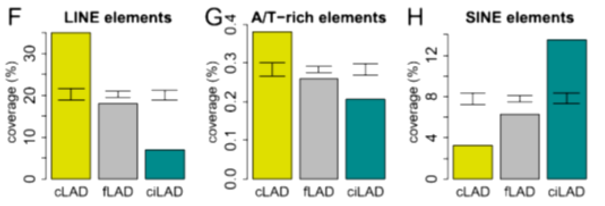

Percent coverage of constitutive LADs (cLADs), facultative LADs (fLADs), and constitutive inter-LADs (ciLADs) regions for LINE elements (F), simple A/T-rich elements (G), and SINE elements (H).

chromatin with the nuclear membrane and the overall genome architecture[36,38-40]. Deletion of both lamin A/C and LBR leads to dissociation of LAD-associated heterochromatin from the nuclear envelope and a reverse positioning of euchromatin and heterochromatin, which however, remain strongly segregated. These results indicate a secondary role for lamina scaffolding in chromatin compartmentalization.

3) Several studies reported biased distributions of L1 and B1 in several subnuclear structures, including pericentromere, nucleolus, and Barr body heterochromatin [41-44].

2. Imaging-based lines of evidence

4) Analysis of metaphase chromosome banding showed roughly inverse distributions of L1 and Alu elements in chromosomal regions with distinct biochemical properties (right)[45].

Cited from Kornberg and Rykowski, 1988. Cell
"Human genome organization: Alu, Lines, and the molecular structure of metaphase chromosome bands".

Cited from the original Figure 1 Cited from the original Figure 2

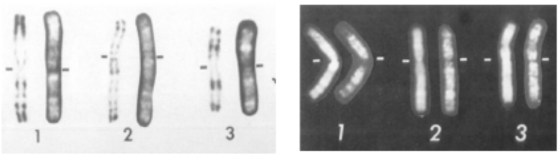

Distributions of Alu (left) and Ll (right) in human metaphase chromosomes 1 to 3 seen by in situ hybridization

Cited from Schermelleh L *et al.,* 2001. Chromosome Res.
"Two-color fluorescence labeling of early and mid-to-late replicating chromatin in living cells".

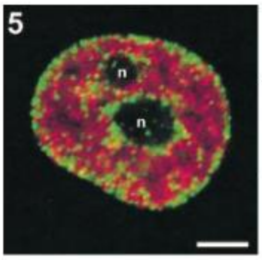

Typical distribution of early (Cy5-dUTP, red) and mid-to-late (Cy3-dUTP, green) replication chromatin.

5) Cremer group (2001) labeled DNA with thymidine analogues in living mammalian cells and suggested that DNA replication domains provide a high level of chromatin organization[46,47]. Mid-to-late replicating chromatin is mainly located at the nuclear periphery and around nucleoli. Early replication chromatin is distributed throughout the nuclear interior with exception of the nucleoli (left).

6) Cremer group (2005) performed Alu DNA FISH in human fibroblasts and lymphocytes[48]. They reported a concentration of Alu-rich chromatin in the nuclear interior, while Alu-poor chromatin forms a shell attached to the nuclear envelope (below). They proposed that chromatin domains, which are gene-poor, form a layer beneath the nuclear envelope, while gene-dense chromatin is enriched in the nuclear interior.

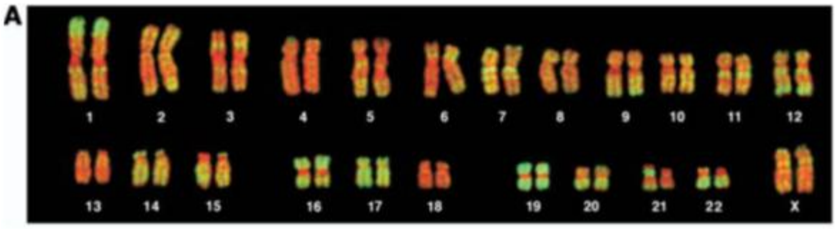

Cited from Bolzer and Cremer *et al.*, 2005. PLoS Biol.
"Three-dimensional maps of all chromosomes in human male fibroblast nuclei and prometaphase rosettes".

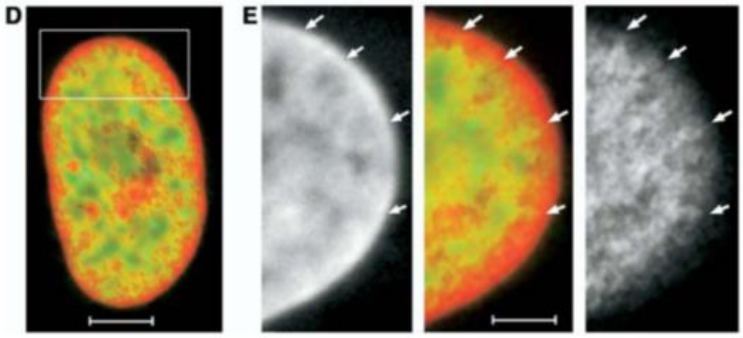

Figure 7. Localization of Alu Sequences in Nuclei of Fibroblasts and Lymphocytes.
(A) Karyotype from a female human lymphocyte (46, XX). Chromosomes were hybridized with a probe for Alu sequences (green) and counterstained with TOPRO-3 (red).
(D) Enlarged confocal mid-section through the human G0 fibroblast nucleus. Scale bar, 5 μm.
(E) Enlargement of the boxed sector in (D). Arrows indicate chromatin rich in Alu sequences expanding into the TOPRO-3-stained, Alu-poor nuclear rim. Scale bar, 2 μm. Alu staining, green; TOPRO-3 counterstaining, red.

7) Irina Solovei and Boris Joffe *et al*. (2009) reported the inverted nuclear architecture of nocturnal rod photoreceptor cells, where heterochromatin localizes in the nuclear center and euchromatin lines the nuclear border[37].

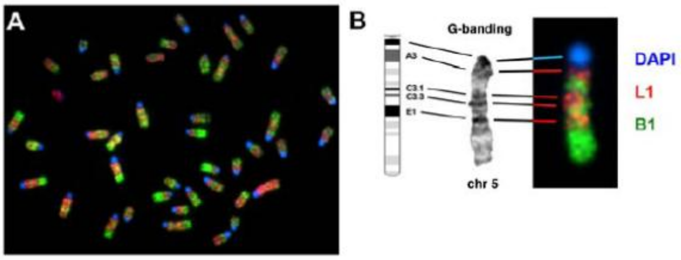

Figure S1. Banding pattern of mouse mitotic chromosomes after FISH with probes for marker chromatin sequences.
FISH with probes for L1 (red, L1-rich heterochromatin) and B1 (green, euchromatin). DNA was counterstained with DAPI (blue) that strongly staind pericentromeric heterochromatin.

In mammalian mitotic chromosomes, gene-poor and gene-rich regions alternate as G and R bands (Figures S1 and 4I; left).

Rod nuclei show a single microsatellite repeat (MSR) positive chromocenter that is surrounded by a thick shell of L1-rich chromatin and a thin outer shell of B1-rich euchromatin (Figure 1E1; below right).

By contrast, ganglion cells (Figure 1F1) and mouse embryonic fibroblasts (Figure 1G1) showed the conventional nuclear architecture: B1-rich gene-dense chromatin was found toward the interior of the nucleus, whereas L1-rich gene-poor chromatin adjoined the nuclear border. L1-rich chromatin also surrounded the chromocenters in ganglion cells but not in fibroblasts.

Cited from Solovei and Joffe *et al*., 2009. Cell
"Nuclear Architecture of Rod Photoreceptor Cells Adapts to Vision in Mammalian Evolution"

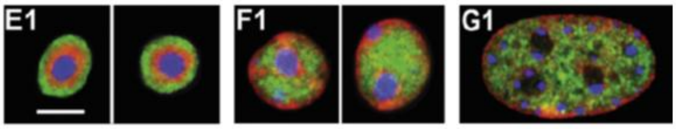

Figure 1. Nuclear Architecture of Mouse Rods in Comparison to Other Cells.
(E1–G1) Radial distribution of constitutive heterochromatin (MSR, blue), L1-rich heterochromatin (L1, red), and euchromatin (B1, green) in the nuclei of rods (E), ganglion cells (F) and fibroblasts (G). Mid optical sections of nuclei.

They also revealed that the inverted pattern is progressively established during differentiation of rod nuclei (below). At P0, rod nuclei had a conventional architecture: the L1 signal at the nuclear periphery and around the chromocenters, and the B1 signal at a more internal position. By P28, the conventional arrangement was transformed into the inverted one. Analysis of the course of this remodeling led them to conclude that it is the nuclear positions and orientation of chromosome territories that are different between the two inverted and conventional patterns. ***However, the features that predetermine the nearly universal prevalence of the conventional nuclear architecture still remain unanswered in their study***.

Cited from Solovei and Joffe *et al*., 2009. Cell
"Nuclear Architecture of Rod Photoreceptor Cells Adapts to Vision in Mammalian Evolution"

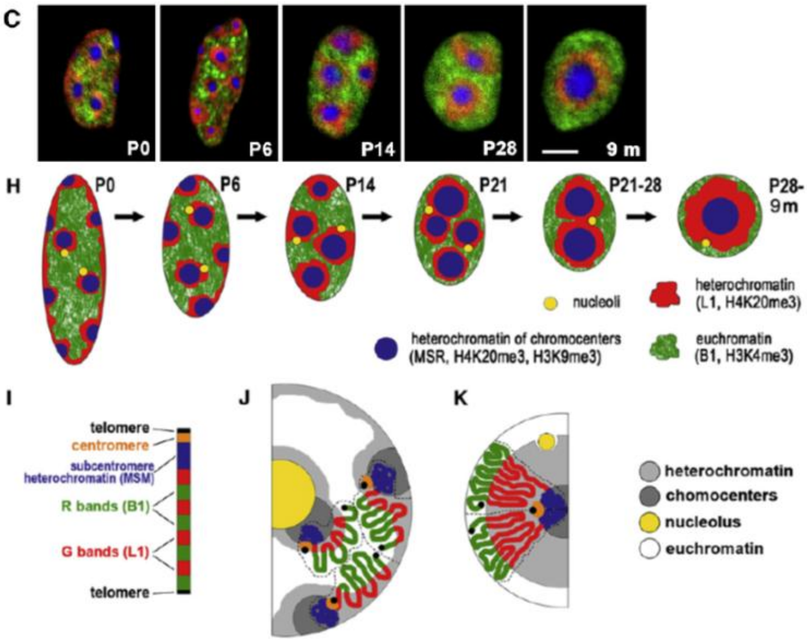

Figure 4. Inversion of the Nuclear Architecture during Terminal Differentiation of Mouse Rods
The organization of the rod nuclei in postnatal development (P0–P28) and in a 9-month-old mouse. (C) FISH with probes for L1 (red), B1 (green), and MSR (chromocenters, blue). (H) Scheme of the remodeling of the nuclear architecture. (I–K) Scheme of the distribution of chromosome subregions (I) in interphase nuclei with the conventional (J) and inverted (K) architecture.

8) Jachowicz and Torres-Padilla *et al*. (2017) reported a role of LINE-1 (L1) in regulating global chromatin accessibility at the beginning of development[49]. LINE-1 DNA FISH detected strong signals at the peripheries of the nucleus and nucleolus (Supplementary Figure 1c; below), the pattern mirroring what we have reported in our paper. However, engineered TALEs that target various regions of LINE-1 DNA showed a *widespread* distribution pattern. They stated in the paper: “We confirmed the nuclear localization of each of these TALEs in mouse ES cells by immunostaining, which showed ***a widespread nuclear distribution similar to that of endogenous LINE-1***, as determined by DNA FISH (Supplementary Fig. 1c)”.

Cited from Jachowicz and Torres-Padilla *et al*., 2017. Nature Genetics
“LINE-1 activation after fertilization regulates global chromatin accessibility in the early embryo”

Supplementary Figure 1C

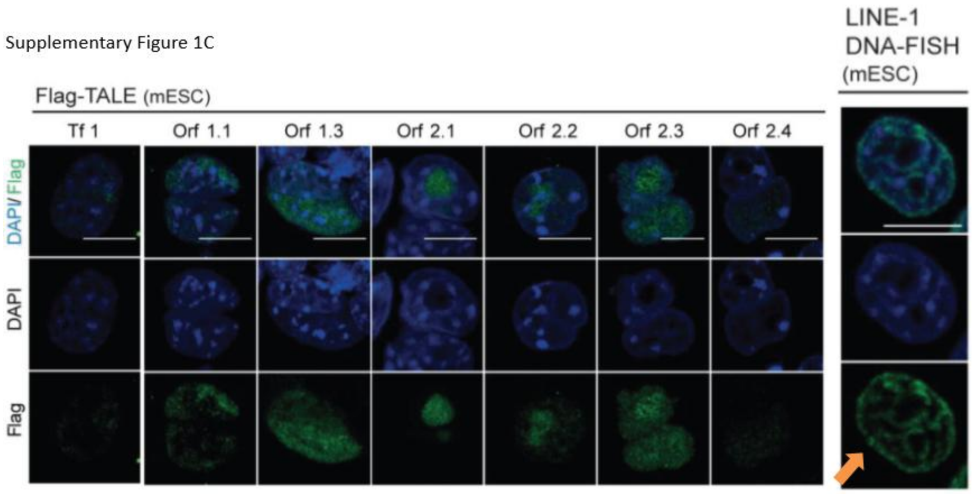

Supplementary Figure 1c. Representative images of expression and localization of Flag-TALE-L1 constructs in mES cells (left) compared to endogenous LINE-1 by DNA-FISH (right).
N=2 (independent transfection experiments). Seven different TALE-L1 constructs described in Fig. S1a were detected using an anti-FLAG antibody (green). Maximal projection of Z-confocal sections are shown. Scale bars, 10 µm.

9) Lenain and Peeper *et al*. (2017) mainly utilized DamID to investigate LAD re-organization during cellular senescence by using the technology[36]. They reported that SINE-enriched chromatin moves from the nuclear interior toward the periphery in oncogene-induced senescence (OIS) cells by DNA FISH (right).

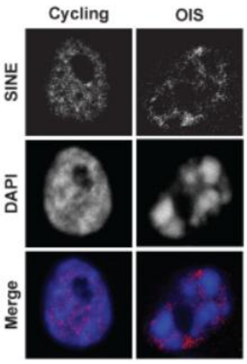

Cited from Lenain, Steensel, and Peeper *et al*., 2017. Genome Research. “Massive reshaping of genome-nuclear lamina interactions during oncogene-induced senescence”.

Figure 3G. Single z-section example of confocal images of cycling and OIS cells stained with a SINE probe.

Cited from Vazquez and Serrano *et al*., 2019. Nucl. Acids Res.
“SIRT7 mediates L1 elements transcriptional repression and their association with the nuclear lamina”.

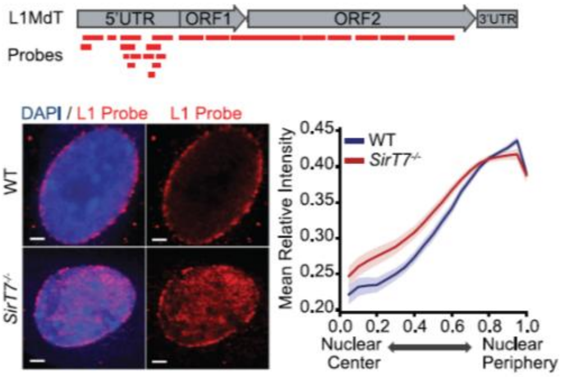

Figure 2F. FISH analysis of the nuclear distribution of L1MdT family.
(Top) L1MdT consensus sequence annotated with location of FISH probes. (Left) Representative images (scale bar=2µm). (Right) L1MdT probe intensity plotted against nuclear radial position.

10) Vazquez and Serrano *et al*. (2019) reported disrupted nuclear distribution of L1MdT DNA in SirT7−/− cells and suggested a role of SIRT7 in regulating the anchoring of L1 elements to the nuclear periphery[50]. In this study, they performed L1 DNA FISH and detected L1 signals at the

nuclear periphery, and identified a functional interplay between SIRT7 and Lamin A/C in L1 repression (left).

11) Falk, Solovei and Mirny *et al*. (2019) employed an *in silico* simulation method and proposed that heterochromatin drives compartmentalization of inverted and conventional nuclei[51]. Using polymer simulations to reconcile microscopy and Hi-C data, they have predicted that: (i) interactions between heterochromatic regions lead to phase separation of chromatin and are crucial for establishing the segregated compartments of euchromatin and heterochromatin in both conventional and inverted nuclei; (ii) euchromatic interactions are dispensable for compartmentalization; (iii) lamina-heterochromatin interactions are dispensable for the segregation of euchromatin and heterochromatin, but are necessary to build the conventional nuclear architecture from segregated active and inactive phases. As shown in their Extended Data Fig. 10b, mutant rod cells re-expressing lamin A/C acquire partially de-inverted morphologies that are remarkably similar to simulations.

Cited from Falk, Solovei and Mirny *et al*., 2019. Nature.
"Heterochromatin drives compartmentalization of inverted and conventional nuclei".

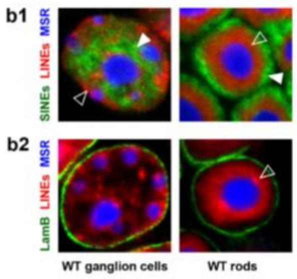

Extended Data Fig. 10b (partial): Conventional nuclei of ganglion cells (left) and inverted rod nuclei (right). FISH with probes for major satellite repeats (blue), LINE-rich heterochromatin (red) and SINE-rich euchromatin (green).
(This legend is modified based on the original description in Falk *et al*., 2019.)

They utilized LINEs and SINEs to label heterochromatin and euchromatin, respectively (above). Unfortunately, they did not link these repeats to chromatin organization. As they stated in the discussion, "Although we narrow the search for key molecular determinants of compartmentalization to heterochromatin-associated molecules, ***making predictions for perturbations to particular molecular determinants remains a limitation of our current study***. Candidates for mediator of heterochormatin-heterochromatin interactions include affinity between homotypic repetitive elements or modified histones, and heterochromatin-associated proteins (for example, HP1)."

3. HiC-based evidence

12) Stevens and Laue *et al.* (2017) performed single-cell based Hi-C analysis[9]. They reported that structures of individual TADs and loops vary substantially from cell to cell, whereas the 3D genome architecture, particularly A and B compartments, lamina-associated domains (LADs) and active enhancers and promoters, are conserved in all cells. Simulation of single-cell Hi-C data predicted that the euchromatic A

Cited from Stevens and Laue *et al*., 2017. Nature.
"3D structures of individual mammalian genomes studied by Single-cell Hi-C".

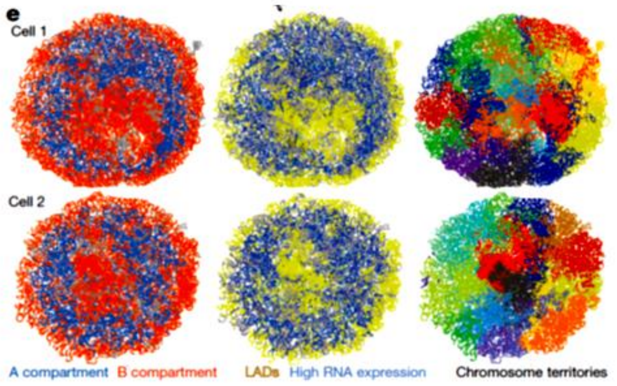

Figure 2e, Cross-sections through five superimposed 3D structures from two different cells, colored according to: whether the sequence is in the A or B compartment (left); whether the sequence is part of a cLAD (yellow) or contains highly expressed genes (blue) (centre); and chromosome identity (right).

compartment adopts a central position, whereas the heterochromatic B compartment moves towards the periphery of nucleus and nucleolus. However, direct visualization of the A/B compartments using common molecular marks remains to be tested experimentally.

4. Evidence linking DNA sequences to chromatin organization

13) Bailey and Eichler *et al.* (2000) reported enriched distribution of L1 elements in the X chromosome than autosomes and suggested a link of L1 to X-chromosome inactivation (XCI)[43].

14) Chow and Heard *et al.* (2010) reported evidence for L1 elements in regulating heterochromatin formation during X chromosome inactivation at different levels[42]. Silent L1s participate in assembly of a heterochromatic nuclear compartment induced by the lncRNA *Xist*, while transient transcription of certain young L1s facilitates local propagation of the silencing into regions that would be otherwise prone to escape[42]. In addition, to investigate LINE distribution and silencing efficiency of XCI in an authosomal context, they characterized two cell lines carrying inducible *Xist* cDNA transgene that is integrated on chromosome 17 or 11. They found that proximity to *Xist* transgene is not sufficient for silencing and the presence and density of L1 sequences may play a major role in ensuring efficient XCI.

15) Cournac and Mozziconacci *et al.* (2016) analyzed the folding of the human, mouse and *Drosophila* genomes based on published Hi-C data[52]. They found that the 3D folding of metazoan genomes correlates with the association of similar repetitive elements, notably many elements of the SINE family in homologous genome regions in human and mouse. In addition, they compared the folding of the *Drosophila* genome with that of human and mouse genomes. Although flies do not have SINE elements, still, significant enrichments in contacts between several classes of repetitive elements were observed, including Gypsy and Invaders subfamilies from the LTF family and the ProtoP elements from the DNA transposons family. Thus, a subset of repetitive elements always exhibits high enrichments in inter-chromosomal contacts, despite important divergences in the evolutionary history of repeats in mouse, human and fly genomes. They suggested a contribution of specific repetitive elements in the regulation of the folding of many metazoan genomes over evolutionary times. While tantalizing, the role of L1 repeats was not recognized in this paper.

16) Werken, Solovei, and Joffe *et al.* (2017) analyzed the spatial intranuclear arrangement of a human artificial chromosome (HAC) in comparison to an orthologous region of native mouse chromosome by DNA FISH and 4C-seq technologies[53]. They showed that chromatin segments (0.6 to 3 Mb) cluster with segments of the same chromatin class, including SINE-rich euchromatin, LINE/LTR-rich heterochromatin, and satellite DNA-containing constitutive heterochromatin, suggesting an autonomous property. They proposed that building of a functional nucleus is largely a self-organizing process based on mutual recognition of chromosome segments belonging to the major chromatin classes.

References:

1 Heintz, E. Das heterochromatin der moose. *Jahresber Wiss Botanik* **69**, 762-818 (1928).

2 Dixon, J. R. *et al.* Topological domains in mammalian genomes identified by analysis of chromatin interactions. *Nature* **485**, 376-380, doi:10.1038/nature11082 (2012).

3 Dixon, J. R. *et al.* Chromatin architecture reorganization during stem cell differentiation. *Nature* **518**,

331-336, doi:10.1038/nature14222 (2015).

4 Schmitt, A. D. *et al.* A Compendium of Chromatin Contact Maps Reveals Spatially Active Regions in the Human Genome. *Cell Rep* **17**, 2042-2059, doi:10.1016/j.celrep.2016.10.061 (2016).

5 Stadhouders, R. *et al.* Transcription factors orchestrate dynamic interplay between genome topology and gene regulation during cell reprogramming. *Nature Genetics* **50**, 238-249, doi:10.1038/s41588-017-0030-7 (2018).

6 Rao, S. S. *et al.* A 3D map of the human genome at kilobase resolution reveals principles of chromatin looping. *Cell* **159**, 1665-1680, doi:10.1016/j.cell.2014.11.021 (2014).

7 Lieberman-Aiden, E. *et al.* Comprehensive mapping of long-range interactions reveals folding principles of the human genome. *Science* **326**, 289-293, doi:10.1126/science.1181369 (2009).

8 Rennie, S., Dalby, M., van Duin, L. & Andersson, R. Transcriptional decomposition reveals active chromatin architectures and cell specific regulatory interactions. *Nat Commun* **9**, 487, doi:10.1038/s41467-017-02798-1 (2018).

9 Stevens, T. J. *et al.* 3D structures of individual mammalian genomes studied by single-cell Hi-C. *Nature* **544**, 59-64, doi:10.1038/nature21429 (2017).

10 Lu, J. Y. *et al.* Genomic Repeats Categorize Genes with Distinct Functions for Orchestrated Regulation. *Cell Rep* **30**, 3296-3311 e3295, doi:10.1016/j.celrep.2020.02.048 (2020).

11 Sexton, T. & Cavalli, G. The role of chromosome domains in shaping the functional genome. *Cell* **160**, 1049-1059, doi:10.1016/j.cell.2015.02.040 (2015).

12 Dixon, J. R., Gorkin, D. U. & Ren, B. Chromatin Domains: The Unit of Chromosome Organization. *Mol Cell* **62**, 668-680, doi:10.1016/j.molcel.2016.05.018 (2016).

13 Nagano, T. *et al.* Single-cell Hi-C reveals cell-to-cell variability in chromosome structure. *Nature* **502**,

59-64, doi:10.1038/nature12593 (2013).

14 Boettiger, A. N. *et al.* Super-resolution imaging reveals distinct chromatin folding for different epigenetic states. *Nature* **529**, 418-422, doi:10.1038/nature16496 (2016).

15 Wang, S. *et al.* Spatial organization of chromatin domains and compartments in single chromosomes. *Science* **353**, 598-602, doi:10.1126/science.aaf8084 (2016).

16 Bintu, B. *et al.* Super-resolution chromatin tracing reveals domains and cooperative interactions in single cells. *Science* **362**, doi:10.1126/science.aau1783 (2018).

17 Tan, L., Xing, D., Chang, C. H., Li, H. & Xie, X. S. Three-dimensional genome structures of single diploid human cells. *Science* **361**, 924-928, doi:10.1126/science.aat5641 (2018).

18 Ramskold, D., Wang, E. T., Burge, C. B. & Sandberg, R. An abundance of ubiquitously expressed genes revealed by tissue transcriptome sequence data. *PLoS Comput Biol* **5**, e1000598, doi:10.1371/journal.pcbi.1000598 (2009).

19 Isoda, T. *et al.* Non-coding Transcription Instructs Chromatin Folding and Compartmentalization to Dictate Enhancer-Promoter Communication and T Cell Fate. *Cell* **171**, 103-119 e118, doi:10.1016/j.cell.2017.09.001 (2017).

20 Vastenhouw, N. L. & Schier, A. F. Bivalent histone modifications in early embryogenesis. *Curr Opin Cell Biol* **24**, 374-386, doi:10.1016/j.ceb.2012.03.009 (2012).

21 Rivera, C. M. & Ren, B. Mapping human epigenomes. *Cell* **155**, 39-55, doi:10.1016/j.cell.2013.09.011 (2013).

22 Ray, J. *et al.* Chromatin conformation remains stable upon extensive transcriptional changes driven by heat shock. *Proc Natl Acad Sci U S A* **116**, 19431-19439, doi:10.1073/pnas.1901244116 (2019).

23 Therizols, P. *et al.* Chromatin decondensation is sufficient to alter nuclear organization in embryonic

stem cells. *Science* **346**, 1238-1242, doi:10.1126/science.1259587 (2014).

24 Tumbar, T., Sudlow, G. & Belmont, A. S. Large-scale chromatin unfolding and remodeling induced by VP16 acidic activation domain. *J Cell Biol* **145**, 1341-1354, doi:10.1083/jcb.145.7.1341 (1999).

25 Rowley, M. J. *et al.* Evolutionarily Conserved Principles Predict 3D Chromatin Organization. *Mol Cell* **67**, 837-852 e837, doi:10.1016/j.molcel.2017.07.022 (2017).

26 Hug, C. B., Grimaldi, A. G., Kruse, K. & Vaquerizas, J. M. Chromatin Architecture Emerges during Zygotic Genome Activation Independent of Transcription. *Cell* **169**, 216-228 e219, doi:10.1016/j.cell.2017.03.024 (2017).

27 Tan, L., Xing, D., Daley, N. & Xie, X. S. Three-dimensional genome structures of single sensory neurons in mouse visual and olfactory systems. *Nat Struct Mol Biol* **26**, 297-307, doi:10.1038/s41594-019-0205-2 (2019).

28 Lander, E. S. *et al.* Initial sequencing and analysis of the human genome. *Nature* **409**, 860-921, doi:10.1038/35057062 (2001).

29 Mouse Genome Sequencing, C. *et al.* Initial sequencing and comparative analysis of the mouse genome. *Nature* **420**, 520-562, doi:10.1038/nature01262 (2002).

30 Bailey, J. A., Liu, G. & Eichler, E. E. An Alu transposition model for the origin and expansion of human segmental duplications. *Am J Hum Genet* **73**, 823-834, doi:10.1086/378594 (2003).

31 Price, A. L., Eskin, E. & Pevzner, P. A. Whole-genome analysis of Alu repeat elements reveals complex evolutionary history. *Genome Res* **14**, 2245-2252, doi:10.1101/gr.2693004 (2004).

32 Jurka, J., Kohany, O., Pavlicek, A., Kapitonov, V. V. & Jurka, M. V. Duplication, coclustering, and selection of human Alu retrotransposons. *Proc Natl Acad Sci U S A* **101**, 1268-1272, doi:10.1073/pnas.0308084100 (2004).

33 Meuleman, W. *et al.* Constitutive nuclear lamina-genome interactions are highly conserved and associated with A/T-rich sequence. *Genome Res* **23**, 270-280, doi:10.1101/gr.141028.112 (2013).

34 Guelen, L. *et al.* Domain organization of human chromosomes revealed by mapping of nuclear lamina interactions. *Nature* **453**, 948-951, doi:10.1038/nature06947 (2008).

35 Pickersgill, H. *et al.* Characterization of the Drosophila melanogaster genome at the nuclear lamina. *Nature Genetics* **38**, 1005-1014, doi:10.1038/ng1852 (2006).

36 Lenain, C. *et al.* Massive reshaping of genome-nuclear lamina interactions during oncogene-induced senescence. *Genome Res* **27**, 1634-1644, doi:10.1101/gr.225763.117 (2017).

37 Solovei, I. *et al.* Nuclear architecture of rod photoreceptor cells adapts to vision in mammalian evolution. *Cell* **137**, 356-368, doi:10.1016/j.cell.2009.01.052 (2009).

38 Amendola, M. & van Steensel, B. Nuclear lamins are not required for lamina-associated domain organization in mouse embryonic stem cells. *EMBO Rep* **16**, 610-617, doi:10.15252/embr.201439789 (2015).

39 Zheng, X. *et al.* Lamins Organize the Global Three-Dimensional Genome from the Nuclear Periphery. *Mol Cell* **71**, 802-815 e807, doi:10.1016/j.molcel.2018.05.017 (2018).

40 Chang, L. *et al.* Chromatin-lamin B1 interaction promotes genomic compartmentalization and constrains chromatin dynamics. *bioRxiv*, 601849, doi:10.1101/601849 (2019).

41 Wijchers, P. J. *et al.* Characterization and dynamics of pericentromere-associated domains in mice. *Genome Res* **25**, 958-969, doi:10.1101/gr.186643.114 (2015).

42 Chow, J. C. *et al.* LINE-1 activity in facultative heterochromatin formation during X chromosome inactivation. *Cell* **141**, 956-969, doi:10.1016/j.cell.2010.04.042 (2010).

43 Bailey, J. A., Carrel, L., Chakravarti, A. & Eichler, E. E. Molecular evidence for a relationship between

LINE-1 elements and X chromosome inactivation: the Lyon repeat hypothesis. *Proc Natl Acad Sci U S A* **97**, 6634-6639 (2000).

44 Nemeth, A. *et al.* Initial Genomics of the Human Nucleolus. *Plos Genet* **6**, doi:ARTN e1000889 10.1371/journal.pgen.1000889 (2010).

45 Korenberg, J. R. & Rykowski, M. C. Human genome organization: Alu, lines, and the molecular structure of metaphase chromosome bands. *Cell* **53**, 391-400 (1988).

46 Schermelleh, L., Solovei, I., Zink, D. & Cremer, T. Two-color fluorescence labeling of early and mid-to-late replicating chromatin in living cells. *Chromosome Res* **9**, 77-80, doi:10.1023/a:1026799818566 (2001).

47 Cremer, T. & Cremer, C. Chromosome territories, nuclear architecture and gene regulation in mammalian cells. *Nat Rev Genet* **2**, 292-301, doi:10.1038/35066075 (2001).

48 Bolzer, A. *et al.* Three-dimensional maps of all chromosomes in human male fibroblast nuclei and prometaphase rosettes. *PLoS Biol* **3**, e157, doi:10.1371/journal.pbio.0030157 (2005).

49 Jachowicz, J. W. *et al.* LINE-1 activation after fertilization regulates global chromatin accessibility in the early mouse embryo. *Nature Genetics* **49**, 1502-1510, doi:10.1038/ng.3945 (2017).

50 Vazquez, B. N. *et al.* SIRT7 mediates L1 elements transcriptional repression and their association with the nuclear lamina. *Nucleic Acids Res* **47**, 7870-7885, doi:10.1093/nar/gkz519 (2019).

51 Falk, M. *et al.* Heterochromatin drives compartmentalization of inverted and conventional nuclei. *Nature* **570**, 395-399, doi:10.1038/s41586-019-1275-3 (2019).

52 Cournac, A., Koszul, R. & Mozziconacci, J. The 3D folding of metazoan genomes correlates with the association of similar repetitive elements. *Nucleic Acids Res* **44**, 245-255, doi:10.1093/nar/gkv1292 (2016).

53 van de Werken, H. J. G. *et al.* Small chromosomal regions position themselves autonomously according to their chromatin class. *Genome Res* **27**, 922-933, doi:10.1101/gr.213751.116 (2017).
