# Supplementary material for: Homotypic clustering of L1 and B1/Alu repeats compartmentalizes the 3D genome
Source: Cell Res. 2021 Jan 29;31(6):613–30. doi: 10.1038/s41422-020-00466-6 (PMC8169921; doi:10.1038/s41422-020-00466-6)
Supplement: Supplementary file 20 — Supplementary information, Data S1 [file 41422_2020_466_MOESM20_ESM.pdf]

Supplementary Information, Data S1.

Materials and Methods

Density and correlation analysis of genomic repeats

The reference catalog of repetitive elements was built from RepeatMasker annotations [1]. The raw annotation was downloaded from UCSC Table Browser (http://genome.ucsc.edu/) in the "Variation and Repeats" section. We removed repetitive elements marked by "?" at the end of "Family" or "Class" name (for example, Alu?) which represent unclear classification. Finally, 96.1% of whole annotated repetitive element were retained for the following analysis.

The mouse (human) genome was first segmented into bins with various sizes ranging from 5 kb, 10 kb, 20 kb to 50 kb. We calculated the percentage of individual repeat sub-families in each bin. The pattern of L1 and B1 distributions in repeat density was similar regardless of bin size. So we chose to use the 10-kb bin in all repeat analyses, including figure panels showing tracks of L1 and B1 density and distribution, unless otherwise indicated.

In Fig. 1b, each compartment was first divided into 10 bins with equal size. We calculated the percentage of L1 and B1/Alu repeat sub-family in each bin (% of each bin made up by individual repeat sub-families). As the genomic contents of L1 and B1/Alu repeats are different, we calculated the relative contents of repeats in the adjacent A and B compartments (20 bins) with Z-Score formula: [Xi-average(X)] / standard deviation(X); Xi represents the repeat content in the i_{th} bin.

In Extended Data Fig. 1a, we calculated the percentage of each repeat sub-family in each bin (B1, B2, B4, Satellite, L2, MIR, Low_complexity, Simple_repeat and L1) and performed Pearson correlation analysis. In Extended Data Fig. 1e, the corresponding human homologous regions in each bin were identified with the liftover tool from the UCSC Browser[2]. The repeat density and average eigenvalues of the Hi-C contact matrix were calculated for each bin before Pearson correlation analysis was performed. For both analyses, 1-Pearson correlation coefficient was used as the distance for the final unsupervised hierarchical clustering.

RNA-seq and ChIP-seq analysis

Alignments of RNA-Seq data to mouse genome assembly mm10 were performed using Tophat v2.0.10[3]. Fragments Per Kilobase of exon model per Million mapped reads (FPKM) were calculated by Cufflink 2.1.1 to represent expression levels of transcripts. Gencode v19 was used as the human gene annotation[4].The ChIP-seq data were analyzed as previously described. All ChIP -seq reads were aligned to the mouse genome assembly mm9 using Bowtie2 (version 2.2.2)[5]. All the ChIP-seq peaks were called by MACS with default parameters[6].

Hi-C and compartmental analysis

Paired-end raw reads of Hi-C library data were processed with HiCPro (version 2.7.7) as described [7]. Briefly, sequencing reads were first independently mapped to the mouse reference genome (mm9) using the bowtie2 algorithm and the option '-very-sensitive 'BOWTIE2_GLOBAL_OPTIONS = --very-sensitive -L 30 --score-min L,-0.6,-0.2 --end-to-end –reorder; BOWTIE2_LOCAL_OPTIONS = --very-sensitive -L 20 --score-min L,-0.6,-0.2 --end-to-end –reorder'. Pairs of aligned reads were then compared to MboI restriction enzyme fragments in order to filter out read pairs from uncut DNA, self-circle ligation and PCR artefacts. Only the valid read pairs involving two different restriction fragments were used to build the contact matrix. Valid read pairs were then binned at a specific resolution by dividing the genome into bins of equal size. We chose a 100-kb or 500-kb bin size for examination of global interaction patterns of the genome, and a 40-kb bin size to show local interactions and to perform TAD calling. The binned interaction matrices were then normalized with the iterative correction method[8] to correct for biases including GC content, mappability and effective fragment length in Hi-C data.

All the Hi-C interaction frequency heatmaps of whole chromosomes and the zoomed-in views were generated using HiCPlotter (Version 0.7.3) and a Hi-C data visualization tool[9]. All the Hi-C

data were normalized contact matrices. The 'triangle' interaction heatmaps were generated with 3D Genome Browser tools[10]. To generate the correlation heatmap, the total correlation matrices for each stage were generated as previously described[11].

A and B compartments were identified as described previously[11]. The normalized contact matrix with a bin size of 500 kb or 40 kb were used. Principal component analysis (PCA) was performed and the first principal component of the normalized matrix coupled with protein-coding gene density were used to identify A/B compartments. Based on mESC Hi-C data[12], 730 compartments were identified with PCA. As the X chromosome can randomly be inactive, only the 696 autosomal compartments, which had a median size of 1.9 Mb and covered 85% of the whole mouse genome, were kept for further analysis. For the analysis of Hi-C data in 21 human cell lines and tissues, PC1 values of Hi-C contact matrix were obtained from Table S2 of the previous report[13]. We first removed those regions with unavailable PC1 score and calculated the percentage of genomic regions showing consistent compartment assignments across different cell lines and tissues, which were labeled as "observed". The expected distribution of compartments across cell lines and tissues are conducted as preciously described[13]. In brief, the compartment for each region was randomly assigned with preserving the total number of A or B compartments on each chromosome. We then calculated the percentage of genomic regions showing consistent compartment assignments after the random compartmental labeling, which reflect the conservation level of compartment "by chance".

Compartmentalization strength was identified as previously described[14-16]. In brief, we calculated observed/expected Hi-C matrices for 100 kb binned data, correcting for average distance decays observed in the P(s) plots. We then arranged observed/expected matrix bins according to their Eigenvector1 values. We aggregated the ordered matrices for each chromosome within a dataset and then divided the aggregate matrix into 100 bins and plotted, yielding a saddle plot (cooltools). Strength of compartmentalization was defined as the ratio of (A–A + B–B)/(A–B + B–

A) interactions. The values used for this ratio were determined by calculating the mean value of the 20 bins (20%) in each corner of the saddle plot.

De novo compartment calling based on L1 and B1 DNA sequences

The mouse genome was first segmented into 100-kb bins. By intersecting with L1 and B1 repeats, we calculated the percentage of L1 and B1 repeats in each bin. The densities of L1 and B1 repeats were normalized with their genome background (19% for L1 and 3% for B1). We then calculated the ratio of normalized B1 to L1 densities and further transferred the ratio to log2 scale [log2(B1/L1)]. Adjacent regions with log2(B1/L1) larger or smaller than zero were merged, respectively. The merged regions with size > 500-kb were kept (85% left) and assigned as B1-rich (540) or L1-rich (648) compartments with a median size of 1.1 Mb to 1.3 Mb (Supplementary information, Table S1). The numbers and sizes of these B1- and L1-rich compartments called *de novo* are comparable to A and B compartments annotated by Hi-C in mESCs (366 and 364, respectively, with a median size of 1.9 Mb). In mESCs, ~85% of the genome has PC1 values of Hi-C interaction matrix, where 46% are A compartments (PC1 >0) and 54% are B compartments (PC1 <0). About 82% B1-rich active compartments are assigned as the A compartment by Hi-C data and 77% L1-rich inactive compartment are assigned as the B compartment by Hi-C data.

Segregation index

Imaging: Pearson's correlation coefficients between B1 and L1 labeled channels within nuclei were quantified by Volocity[17]. They were normalized to background signals obtained from five randomly chosen areas outside the nucleus. To quantify the degree of nuclear segregation of L1 and B1 DNA, we defined a FISH-based segregation index as the negative value of Pearson's correlation coefficient of L1 and B1 DNA signals in the nucleus. Thus, a higher segregation index means a smaller Pearson's correlation coefficient of L1 and B1 signals, indicating poor co-

localization and better segregation of L1 and B1.

Hi-C: We defined a Hi-C-based segregation index by calculating the ratio of homotypic versus heterotypic interaction frequencies between L1 and B1/Alu subfamilies. Heterotypic interaction frequency represents the interaction between different classes of compartments (L1-rich compartments and B1-rich compartments). Homotypic interaction frequencies are interactions between the same classes of compartments. Therefore, the Hi-C segregation index is defined as (L1-L1 + B1-B1) / L1-B1. A higher segregation index represents more homotypic but less heterotypic interaction frequency.

DNA, Oligopaint and immuno-FISH

DNA FISH of L1 and B1 in mESCs was performed as previously described[18]. mESCs growing on 35 mm glass-bottom dishes were fixed with 4% paraformaldehyde (PFA) diluted by phosphate-buffered saline (PBS) for 10 min at room temperature (RT), and then washed with PBS for 2 min, followed by permeabilization with 0.5% Triton X-100 in PBS. After three washes with PBS, mESCs were treated with 0.1 M HCl for 5 minutes and then incubated with 0.1 mg/mL RNase A diluted in PBS for 45 minutes at 37 ℃. Prior to prehybridization, cells were washed three times with 2 ×saline-sodium citrate (SSC) buffer. Prehybridization was performed by incubating cells in 50% formamide diluted in 2 ×SSCT (2 ×SSC + 0.1% Tween-20) for 5 minutes at RT, then for 20 minutes at 47 ℃. Samples were denatured for 2.5 minutes at 78 ℃ on the top of a water-immersed heat block. For hybridization, 200 µl hybridization buffer containing 2 ×SSC, 50% formamide, 20% dextran sulfate, and 0.5 µM of each of the B1 and L1 probes was applied to cover the glass bottom and then samples were incubated in a humidified chamber at 37 ℃ for about 16 hours. Finally, mESCs were washed twice with 2 ×SSCT for 15 minutes at 50 ℃, and then washed twice with 2x SSCT for 1 hour at RT. The labeled mESCs were rinsed briefly with 2 ×SSC and mounted with ProLong® Diamond Antifade Mountant with DAPI (P36962, Thermo Fisher Scientific).

For embryos, DNA FISH was performed as above with several optimizations. 0.1% Tween-20 was added to all buffers. Embryos were fixed with 1% PFA (instead of 4% PFA) at 4 ℃ overnight. The denaturation time for embryos was 10 minutes (compared to 2.5 minutes for mESCs). Embryos were additionally incubated in hybridization buffer at 86 ℃ for 3 minutes before hybridization overnight. After washing at 50 ℃, embryos were washed with 2×SSCT at 4 ℃ overnight. Probes targeting to consensus sequence of L1 and B1 (Fig. 2a) were used in both mESCs and embryos (Supplementary information, Table S2)

For Oligopaint FISH analysis of non-repetitive sequences in A/B compartments, probe sets were designed using standard procedures to target the genomic regions defined in Supplementary information, Table S3. We chose four regions in ~100-kb length, including B1-rich regions *F, H* and *R,* and L1-rich region *e,* each of which are targeted by 500 DNA probes for FISH analysis. We chose two large L1-rich regions *g* and *q* (~1 Mb), each of which are targeted by 4,500 DNA probes for FISH.

For the primary probe pool, we purchased the Oligoarray pool (Synbio Technologies), and prepared FISH probes via limited cycle PCR, *in vitro* transcription, and reverse transcription as described previously[18]. The primary probes were freshly mixed with corresponding secondary probes in hybridization buffer at 1 μM final concentration for each secondary probe. The hybridization buffer with the primary and secondary probes was heated to 86 ℃ for 3 min, and then placed on ice immediately. Cell samples were prepared for DNA FISH as described above for mESCs, except that the cells were incubated in hybridization buffer at 86 ℃ for 3 minutes before hybridization overnight.

Immuno-FISH in different cell types was performed as described[19]**.** DNA FISH was performed with B1 and L1 probes following the DNA FISH protocol for mESCs described above. After cells were washed three times in 2x SSCT, immunofluorescence labeling of proteins was performed. Samples were permeabilized with 0.5% Triton-X in PBS at room temperature for 5 min, and then

blocked with blocking buffer containing 5% BSA and 0.1% Triton-X in 1x PBS at room temperature for 30 min. The samples were incubated with primary antibodies in a humidified chamber at 4 ℃ overnight. Antibodies were diluted in blocking buffer. The primary antibody was removed by washing the samples three times in 1x PBS at room temperature for 5 minutes each time. The cells were incubated with secondary antibody solution at room temperature for 1 hour, then washed three times with 1x PBS for 5 minutes each time. Finally, samples were mounted with ProLong® Diamond Antifade Mountant with DAPI. For NCL immuno-FISH, rabbit polyclonal anti-NCL (Abcam; ab22758; 1:1000) and Alexa Fluor 488 donkey anti-rabbit IgG (H+L) (Thermo Fisher Scientific; A21206; 1:1000) were used as the primary and secondary antibodies, respectively.

RNA FISH

RNA FISH of mESCs and embryos was performed as described before[20]. Cells were fixed in 4% PFA for 20 minutes at RT and then rinsed twice with PBS. The cell permeabilization step was done in 70% ethanol at 4 ℃ overnight. After washing with PBS, cells were incubated in 15% formamide freshly prepared in 1x SSC buffer for 15 minutes at RT, followed by hybridization at 37°C using probes targeting B1 and L1 in 200 μl hybridization buffer. The samples were washed twice with 15% formamide in 1×SSC for 30 minutes at 37 ℃, and then washed again at RT. Finally, cells were mounted after rinsing with PBS. All the reagents and containers were RNase-free. For embryos, 0.1% Tween-20 was added to each buffer, and fixation was carried out with 1% PFA at 4 ℃ overnight.

Optical setup, image acquisition and quantification

All imaging was performed with an UltraVIEW VoX spinning disc microscope (PerkinElmer) except for the 3D imaging of DNA FISH and immuno-FISH samples. For RNA FISH samples, z-stacks covering the whole nucleus with a step size of 400 nm were taken for each channel and

imaging conditions were kept consistent among different samples in one experiment. To reduce the effect of photobleaching on quantification, the channel that needs to be quantified was imaged first. In DNA FISH image collection, one layer of both channels was acquired at 10 time points. DNA FISH images were prepared for presentation by averaging 10 images of each channel to increase the signal-to-noise ratio. For 3D imaging of DNA FISH and immuno-FISH samples, images of nuclei were collected with a Dragonfly spinning disc microscope (Andor) with a 200-400 nm step size for each channel.

The 3D DNA FISH images of genomic regions in A/B compartments were analyzed by Imaris version 8.4.1 (Bitplane Inc, software available at http://www.bitplane.com/). The nuclei of individual cells were identified by DAPI staining, and cells containing two spots per DNA-FISH channel were identified manually. Images were cropped to contain only the identified cell. Both alleles for each DNA locus were defined by applying the Imaris "Surface" function (no smooth and absolute intensity) on the corresponding fluorescent channel. The coordinates of mass center were used to calculate distances between spots in different channels, and the shorter distance from one spot to two alleles of another genomic locus was collected. For the regions *g* and *q*, we measured the absolute volume of individual FISH foci by Imaris. For smaller genomic segments (*e, F, H* and *R*; 100-kb in length*),* the voxels captured by confocal microscopy are too few to get an accurate quantification of its volume. Thus, we only showed changes in the volume of the FISH signal for two ~1-Mb L1-rich (*g, q*) regions on chr17. DNA FISH images were quantified by Volocity[17]. Pearson's correlation coefficients between B1 and L1 labeled channels within nuclei were quantified by Volocity[17]. They were normalized to background signals obtained from five randomly chosen areas outside the nucleus. Quantification of RNA FISH data was done with ImageJ. The total intensity within the nucleus of each z section was measured. The intense cytoplasmic fluorescence was subtracted as background to prevent it affecting the quantification of nuclear fluorescence intensity.

mESC culture, synchronization and transcriptional inhibition

mESCs (J1)[21] were maintained in complete ESC culture medium: DMEM supplemented with 15% heat-inactivated FCS (fetal calf serum), 1% of nucleoside mix (100x stock, Millipore), Penicillin-Streptomycin Solution (100x stock, Life Technologies), 2 mM Glutamax (100x, Life Technology), 0.1 mM non-essential amino acid, 0.1 mM 2-mercaptoethanol and supplied with 1000 U/ml recombinant leukemia inhibitory factor (LIF, Millipore). Cells were maintained at 37 ℃ and 5% CO_2 in a humidified incubator. Auxin-induced degradation (AID) of CTCF and RAD21 was performed as described previously[14,22]. For cell-cycle synchronization, mitotic cells were collected after being treated with 1.25 mM Thymidine for 14 hours and then 50 ng/ml Nocodazole for 7 hours. G1 and S phase cells were fixed at 1.5 hours and 7 hours after release from Nocodazole treatment. Inhibition of transcription in mESCs were performed by treating cells with 100 µM of DRB for 3 hours to block elongation of RNA Pol II or 1 µg/ml of ActD for 3 hours to inhibit both RNA Pol I and II. The control treatment was 0.2% DMSO for 3 hours.

Embryo collection, culture, and microinjection.

Embryos were isolated from 3- to 4-week-old C57BL/6J females mated with C57BL/6J males. To induce ovulation, females were administered with 10 IU of PMSG intraperitoneally and then 10 IU of hCG (San-Sheng pharmaceutical Co. Ltd) 44–48 hours after PMSG. Zygotes for ASO, AMO and mRNA injections were collected 10-12 hours post coitus (hpc) in M2 medium (Sigma M7167) and subsequently cultured in KSOM medium (Merck Millipore MR-106-D). Mouse preimplantation embryos at particular stages were collected *in vitro* at defined time periods after coitus: 21 hpc (PN5 zygote), 21.5 hpc (early 2-cell), 44 hpc (late 2-cell), 56 hpc (4-cell), 63.5 hpc (8-cell), 3.5 dpc (morula) and 4.5 dpc (blastocyst). Microinjection of ASO (5 µM) and AMO (1 mM) into PN3 zygotes was performed on a Leica DMI3000B microscope equipped with a Leica

micromanipulator.

RNA depletion by AMO or ASO

To deplete L1 RNA in mouse embryos and in mESCs, we used the same morpholino antisense oligonucleotide (AMO or ASO) as Percharde *et al.* previously used[23]. This sequence targets the highly conserved inter-ORF region of at least 500 full-length young L1 elements (Fig. 2a) and does not share homologies to any known Refseq mRNAs in mouse. Because of its morpholino chemical backbone, we think it is more appropriate to call this short 25-nt oligo as L1 AMO, rather than L1 ASO as previously designated in the Percharde paper[23]. AMO acts through steric blockage of target RNA, whereas ASO induces RNase H-dependent degradation[24,25]. In both embryos and ESCs, L1 AMO led to 17.4% to 28.3% decreases in L1 RNA levels (Extended Data Fig. 8a, f), which are consistent with the acting mechanism of AMO to inhibit its target RNA's function rather than inducing its degradation. L1 AMO (or L1 ASO) has proven to be effective in inhibiting L1 RNA function in our previous reports[23,26] and also in this work. We used a standard scramble (SCR) AMO that targets a human beta-globin intron mutation (Gene Tools) as the negative control in this study. L1 and SCR AMOs were synthesized and purchased from Gene Tools.

To deplete B1 RNA, two antisense oligonucleotide (ASO) sequences targeting the B1 consensus sequence were designed and synthesized by IDT (Integrated DNA technologies). These ASOs are 20mers linked by phosphorothioate bond, with five 2'-O-methoxyethyl (MOE) RNA bases at both 5' and 3' end, separated by 10 unmodified deoxynucleotides within the central position which is responsible for inducing RNA degradation via RNase H. A standard scramble (SCR) ASO[27] was used as a control for B1 ASO in embryonic injection. All AMO and ASO probes were terminally labeled by fluorescence for selection of positively injected embryos or transfected cells. Their sequences are listed in Supplementary information, Table S4.

We performed L1 RNA depletion as previously described[23]. Briefly, mESCs were transfected

with a Nucleofector electroporation device (Amaxa). SCR and L1 AMOs were used at a concentration of 5 nmol/100 μl for ~2.5×10^6 cells in suspension. After transfection, cells were immediately plated in mESC culture medium. AMO-transfected mESCs were purified by FACS to enrich for successfully nucleofected cells at the 12-hour time-point after transfection. Enriched mESCs were directly used for DNA FISH (12-hour time point) or cultured for additional 24 hours and then harvested for DNA FISH and Hi-C (36-hour time point). The L1 and scramble AMO sequences were the same as those used to treat embryos. We want to note that treatment of mESCs with B1 ASOs led to severe cell death within hours of transfection, precluding direct assessment of the role of B1 transcripts in chromatin organization. This observation is consistent with broad functional roles of B1/Alu repeats in diverse processes, including transcription, RNA processing, and nuclear export[28-31].

Hi-C library generation and sequencing

Two independent experiments of L1 or scramble (SCR) AMO transfection followed by Hi-C were performed. The procedure for small-scale *in situ* Hi-C (sisHi-C) was conducted as previously described[32]. sisHi-C can generate high-quality Hi-C data using only 500 cells that accurately recapitulate chromatin interaction patterns derived from millions of cells. Briefly, mESCs were fixed with 1% formaldehyde at room temperature for 10 minutes. Formaldehyde was quenched with glycine for 10 minutes at room temperature. mESCs were then washed twice with 1 ×PBS. The exchange of buffers was done by transferring mESCs with a mouth capillary pipette. mESCs were lysed in 50 μl lysis buffer (10 mM Tris-HCl pH 7.4, 10 mM NaCl, 0.1 mM EDTA, 0.5% NP-40 and proteinase inhibitor) on ice for 50 minutes. After spinning at 3,000 r.p.m. at 4 ℃ for 5 minutes, the supernatant was discarded carefully with a pipette. Chromatin was solubilized in 10 μl 0.5% SDS and incubated at 62°C for 10 minutes. SDS was quenched with 5 μl 10% Triton X-100 at 37 ℃ for 30 minutes. Then the nuclei were digested with 50 U MboI at 37 ℃ overnight with

rotation in a total volume of 50 μl. MboI was then inactivated at 62°C for 20 minutes. To add the biotin to the DNA, 1.5 μl 1 mM dATP, 1.5 μl 1 mM dGTP, 1.5 μl 1 mM dTTP, 3.75 μl 0.4 mM biotin-14-dCTP and 10 U Klenow were added to the solution and the reaction was carried out at 37°C for 1.5 h with rotation. After adding 60 μl ligation mix (38.8 μl water, 12 μl 10 × NEB T4 DNA ligase buffer, 7 μl 10% Triton X-100, 1.2 μl 10mg/ml BSA and 1 μl 400U/μl T4 DNA ligase), the fragments were ligated at room temperature for 6 h with rotation. This was followed by reversal of crosslinking and DNA purification. DNA was sheared to 300–500 bp with a Covaris M220 ultrasonicator. The biotin- labelled DNA was then pulled down with 10 μl Dynabeads MyOne Streptavidin C1 (Life Technology). Sequencing library preparation was performed on the beads, including end repair, dATP tailing and adaptor ligation. DNA was eluted twice by adding 20 μl water to the tube and incubating at 66 ℃ for 20 minutes. 12–15 cycles of PCR amplification were performed with Extaq (Takara). Finally, size selection was done with AMPure XP beads and fragments ranging from 200 bp to 1,000 bp were selected. All the libraries were sequenced on Illumina HiSeq2500 or HiSeq XTen according to the manufacturer's instruction. Hi-C Summary statistics for Quality control were shown in Supplementary information, Table S5. Please see above for Hi-C data analysis.

H3K9me3 ChIP-seq

Cells were harvested by trypsin digestion and rinsed twice by PBS. Then cells were crosslinked using 1% FMA with rotation at RT for 10 min and stopped by addition of 125 mM glycine. Cells were washed with PBS twice and then treated with 200 μl lysis buffer (50 mM Tris-HCl pH 8.1, 10 mM EDTA, 1% SDS, supplied with 1 mM DTT and 1 mM PMSF) on ice for 10 min. Sonication was performed on ice with 10 s on, 20 s off for 5 cycles with 25% amplification. Samples were spun down at 14,000 rpm at 4℃ for 10 min, and supernatant was collected for immunoprecipitation. For each ChIP, 1 μl H3K9me3 antibody (ABclonal A2360) was added into the sample and

incubated at 4℃ overnight, after washing subsequently with Low Salt IP Wash Buffer (25 mM Tris-HCl pH 8.1, 150 mM NaCl, 1 mM EDTA, 1% Triton X-100, 0.1% SDS, 0.1% Na-deoxycholate), High Salt IP Wash Buffer (25 mM Tris-HCl pH 8.1, 500 mM NaCl, 1 mM EDTA, 1% Triton X-100, 0.1% SDS, 0.1% Na-deoxycholate), LiCl IP Wash Buffer (10 mM Tris-HCl pH 8.1, 250 mM LiCl, 1 mM EDTA, 0.5% NP-40, 0.1% Na-deoxycholate) and TE buffer (10 mM Tris-HCl pH 8.1, 1 mM EDTA) for 10 min at RT of each wash, ChIPed DNA were eluated twice by adding IP Elution solution (50 mM Tris-HCl pH 8.1, 1% SDS, 1 mM EDTA and supplied with 100 mM NaHCO3, 250 mM NaCl, 80 μg/ml Proteinase K) and treated at 65℃ for 20 min. Combined DNA eluates were subjected to reverse crosslinking at 65℃ for 6-16 h, and DNA were extracted by AMPure XP beads (Beckman Coulter, A63881). ChIP-seq library was constructed by NEBNext ChIP-Seq Library Prep Reagent Set and sequenced by Illumina Hiseq 2500 platform of Genewiz.

Heterochromatin fractionation and RNA enrichment analysis

About 3×10^7 mESCs were used for isolation of nuclear extracts and chromatin fractionation based on previously reported method with modifications[33,34]. Briefly, cells were UV crosslinked (energy mode: 254 nm UV, 400 mJ/cm^2) in plate and harvested by trypsin digestion. And cell pellets were resuspended in 200 μl cold cytoplasmic lysis buffer (0.035% NP-40, 10 mM Tris-HCl pH 7.5, 150 mM NaCl, freshly added with 1 mM DTT, 1 mM PMSF, proteinase inhibitor and RNase inhibitor) and incubated on ice for 5 min. The cell lysates were gently layered on the 500 μl cold sucrose buffer (10 mM Tris-HCl pH 7.5, 150 mM NaCl, 24% sucrose w/v, freshly added with 1 mM DTT, 1 mM PMSF, proteinase inhibitor and RNase inhibitor) and centrifuged at 13,000 rpm 4℃ for 10 minutes. After removal of cytoplasmic supernatants, the nuclear pellets were then gently resuspended in 900 μl cold nuclear wash buffer (20 mM HEPES, pH 8.0, 200 mM NaCl, 1 mM EDTA, 0.5 mM EGTA, freshly added with 1 mM DTT, 1 mM PMSF, proteinase inhibitor and RNase inhibitor) and rotated at 4℃ for 10 min. Nuclei were spun down at 1,350 g at 4℃ for 5

minutes and resuspended in 200 μl cold sonication lysis buffer (10 mM Tris-HCl pH 8.0, 100 mM NaCl, 1 mM EDTA, 0.5 mM EGTA, 0.5% N-lauroylsarcosine, 0.1% sodium deoxycholate, freshly added with 1 mM DTT, 1 mM PMSF, proteinase inhibitor and RNase inhibitor) and kept on ice. The sonication was performed with 10 seconds on / 20 seconds off, 25% amplification for 1.5 minutes. After sonication, the lysates were supplied with 1% Triton X-100 and centrifuged at 20,000 g 4℃ for 10 minutes to pellet the debris. The supernatants which represent the nuclear extracts were subjected to subsequent sucrose gradient centrifugation. And 6%-40% linear sucrose gradient was prepared in sucrose basic buffer (10 mM Tris-HCl, pH 8.0, 100 mM NaCl, 1 mM EDTA, 0.5 mM EGTA, 1% Triton-X 100, 0.1% N-lauroylsarcosine, freshly added with 1 mM DTT, 1 mM PMSF, proteinase inhibitor and RNase inhibitor) into 15 ml Polyallomer Centrifuge Tubes (Backman Coulter, 31372). Nuclear extracts were laid on the top of sucrose gradient and centrifuged in a SW41Ti rotor (Backman Coulter) at 40,000 rpm 4℃ for 3 hours. After the centrifugation, fractions were collected and 10% was used for western blot analysis to evaluate the efficiency of fractionation and the left was used for RNA extraction and RT-qPCR analysis.

RNA was extracted using TRIzol reagent (Invitrogen) according to the manufacturer's instructions. First strand cDNA was synthesized using PrimeScript™ RT reagent Kit with gDNA Eraser (TaKaRa, RR047A). Quantitative real-time PCR (RT-qPCR) was performed using 2×RealStar Power SYBR Mixture (Genstar, A311-01) and detected by Bio-Rad CFX384 Real Time System. In Fig. 7d, the RNA enrichment in each fraction was calculated based on the Ct value compared to the nuclear input. Data represented by %Input were shown as mean and standard deviation from two biological replicates. In Extended Data Fig. 7c, relative RNA levels of POU5F1 were first normalized to *CCND3* mRNA (a relatively stable transcript[35]) and then compared to DMSO control. Primers for RT-qPCR analysis are listed in Supplementary information, Table S4.

***In vitro* pull-down assay**

We purified the recombinant human HP1α protein as previously described[36]. We used the full-length L1 DNA cloned from mESC genomic DNA as the template to generate a series of eight 1-kb fragments, designated as F1 to F8, to cover the full-length 6,544-kb L1 sequence by PCR (Extended Data Fig 12a). Their GC contents range from 37.3% to 56.3%. We also synthesized two artificial fragments comprising eight tandem copies of either B1 element (8xB1, in 1-kb length) or scrambled B1 sequence (8xSCR, in 1-kb length) for comparison. Note that a tandem repeat of 8 B1 or scramble sequence might provide a multivalency to the binding and phase separation of HP1α. The templates for *in vitro* transcription were generated by PCR with a forward primer containing the T7 promoter.

T7 promoter-containing DNA fragments were used to generate L1 and control RNA fragments through *in vitro* transcription. Biotinylated DNA were produced by PCR using 5'-biotin modified primers. Purified DNA or RNA fragments of L1 were combined together (designated as L1 mix) for RNA or DNA pull-down and phase separation assays. Primers used for cloning as listed in Supplementary information, Table S1.

RNA was obtained by *in vitro* transcription and purified according to the manufacturer's protocol (Ambion, AM1334). Synthesis of biotinylated RNA fragments was carried out by *in vitro* transcription in the presence of biotin-16-rUTP (Roche, 11388908910). After incubation at 37℃ for 16 hours, the reaction was stopped by adding the ammonium acetate Stop Solution and DNA template was digested by TURBO DNase. *In vitro* transcribed RNA was then extracted by TRIzol™ Reagent (Invitrogen) followed by ethanol precipitation. The quality of DNA and RNA were confirmed by Nanodrop™ 2000 spectrophotometry and agarose gel.

For the pull-down assay, 1 μg of biotin-labeled RNA was denatured at 65℃ for 5 minutes, then snap-cooled on ice for 2 minutes. Finally, RNA was refolded by adding the structure buffer (final concentration: 10 mM Tris-HCl, pH 7.4, 150 mM NaCl, 10 mM $MgCl_2$) and stand at room temperature for 20 minutes. Refolded control or L1 RNA (a mixture of 8 L1 RNA fragments) and

2 μg of HP1α protein were incubated with pre-blocked streptavidin M-280 beads (Invitrogen) in IP buffer (25 mM Tris-HCl pH 7.4, 1 mM $MgCl_2$, 150 mM NaCl, 0.5% Triton X-100, 5% glycerol) at room temperature for 30 minutes with rotation. After incubation, the mixture was washed three times for 10 minutes at 4℃ with rotation by using 1 ml of washing buffer containing various concentrations of salt (0.075/0.15/0.3/0.5/1 M NaCl or 1 M NaCl + 1 M Urea in 20 mM HEPES pH 7.4, 10 mM KCl, 1.5 mM $MgCl_2$, 1 mM EDTA, 5% glycerol). Finally, the beads were resuspended in 40 μl elution buffer (10 mM Tris-HCl, pH 8.0, 1 mM EDTA, 1% SDS) and rotated for 30 minutes at 16℃. Eluate was heated in sample buffer (50 mM Tris-HCl pH 6.8, 2% SDS, 0.1% bromophenol blue, 10% glycerol, 1% β-mercaptoethanol) at 95℃ for 10 minutes then analyzed by coomassie brilliant blue staining or western blot using antibody against HP1α (ab77256).

***In vitro* phase separation assays**

Recombinant HP1α protein and DNA and RNA fragments for L1, 8xB1, and 8xSCR were prepared as described above. *In vitro* phase separation assays were performed in a 384-well low-binding 0.17 mm microscopy plate (In Vitro Scientific) and samples were mixed directly on the glass bottom. The samples were observed after incubation at 4℃ overnight. Phase separation assays were done in the reaction buffer (50 mM NaCl, 10 mM HEPES pH 7.4, 0.5 mM DTT) with various concentrations of HP1α protein, DNA and RNA. Phase separation was tested at HP1α protein concentrations ranging from 6.25-50 μM, DNA and RNA concentrations ranging from 0-200 ng/μl. In order to have a clear view of phase separation and also to quantify the droplets formed *in vitro*, we used fluorescence to label HP1α and stained RNA/DNA with propidium iodide. Alexa Fluor™ 488 NHS Ester (Invitrogen, A20000) was mixed with HP1α protein and incubated on ice for at least 2 hours avoiding light exposure, which provides a selectively covalent link between

Alexa Fluor dye and primary amines (R-NH2) located on proteins. Fluorescence-labeled HP1α protein was mixed with unlabeled HP1α (labeled:unlabeled = 1:19) protein before use.

In vitro FRAP experiments were performed using 50 iterations with 100% transmission of a 488 nm laser in an UltraVIEW VoX spinning disc microscope (PerkinElmer). To reduce the photobleaching during image acquisition, we used low transmission of 561 nm or 647 nm lasers. In one field, three rectangular bleached regions were chosen on different droplets. Recovery from photobleaching was recorded for the indicated time. Images were processed and analyzed by Fiji (Fiji Is Just ImageJ, software available at https://imagej.net/Fiji/Downloads).

Quantification and statistical analysis

Statistical analyses were carried out using Excel or R (version 3.4.3). All of the statistical details can be found in the relevant figure legends.

References

1 Tempel, S. Using and understanding RepeatMasker. *Methods Mol Biol* **859**, 29-51, doi:10.1007/978-1-61779-603-6_2 (2012).

2 Kent, W. J. *et al.* The human genome browser at UCSC. *Genome Res* **12**, 996-1006, doi:10.1101/gr.229102 (2002).

3 Kim, D. *et al.* TopHat2: accurate alignment of transcriptomes in the presence of insertions, deletions and gene fusions. *Genome Biol* **14**, R36, doi:10.1186/gb-2013-14-4-r36 (2013).

4 Trapnell, C. *et al.* Differential gene and transcript expression analysis of RNA-seq experiments with TopHat and Cufflinks. *Nat Protoc* **7**, 562-578, doi:10.1038/nprot.2012.016 (2012).

5 Langmead, B. & Salzberg, S. L. Fast gapped-read alignment with Bowtie 2. *Nat Methods* **9**, 357-

359, doi:10.1038/nmeth.1923 (2012).

6 Zhang, Y. *et al.* Model-based analysis of ChIP-Seq (MACS). *Genome Biol* **9**, R137, doi:10.1186/gb-2008-9-9-r137 (2008).

7 Servant, N. *et al.* HiC-Pro: an optimized and flexible pipeline for Hi-C data processing. *Genome Biol* **16**, 259, doi:10.1186/s13059-015-0831-x (2015).

8 Imakaev, M. *et al.* Iterative correction of Hi-C data reveals hallmarks of chromosome organization. *Nat Methods* **9**, 999-1003, doi:10.1038/nmeth.2148 (2012).

9 Akdemir, K. C. & Chin, L. HiCPlotter integrates genomic data with interaction matrices. *Genome Biol* **16**, 198, doi:10.1186/s13059-015-0767-1 (2015).

10 Wang, Y. *et al.* The 3D Genome Browser: a web-based browser for visualizing 3D genome organization and long-range chromatin interactions. *Genome Biol* **19**, 151, doi:10.1186/s13059-018-1519-9 (2018).

11 Lieberman-Aiden, E. *et al.* Comprehensive mapping of long-range interactions reveals folding principles of the human genome. *Science* **326**, 289-293, doi:10.1126/science.1181369 (2009).

12 Fraser, J. *et al.* Hierarchical folding and reorganization of chromosomes are linked to transcriptional changes in cellular differentiation. *Mol Syst Biol* **11**, 852, doi:10.15252/msb.20156492 (2015).

13 Schmitt, A. D. *et al.* A Compendium of Chromatin Contact Maps Reveals Spatially Active Regions in the Human Genome. *Cell Rep* **17**, 2042-2059, doi:10.1016/j.celrep.2016.10.061 (2016).

14 Nora, E. P. *et al.* Targeted Degradation of CTCF Decouples Local Insulation of Chromosome Domains from Genomic Compartmentalization. *Cell* **169**, 930-944, doi:10.1016/j.cell.2017.05.004 (2017).

15 Falk, M. *et al.* Heterochromatin drives compartmentalization of inverted and conventional nuclei.

Nature **570**, 395-399, doi:10.1038/s41586-019-1275-3 (2019).

16 Abramo, K. *et al.* A chromosome folding intermediate at the condensin-to-cohesin transition during telophase. *Nat Cell Biol* **21**, 1393-1402, doi:10.1038/s41556-019-0406-2 (2019).

17 Barlow, A. L., Macleod, A., Noppen, S., Sanderson, J. & Guerin, C. J. Colocalization analysis in fluorescence micrographs: verification of a more accurate calculation of pearson's correlation coefficient. *Microsc Microanal* **16**, 710-724, doi:10.1017/S143192761009389X (2010).

18 Wang, S. *et al.* Spatial organization of chromatin domains and compartments in single chromosomes. *Science* **353**, 598-602, doi:10.1126/science.aaf8084 (2016).

19 Quinodoz, S. A. *et al.* Higher-Order Inter-chromosomal Hubs Shape 3D Genome Organization in the Nucleus. *Cell* **174**, 744-757 e724, doi:10.1016/j.cell.2018.05.024 (2018).

20 Tsanov, N. *et al.* smiFISH and FISH-quant–a flexible single RNA detection approach with super-resolution capability. *Nucleic acids research* **44**, e165-e165 (2016).

21 Shen, X. *et al.* Jumonji modulates polycomb activity and self-renewal versus differentiation of stem cells. *Cell* **139**, 1303-1314, doi:10.1016/j.cell.2009.12.003 (2009).

22 Rhodes, J. *et al.* Cohesin disrupts polycomb-dependent chromosome interactions. 593970, doi:10.1101/593970 %J bioRxiv (2019).

23 Percharde, M. *et al.* A LINE1-Nucleolin Partnership Regulates Early Development and ESC Identity. *Cell* **174**, 391-405 e319, doi:10.1016/j.cell.2018.05.043 (2018).

24 Abrams, D. R. C. a. J. M. Morpholino antisense oligonucleotides: tools for investigating vertebrate development. *Genome Biol.* **2**, reviews1015.1011–reviews1015.1013., doi:10.1186/gb-2001-2-5-reviews1015 (2001).

25 Heasman, J. Morpholino oligos: making sense of antisense? *Dev Biol* **243**, 209-214,

doi:10.1006/dbio.2001.0565 (2002).

26 Lu, J. Y. *et al.* Genomic Repeats Categorize Genes with Distinct Functions for Orchestrated Regulation. *Cell Rep* **30**, 3296-3311 e3295, doi:10.1016/j.celrep.2020.02.048 (2020).

27 Wheeler, T. M. *et al.* Targeting nuclear RNA for in vivo correction of myotonic dystrophy. *Nature* **488**, 111-115, doi:10.1038/nature11362 (2012).

28 Chen, C., Ara, T. & Gautheret, D. Using Alu elements as polyadenylation sites: A case of retroposon exaptation. *Mol Biol Evol* **26**, 327-334, doi:10.1093/molbev/msn249 (2009).

29 Dominissini, D., Moshitch-Moshkovitz, S., Amariglio, N. & Rechavi, G. Adenosine-to-inosine RNA editing meets cancer. *Carcinogenesis* **32**, 1569-1577, doi:10.1093/carcin/bgr124 (2011).

30 Lubelsky, Y. & Ulitsky, I. Sequences enriched in Alu repeats drive nuclear localization of long RNAs in human cells. *Nature* **555**, 107-111, doi:10.1038/nature25757 (2018).

31 Polak, P. & Domany, E. Alu elements contain many binding sites for transcription factors and may play a role in regulation of developmental processes. *Bmc Genomics* **7**, 133, doi:10.1186/1471-2164-7-133 (2006).

32 Du, Z. *et al.* Allelic reprogramming of 3D chromatin architecture during early mammalian development. *Nature* **547**, 232-235, doi:10.1038/nature23263 (2017).

33 Becker, J. S. *et al.* Genomic and Proteomic Resolution of Heterochromatin and Its Restriction of Alternate Fate Genes. *Mol Cell* **68**, 1023-1037 e1015, doi:10.1016/j.molcel.2017.11.030 (2017).

34 Bhatt, D. M. *et al.* Transcript dynamics of proinflammatory genes revealed by sequence analysis of subcellular RNA fractions. *Cell* **150**, 279-290, doi:10.1016/j.cell.2012.05.043 (2012).

35 Sharova, L. V. *et al.* Database for mRNA half-life of 19 977 genes obtained by DNA microarray analysis of pluripotent and differentiating mouse embryonic stem cells. *DNA Res* **16**, 45-58,

doi:10.1093/dnares/dsn030 (2009).

36 Wang, L. *et al.* Histone Modifications Regulate Chromatin Compartmentalization by Contributing to a Phase Separation Mechanism. *Mol Cell*, doi:10.1016/j.molcel.2019.08.019 (2019).
